# Carbon Nanotube-Based Gas Sensors: Sensing Mechanisms, Functional Interfaces, Gas-Specific Performance, and Flexible/Wearable Integration

**DOI:** 10.3390/s26154959

**Published:** 2026-08-05

**Authors:** Daewoong Jung

**Affiliations:** 1 Department of Nanomechatronics Engineering, College of Nanoscience & Nanotechnology, Pusan National University, 2 Busandaehak-ro, Busan 46241, Republic of Korea; dwjung@pusan.ac.kr; 2 School of Transdisciplinary Engineering, College of Engineering, Pusan National University, 2 Busandaehak-ro, Busan 46241, Republic of Korea

**Keywords:** carbon nanotubes, gas sensors, chemiresistor, functionalization, metal oxide, conducting polymer, flexible electronics, room-temperature sensing

## Abstract

Carbon nanotubes (CNTs) have become one of the most widely investigated nanomaterials for gas sensing because their nearly one-dimensional geometry, large surface-to-volume ratio, hollow structure, and tunable metallic or semiconducting character allow trace adsorption events to be transduced into measurable electrical signals at or near room temperature. This review summarizes CNT-based gas sensors from a system-oriented perspective, linking four interconnected topics: (i) CNT structure, synthesis, and film/device fabrication; (ii) sensing mechanisms, including charge transfer, Schottky-barrier modulation, carrier-lifetime effects, and field-enhanced ionization; (iii) functional interfaces based on noble metals, metal oxides, conducting polymers, and graphene derivatives; and (iv) gas-specific and flexible/wearable device performance. Particular attention is given to recent room-temperature and mechanically compliant CNT-film sensors fabricated on polymer, cellulose, paper, textile, and mask substrates. Rather than cataloguing only individual response values, this review compares representative devices in terms of target gas, operating condition, sensitivity, recovery strategy, selectivity, humidity tolerance, and wearable relevance. The review concludes by discussing remaining challenges in reproducibility, selectivity, humidity compensation, recovery, power consumption, and standardization, and by outlining future directions toward robust, scalable, and intelligent CNT-enabled sensing systems.

## 1. Introduction

Reliable detection of hazardous, toxic, and greenhouse gases is essential for industrial safety, environmental monitoring, space exploration, biomedicine, and homeland security [1,2]. Pollutants and greenhouse gases such as CO, CO_2_, CH_4_, N_2_O, SO_2_, NO_x_, and NH_3_ have accumulated in the atmosphere largely as a result of human activity, while indoor leakage of combustible or toxic species poses acute risks to health. An effective gas-sensing system is generally required to combine high sensitivity and selectivity, fast response and recovery, low analyte consumption, low operating temperature, and stable long-term performance [1]. Conventional gas-sensing materials—vapor-sensitive polymers, semiconductor metal oxides, and porous structures—satisfy some of these requirements but frequently demand elevated operating temperatures or offer limited form-factor flexibility [1,2,3].

The advent of nanotechnology has reshaped this landscape by providing materials whose extremely high surface-to-volume ratio and nanoscale dimensions are ideally suited to the adsorption and detection of gas molecules. Among such nanomaterials, carbon nanotubes (CNTs) have attracted sustained interest since their identification by Iijima in 1991 [4]. A CNT can be regarded as a seamless cylinder formed by rolling up one or more graphene sheets: single-walled carbon nanotubes (SWCNTs) consist of a single graphene wall, whereas multi-walled carbon nanotubes (MWCNTs) comprise several concentric tubes sharing a common axis [5]. This structure endows CNTs with outstanding electrical conductivity, mechanical strength, thermal stability, and—crucially for sensing—a surface in which essentially every atom is exposed to the environment. Consequently, adsorption of even a small quantity of gas can perturb the carrier concentration or transport properties of the tube enough to be transduced into an electrical signal [6]. For these reasons, CNT-based gas sensors and their operating mechanisms have been the subject of intensive experimental and theoretical study over the past two decades [7,8,9,10].

Despite these strengths, pristine CNTs are rarely sufficient for practical gas analysis. Many gases induce similar resistance changes because the basic transduction mechanism is often a nonspecific charge transfer; strongly adsorbed species can slow recovery and generate baseline drift, while weakly adsorbed molecules may produce only low signals. Reproducibility is further complicated by CNT chirality, metallic/semiconducting composition, defect density, residual catalyst, bundle structure, network density, and substrate effects. Consequently, the field has shifted from pristine CNT networks toward materials-by-design strategies that combine CNTs with noble metals, transition metals, metal oxides, conducting polymers, graphene derivatives, transition-metal dichalcogenides (TMDCs), metal–organic frameworks (MOFs), and biomolecular receptors [7,8,9,10].

The need for an updated review arises from the rapid broadening of the field and from the increasing emphasis on practical, flexible, and low-power sensing platforms. Previous reviews have discussed CNT gas-sensor fundamentals, CNT chemiresistors, ammonia sensing by carbon nanomaterials, flexible NO_2_/NH_3_ sensors, carbon-nanomaterial trends, and metal/metal-oxide/polymer modification strategies [8,10,11,12,13].

Although these reviews have provided valuable foundations for CNT-based gas sensors, several gaps remain. Many previous reviews emphasize CNT synthesis, intrinsic electrical properties, individual target gases, or specific functionalization routes, but comparatively fewer studies integrate sensing mechanisms with the device architecture, performance metrics, humidity interference, recovery protocol, reproducibility, and system-level implementation within a single framework. In addition, response values are often compared across studies without sufficient attention to differences in response definition, gas concentration, relative humidity, flow or chamber conditions, recovery method, number of tested devices, and long-term drift. As a result, it remains difficult to determine whether high reported responses originate from intrinsic CNT–gas interactions, functional-interface effects, device geometry, or testing protocol differences. The present review addresses this remaining gap by treating CNT gas sensors not as sensing materials, but as complete sensing systems in which material chemistry, transduction mechanism, device configuration, testing protocol, and practical deployment are interdependent.

The novelty of this review is therefore fivefold. First, it does not treat CNT gas sensors only as individual material-response case studies, but organizes them according to the mechanistic pathway that dominates the response, including charge transfer, Schottky-barrier modulation, heterojunction effects, catalytic spillover, tunneling/percolation modulation, molecular sieving, and recovery-assisted transduction. Second, it explicitly links functionalization strategies with the corresponding sensing mechanisms, showing how noble metals, metal oxides, conducting polymers, graphene derivatives, MOFs, TMDs, and biomolecular receptors alter sensitivity, selectivity, recovery, humidity tolerance, and reproducibility. Third, it connects gas-specific performance with flexible, wearable, self-powered, and system-level integration, thereby moving beyond material-level comparison toward practical deployment. Fourth, unlike many reviews that compare response values without fully addressing reporting inconsistency, this review emphasizes standardized reporting, including response definition, LOD calculation, humidity condition, recovery protocol, device-to-device statistics, long-term drift, durability, and power consumption. Fifth, it highlights emerging system-level directions, including integrated sensing platforms, wireless readout, machine-learning-assisted recognition, sensor arrays, drift compensation, and intelligent environmental-monitoring systems. This structure distinguishes the present review from earlier reviews that focused primarily on CNT fundamentals, individual target gases, chemiresistive response, or isolated modification strategies.

Accordingly, this manuscript is organized around mechanisms, interfaces, and application-oriented design rules rather than only by gas species. The scope is CNT-centered but not CNT-isolated: graphene, reduced graphene oxide (rGO), carbon nanofibers (CNFs), transition-metal dichalcogenides (TMDCs), metal–organic frameworks (MOFs), and metal-oxide nanoparticles are discussed where they form hybrid sensing interfaces with CNTs or provide useful comparisons for CNT device design. Section 2 summarizes the CNT structure, intrinsic properties, synthesis, and film fabrication. Section 3 examines the fundamental sensing mechanisms. Section 4 surveys major device architectures and performance metrics. Section 5 addresses functionalization with noble metals, metal oxides, conducting polymers, and graphene derivatives. Section 6 reviews the gas-specific sensing characteristics of ammonia, nitrogen dioxide, hydrogen, hydrogen sulfide, and carbon dioxide. Section 7 focuses on flexible and wearable CNT-based sensing platforms. Section 8 discusses challenges and future perspectives, and Section 9 concludes.

Moreover, to distinguish paradigm-shifting contributions from incremental case studies, Table 1 summarizes representative seminal advances that shaped the development of CNT-based gas sensors. These studies were selected not simply because they reported high response values, but because they introduced new sensing concepts, device architectures, functional-interface strategies, or system-level directions that influenced later research. This perspective is used throughout the review to evaluate CNT gas sensors in terms of mechanistic significance, practical relevance, and translational potential rather than by response magnitude alone.

## 2. Structure, Properties, and Synthesis of Carbon Nanotubes

### 2.1. Structure and Intrinsic Properties

**Figure 1:**
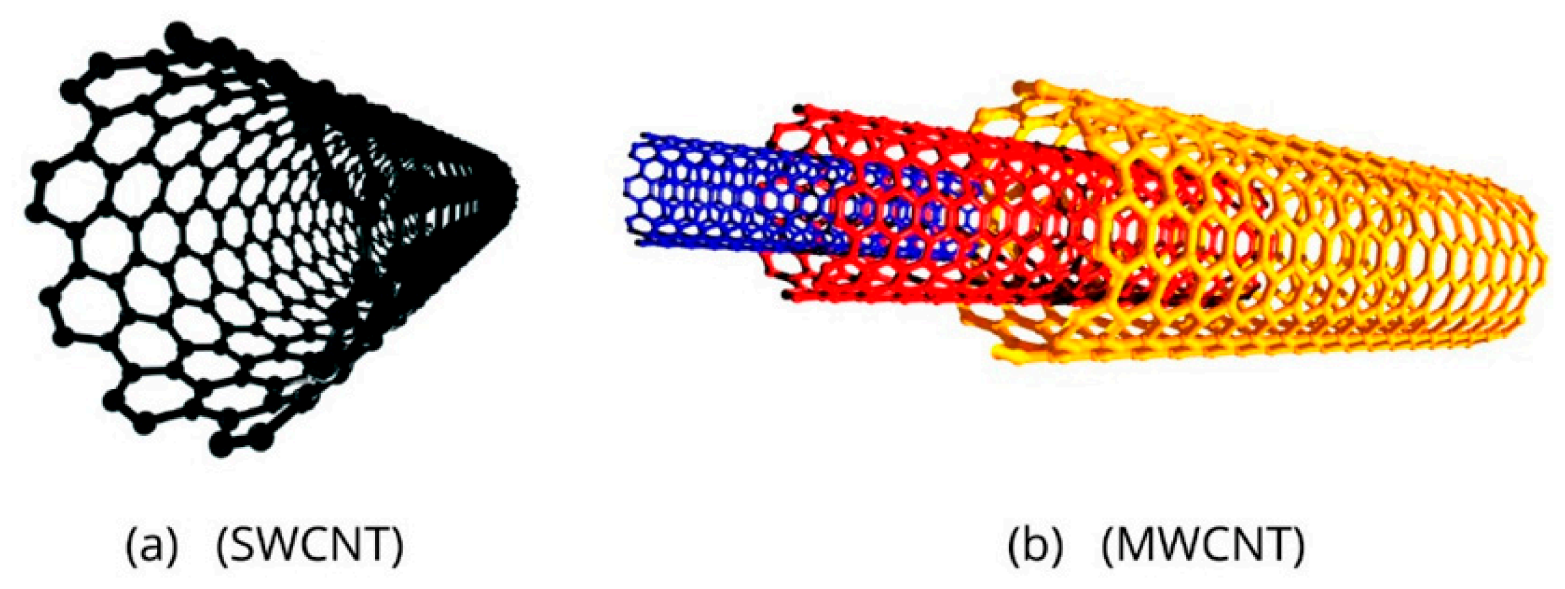
Conceptual diagrams of single-walled carbon nanotubes (SWCNTs) (**a**) and multiwalled carbon nanotubes (MWCNTs) (**b**). All panels adapted with permission from ref. [79].

CNTs belong to the fullerene family and are conveniently classified as SWCNTs or MWCNTs (Figure 1). The diameter of SWCNTs typically ranges from about 0.8 to 2 nm, whereas MWCNTs span roughly 5 to 100 nm, and tube lengths can extend from micrometers to millimeters [75,76]. The electronic character of a CNT is governed by its chirality and diameter: depending on the way the graphene lattice is rolled, expressed by the chiral index (n, m), a nanotube may be metallic or semiconducting, with semiconducting tubes exhibiting a diameter-dependent band gap [77]. This one-dimensional nature reduces electron scattering relative to bulk materials and allows high current densities with little heating [78,79].

**Figure 2:**
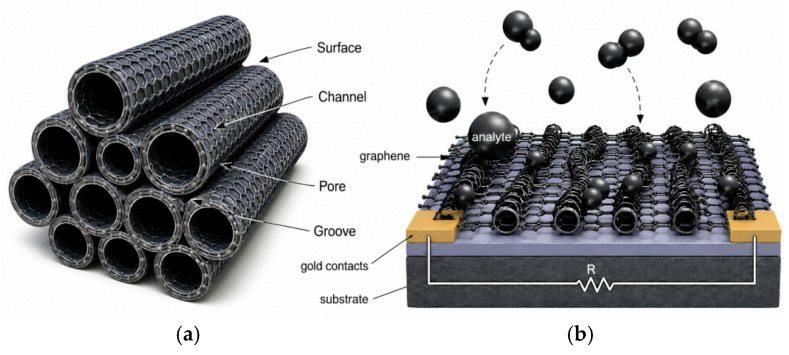
Adsorption environments in carbon nanomaterials. (**a**) A CNT bundle exposes the outer wall surface, the grooves between adjacent tubes, the interstitial channels inside the bundle, and the interior pore of uncapped tubes; (**b**) a graphene sheet adsorbs molecules on both basal faces. Schematic illustration prepared with the assistance of ChatGPT (GPT-5.5)-based AI tools and verified by the author.

The mechanical and transport properties of CNTs are remarkable, with reported tensile strengths in the tens of GPa, a Young’s modulus approaching 1 TPa, electron mobilities exceeding 10^4^ cm^2^ V^−1^ s^−1^, and high thermal conductivity along the tube axis [79,80]. For gas sensing, however, the single most important property is the very large accessible surface area, which renders the local chemical environment of the tube directly observable through its electrical response. In SWCNT bundles, gas molecules can adsorb at several distinct sites—the external surface, the grooves between adjacent tubes, the interstitial channels within a bundle, and, where tubes are uncapped or defective, the interior pore—each characterized by a different binding energy and accessibility (Figure 2) [81]. By comparison, a graphene sheet adsorbs gas molecules on both basal faces; the relative population of these sites, and therefore the overall sensitivity, is governed by the binding energy of the target gas [82].

The gas-sensing advantage of CNTs therefore arises from the combination of a high surface-to-volume ratio and efficient electrical transport. Adsorption at the nanotube wall, at defect sites, at functional groups, at inter-tube junctions, or at metal/CNT contacts can perturb the total device resistance. In network sensors, the measured resistance includes contributions from intrinsic tube resistance, tube–tube contacts, tube–electrode contacts, and percolation geometry. This distributed architecture amplifies small molecular perturbations but also makes response interpretation more complex than in a single-crystal semiconductor [11,12,13,83,84]. CNT surfaces are chemically versatile. Non-covalent functionalization through π–π stacking, van der Waals interactions, polymer wrapping, or electrostatic adsorption can preserve the conjugated electronic structure while introducing selective receptors. Covalent functionalization—oxidation, amination, diazonium chemistry, or cycloaddition—provides stable anchoring sites for nanoparticles and polymers but may disrupt tube conductance if overused. The central design problem is therefore to introduce enough active sites for selectivity without destroying the conductive CNT network [85,86,87].

### 2.2. Synthesis Methods

**Figure 3:**
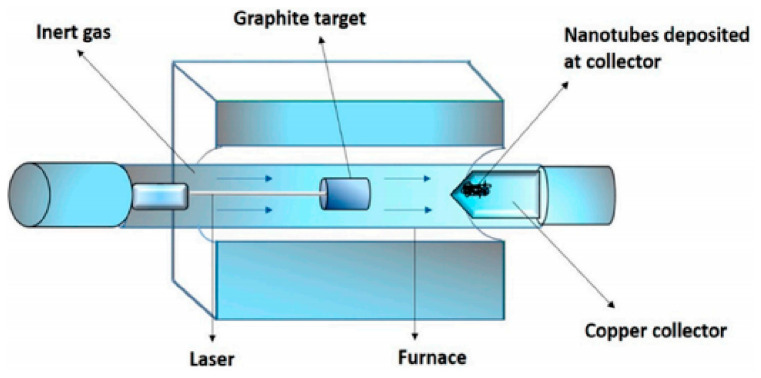
Schematic structure showing the laser ablation method. All panels adapted with permission from ref. [88].

Three techniques dominate CNT production: arc discharge, laser ablation, and chemical vapor deposition (CVD) [88,89,90]. In arc discharge—the oldest method, and the one by which CNTs were first observed—a high voltage struck between two carbon electrodes generates a plasma that sublimes the anode carbon; nanotubes then condense, often aided by metal catalysts and an inert atmosphere. The method yields SWCNTs and MWCNTs of high crystallinity and few defects, but with limited control over morphology [89,90]. Laser ablation (Figure 3), in which intense laser pulses vaporize a graphite–catalyst target inside a heated furnace, similarly produces high-purity material, again at the cost of extreme temperatures and modest scalability [91].

CVD has become the workhorse for large-area and device-oriented synthesis. A hydrocarbon precursor (commonly methane, acetylene, or ethylene) decomposes over catalytic metal nanoparticles such as Fe, Co, Ni, or Mo at moderate temperatures, nucleating nanotubes on a substrate [90,92]. Although CVD-grown MWCNTs tend to contain more defects than arc-discharge material, the technique offers decisive advantages: it operates under comparatively mild conditions, scales readily, and—most importantly—permits structural control, including direct growth of vertically aligned CNT (VACNT) arrays and patterned architectures positioned selectively on a substrate [93]. After growth, purification by oxidation and acid treatment removes amorphous carbon and residual catalyst, since impurities can otherwise mask the intrinsic properties of the nanotubes [94].

The synthesis route also affects gas-sensing behavior because it determines the structural quality, defect density, residual catalyst content, oxygen-containing functional groups, and network morphology of CNTs. Highly crystalline CNTs produced by arc discharge or laser ablation generally provide stable electrical transport and low intrinsic noise, but their relatively inert sidewalls may limit adsorption-site density and reduce sensitivity toward weakly interacting gases unless additional functionalization is introduced. In contrast, CVD-grown CNTs often contain more defects, open ends, residual metal catalysts, and oxygen-containing groups after purification or acid treatment. These sites can enhance gas adsorption, catalytic interaction, and charge transfer, thereby improving response magnitude; however, excessive defects, catalyst residues, or nonuniform oxidation can also increase baseline drift, hysteresis, humidity sensitivity, and device-to-device variation. Residual Fe, Co, Ni, or Mo catalysts may contribute to catalytic dissociation or redox reactions, while carboxyl, hydroxyl, and carbonyl groups formed during oxidative purification can act as anchoring sites for metal nanoparticles, polymers, MOFs, or oxide layers. Therefore, CNT synthesis and post-treatment conditions should be considered as sensing-design variables rather than merely material-preparation steps, and they should be reported together with gas-sensing performance. Table 2 summarizes the major CNT synthesis and post-treatment routes with emphasis on their sensor-relevant effects, including defect density, residual catalyst content, oxygen-containing groups, functionalization compatibility, and reproducibility.

### 2.3. Film and Device Fabrication

**Figure 4:**
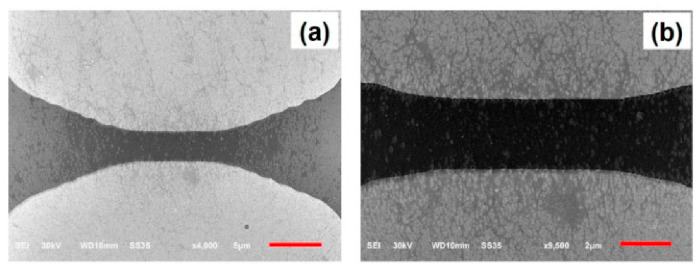
(**a**) SEM image of CNTs aligned and assembled between a pair of electrodes in a CNT-NMP solution with a higher concentration of CNTs using the DEP process; (**b**) enlarged view of the CNTs, showing a network-like profile of CNTs. All panels adapted with permission from ref. [98].

Translating powder or suspension CNTs into a working sensor requires a deposition or assembly step, and CNT quality and device reproducibility depend strongly on this processing. Solution processing—drop-casting, spin-coating, spraying, or vacuum filtration of dispersed CNT inks—is simple and substrate-agnostic but demands careful control of dispersion, frequently using surfactants, sonication, and post-deposition annealing or plasma treatment to remove residues and promote inter-tube contact [95,96]. Printing methods, including screen and inkjet printing, allow patterned chemiresistors and electrodes to be defined directly, with inkjet offering a high resolution and additive, mask-free deposition. Dielectrophoresis (DEP) provides controlled trapping and alignment of CNTs between electrodes and establishes good electrical contact, while direct CVD growth on the sensor substrate yields aligned mats with superior reproducibility (Figure 4) [97,98]. For flexible gas sensors, CNT networks can be deposited on polyimide, polyethylene terephthalate (PET), polydimethylsiloxane (PDMS), paper, textiles, cellulose, and elastomeric substrates [99,100,101]. The choice of fabrication route strongly influences the density, morphology, and ultimately, the response and stability of the resulting device.

## 3. Gas-Sensing Mechanisms

### 3.1. Charge Transfer and Resistance Modulation

**Figure 5:**
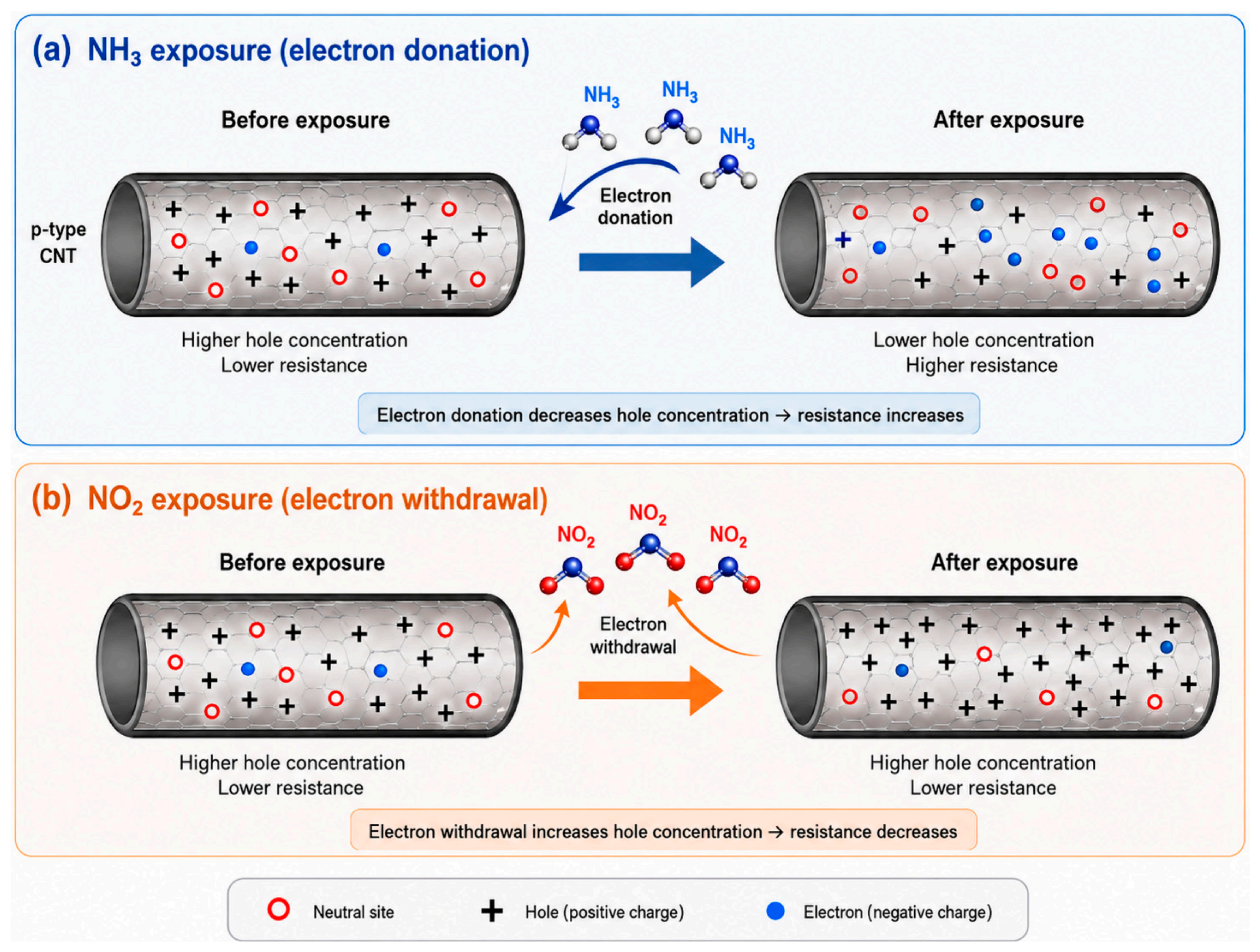
Charge-transfer sensing mechanism for a p-type CNT. (**a**) A reducing gas (NH_3_) donates electrons, neutralizing holes and increasing resistance; (**b**) an oxidizing gas (NO_2_) withdraws electrons, increasing hole density and decreasing resistance. Schematic illustration prepared with the assistance of ChatGPT (GPT-5.5)-based AI tools and verified by the author.

The dominant transduction mechanism in CNT chemiresistors is charge transfer between adsorbed gas molecules and the nanotube. As-prepared CNTs in air generally behave as p-type semiconductors, in part because adsorbed oxygen withdraws electrons and pins the carrier population toward holes [10,11,12,13]. When an oxidizing (electron-withdrawing) gas such as NO_2_ adsorbs, it extracts electrons from the semiconducting tube, increasing the hole concentration and thereby decreasing the resistance; conversely, a reducing (electron-donating) gas such as NH_3_ injects electrons that recombine with holes, lowering the carrier density and increasing the resistance [102,103]. This complementary behavior, illustrated in Figure 5, is the basis of most CNT resistive sensors and was demonstrated in the seminal single-tube transistor experiments of Kong and co-workers, in which exposure to NH_3_ and NO_2_ changed the conductance by orders of magnitude within seconds [14].

The role of functionalization can be understood more clearly when it is mapped onto the sensing mechanisms summarized in Table 3. Noble-metal nanoparticles primarily introduce catalytic dissociation, spillover, and work-function modulation, which are particularly important for weakly interacting gases such as H_2_. Metal-oxide and TMD hybrids create p–n or Schottky-type heterojunctions, depletion layers, oxygen-vacancy-related adsorption sites, and surface-redox pathways. Conducting polymers modify CNT responses through molecular doping/dedoping, acid–base interaction, swelling-induced percolation changes, and tube–tube tunneling modulation. Graphene derivatives, MOFs, peptides, and molecular receptors provide additional adsorption sites, molecular-sieving effects, humidity-screening functions, or selective chemical interactions. Thus, functionalization should not be regarded simply as surface decoration; rather, it defines which transduction pathway dominates the sensor response and determines the balance among sensitivity, selectivity, recovery, humidity tolerance, and reproducibility.

**Figure 6:**
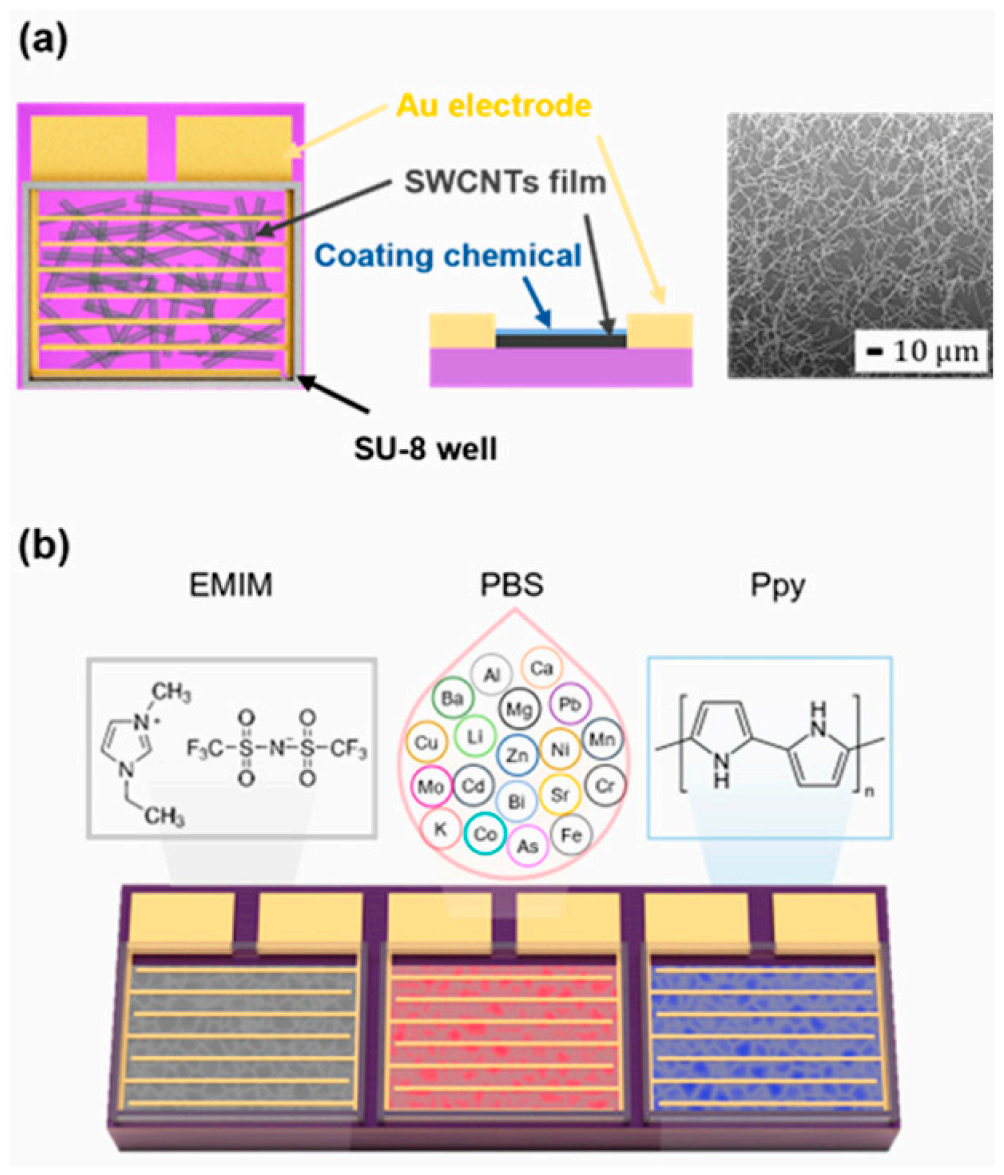
SWCNT sensor array fabrication. (**a**) Design of the SWCNT chemiresistor and SEM images for transferred SWCNT film on SiO_2_ substrate. (**b**) Chemiresistor array coated with EMIM, PBS, and PPy, respectively. All panels adapted with permission from ref. [15].

Beyond the simple donor/acceptor picture, adsorbed analytes can modulate the measured conductance through three distinct routes: (i) modulation of the Schottky barrier at the CNT–electrode contact, (ii) charge transfer along the body of the tube, and (iii) alteration of the inter-tube junctions that link individual nanotubes into a percolating network. Since individual tubes are rarely long enough to bridge the electrodes, the response of a practical network device reflects a combination of all three contributions (Figure 6) [104]. At the metal/CNT interface, adsorption can shift the Schottky barrier height and width and alter carrier injection; on the sidewall, adsorbates shift carrier density or introduce scattering; and at tube–tube junctions, molecules can change tunneling barriers, inter-tube distances, polymer swelling, or the local dielectric environment. Distinguishing these contributions is difficult in a two-terminal chemiresistor but can be approached using FET measurements, contact engineering, controlled network density, and temperature-dependent analysis [105,106].

This multi-region sensitivity explains why CNT networks respond at low analyte concentrations but also why device reproducibility is challenging: a small difference in electrode work function, network alignment, or junction density can change the dominant sensing contribution. For this reason, sensor reports should describe the electrode material, channel geometry, network resistance, and fabrication reproducibility, not only the sensing-material composition. The same effects are observed in field-effect operation, where gate-voltage sweeps separate doping, Schottky-barrier, and mobility contributions to the response.

Charge transfer is not the only operative mechanism. Theoretical NO_2_ studies and molecular-doping experiments underscore the importance of local electronic structure: the conductance of a semiconducting SWCNT depends strongly on the distance between the NO_2_ adsorption site and the metal electrode, with some configurations producing roughly tenfold conductance variation and negative-differential-resistance behavior [107]. Non-covalent amphoteric doping can tune both baseline conductivity and analyte preference: TTF-modified SWCNT films enhance the trace NO_2_ response, whereas TCNQ-modified films favor NH_3_ detection [108,109]. Transport studies have further shown that even inert gases such as N_2_ and He, which exchange essentially no charge with CNTs, can alter the thermopower and resistance of nanotube mats [110]. These effects are attributed to modulation of the carrier lifetime—through additional scattering from adsorbed species or gas-induced phonon generation—rather than to a shift of the Fermi level, an interpretation captured by Nordheim–Gorter analysis [110]. For MWCNTs, the multilayer structure further complicates the response, so that the overall behavior reflects a combination of charge-transfer and scattering contributions.

### 3.2. Adsorption Energetics and Theoretical Insight

First-principles calculations based on density functional theory have clarified why CNTs respond strongly to some gases but not to others. Studies of molecular adsorption on SWCNTs reveal that most common species—H_2_O, NH_3_, CO_2_, CH_4_, H_2_, N_2_, and Ar—are weak charge donors that bind through physisorption with small adsorption energies and negligible charge transfer, whereas NO_2_ and O_2_ are strong charge acceptors with large binding energies, consistent with experiments [111]. This explains the intrinsic sensitivity of pristine CNTs to NO_2_ and their relative insensitivity to many toxic or inert gases. Theory also pointed the way to a remedy: substitutional doping with boron or nitrogen, or the use of composite B_x_CᵧNᴢ nanotubes, modifies the local chemical reactivity and enables strong, even chemisorptive, interactions with otherwise weakly bound molecules such as CO and H_2_O [112]. These predictions provided an early theoretical foundation for the functionalization strategies discussed in Section 5.

Complementary insight also comes from adsorption-based sensing in other porous or defect-engineered materials, which parallels the vacancy- and heteroatom-defect strategies discussed for CNTs (Section 3.3) and the functionalization approaches in Section 5.

In zeolites, controlled thermal treatment under a reducing H_2_/N_2_ atmosphere generates oxygen vacancies that increase the density of Lewis and Brønsted basic sites and thereby enhance the material’s interaction with oxygenated volatile organic compounds (OVOCs); a Faujasite-type zeolite treated at 600 °C showed an approximately 280-fold enhancement in surface photovoltage response to acetone vapor, measured by a scanning Kelvin probe, relative to the untreated material, with about 67% signal recovery under UV illumination [113]. Similarly, amine-functionalized organic sorbents illustrate how chemical basicity can be engineered for selective CO_2_ capture: a phenothiazine-based small organic molecule bearing amine functionality was shown to chemisorb CO_2_ through carbamate-type interactions [114]. These defect- and amine-based adsorption strategies, established in non-CNT porous and molecular sorbents, provide a useful design reference for functionalizing CNT surfaces toward more selective, reversible OVOC and CO_2_ sensing.

Importantly, these theoretical results should be interpreted as a mechanistic guide for experimental sensor design rather than as isolated adsorption calculations. For pristine CNTs, DFT-predicted strong electron-withdrawing interactions of NO_2_ and O_2_ are consistent with the experimentally observed high sensitivity of p-type CNT networks to oxidizing gases, whereas weak physisorption of CO_2_, CH_4_, H_2_, and N_2_ explains why pristine CNTs usually require functionalization, catalytic metals, or hybrid interfaces for practical detection. Similarly, the predicted enhancement of molecular adsorption by heteroatom doping supports the experimental use of doped CNTs, oxygenated CNTs, conducting polymers, metal oxides, MOFs, and TMD/CNT heterostructures to create stronger or more selective adsorption sites. Recent hybrid examples, such as MWCNTs-InSe, further show how DFT-derived charge transfer, adsorption selectivity, and interfacial electric-field formation can be connected to experimentally observed NO_2_ selectivity and resistance modulation. Thus, DFT calculations provide a useful bridge between molecular-level adsorption energetics and macroscopic sensing parameters such as response magnitude, selectivity, recovery, and humidity tolerance [54,111,112,115].

### 3.3. Limitations of Pristine CNTs

Despite their attractive intrinsic response, pristine CNTs face two well-recognized limitations as gas sensors. First, their selectivity is poor: because the resistive response derives from a generic charge-transfer interaction, many analytes produce qualitatively similar signals, and only gases with sufficiently large adsorption energy—principally NO_2_ and NH_3_—are detected with high sensitivity [11,12,13,75,76,116,117]. Gases of major practical importance, including CO, CO_2_, CH_4_, and H_2_, interact only weakly with the bare nanotube wall and yield little or no signal. Second, the strong interaction that makes adsorption detectable also impedes desorption, so recovery is often slow and incomplete at room temperature [118,119]. Mitigation by mild heating with an integrated micro-heater or brief ultraviolet illumination has been demonstrated, but the most general solution is chemical or physical functionalization, which both broadens the range of detectable gases and enhances sensitivity and selectivity.

**Table 3:** Dominant sensing mechanisms in CNT-based gas sensors.

Charge transfer and resistance modulation	Adsorbed donor or acceptor molecules exchange charge with p-type CNTs, changing carrier density and resistance.	CNT sidewalls, defect sites, oxygen-containing groups, and adsorbed oxygen species.	Pristine CNT networks, doped CNTs, receptor-modified CNTs.	NH_3_, NO_2_, O_2_.	[14,102,103,111,112,113]	Provides room-temperature sensitivity but is intrinsically broad and often insufficiently selective.
Schottky-barrier modulation	Gas adsorption changes the work function or local electrostatic environment at a metal/CNT contact, modifying carrier injection across the Schottky barrier.	Metal-CNT electrode interface; contact-dominated resistance.	CNT FETs, Pd/SWCNT Schottky contacts, SWCNT-film diode sensors.	H_2_, NO_2_, NH_3_.	[59,61,104,105,106]	Small interfacial work-function changes can produce large conductance changes; electrode design is critical.
p-n or Schottky-type heterojunction modulation	Gas adsorption modulates depletion layers, band bending, or built-in fields formed at CNT/semiconductor interfaces.	Interfaces between p-type CNTs and n-type oxides or 2D semiconductors; CNT/TMD or CNT/oxide junctions.	WO_3_/CNT, Sn-TiO_2_/rGO/CNT, CNT/WS_2_, WSe_2_/MWCNT, MWCNTs-InSe.	NH_3_, NO_2_, H_2_, VOCs.	[38,40,50,51,52,120]	Improves sensitivity and selectivity through interface-level barrier modulation, but performance depends on junction uniformity.
Catalytic dissociation, spillover, and work-function change	Catalytic nanoparticles dissociate target gases; adsorbed species spill over to CNTs or alter nanoparticle work function, changing CNT resistance or contact barriers.	Noble-metal nanoparticles and metal/CNT interfaces.	Pd/CNT, Pt/CNT, PdAu-PMP, Pt/CNT/graphene hybrids.	H_2_, CO, VOCs.	[19,20,21,22,23,24,28,115,121,122,123,124,125,126]	Essential for weakly interacting gases such as H_2_; nanoparticle size, alloying, and stability determine reversibility.
Tube–tube junction, tunneling, and percolation modulation	Gas adsorption, polymer swelling, or dielectric changes modify tunneling barriers and inter-tube contact resistance in CNT networks.	CNT-CNT junctions, polymer/CNT interfaces, network density, and percolation pathways.	PANI/CNT, PPy/CNT, PEDOT/CNT, peptide-functionalized CNT networks.	NH_3_, NO_2_, VOCs.	[30,31,32,33,34,35,127,128,129]	Network density and junction quality control signal-to-noise ratio, reproducibility, and mechanical stability.
Adsorption-site and molecular-sieving effects	Selective receptors or porous frameworks preconcentrate target molecules or exclude interferents based on affinity, pore size, polarity, or functional groups.	MOF pores, polymer receptors, amine groups, peptide layers, oxygenated or defect-rich carbon sites.	ZIF-8/MWCNT, ZIF-8/CNT, PEI-CNT, peptide-CNT, amine-GO/CNT.	CH_4_, CO_2_, NH_3_, VOCs.	[129,130,131,132,133,134]	Improves selectivity and humidity tolerance, but receptor binding must remain reversible for repeated operation.
Surface reaction with chemisorbed oxygen species	Reducing or oxidizing gases react with adsorbed oxygen species on oxide or defect-rich surfaces, releasing or trapping electrons and changing resistance.	Metal-oxide grains, oxide/CNT interfaces, oxygen vacancies, and chemisorbed O_2_^−^/O^−^ species.	Fe_2_O_3_/MWCNT, TiO_2_/MWCNT, SnO_2_/CNT, ZnO/CNT, CuO/CNT.	NH_3_, H_2_, H_2_S, CO, VOCs.	[25,26,42,43,44,45,46,47,48,49,51,58,135,136,137,138,139,140,141]	Enhances response to reactive gases but can require elevated temperature or recovery assistance.
Carrier-lifetime and scattering modulation	Weakly interacting adsorbates or gas collisions alter carrier scattering and lifetime without substantial charge transfer.	CNT mats and networks exposed to weakly adsorbing or inert gases.	Pristine SWCNT mats, thermopower-based CNT studies.	N_2_, He, weakly adsorbing gases.	[110]	Shows that CNT responses may arise even without strong charge transfer; useful for interpreting baseline shifts.
Recovery and desorption-assisted transduction	External stimuli such as heat, UV/visible light, electrical pulses, self-heating, or airflow accelerate desorption and restore the baseline.	Strongly adsorbed species at defects, receptors, polymers, nanoparticles, and porous interfaces.	Self-heated CNTs, filament-heater CNT sensors, UV-assisted CNT networks, pulse-recovered memristor/CNT devices.	NO_2_, NH_3_, H_2_S, VOCs.	[142,143,144,145,146,147,148]	Recovery strategy should be reported with power consumption, duty cycle, and humidity dependence.

## 4. Device Architectures

CNTs have been integrated into several distinct sensor architectures, each exploiting a different transduction principle. The choice of architecture determines the measurand, the range of detectable gases, and the power budget of the device.

### 4.1. Chemiresistors, Transistors, Ionization, and Resonant Devices

**Chemiresistors.** CNT gas sensors are most commonly implemented as chemiresistors, in which a CNT film or network bridges two—often interdigitated—electrodes and the analyte is detected through a change in DC resistance or conductance. Interdigitated electrodes provide a large sensing area and good contact, and the response is read out directly [149]. Chemiresistors are attractive because they require simple fabrication and low-cost readout electronics, which accounts for their predominance and their suitability for distributed sensor nodes and flexible substrates. Their main weakness is limited intrinsic selectivity, because a single resistance signal compresses several physical contributions into one output (Figure 6) [15,16].

**Field-effect transistors (FETs).** When a semiconducting SWCNT bridges source and drain over a gated dielectric, gas-induced charge transfer modulates the channel conductance, and the gate voltage provides an additional control parameter. Gas adsorption can modulate the channel conductance, threshold voltage, mobility, and contact barriers, and gate-voltage sweeps can separate doping effects from mobility or Schottky-barrier effects. Single-tube and network FETs have shown conductance changes of several orders of magnitude for NO_2_ and large, reversible responses to alcohol vapors [150]. CNT FETs can deliver high sensitivity at low power, but device-to-device reproducibility, semiconducting-tube enrichment, and integration with scalable fabrication remain important challenges [151,152].

**Figure 7:**
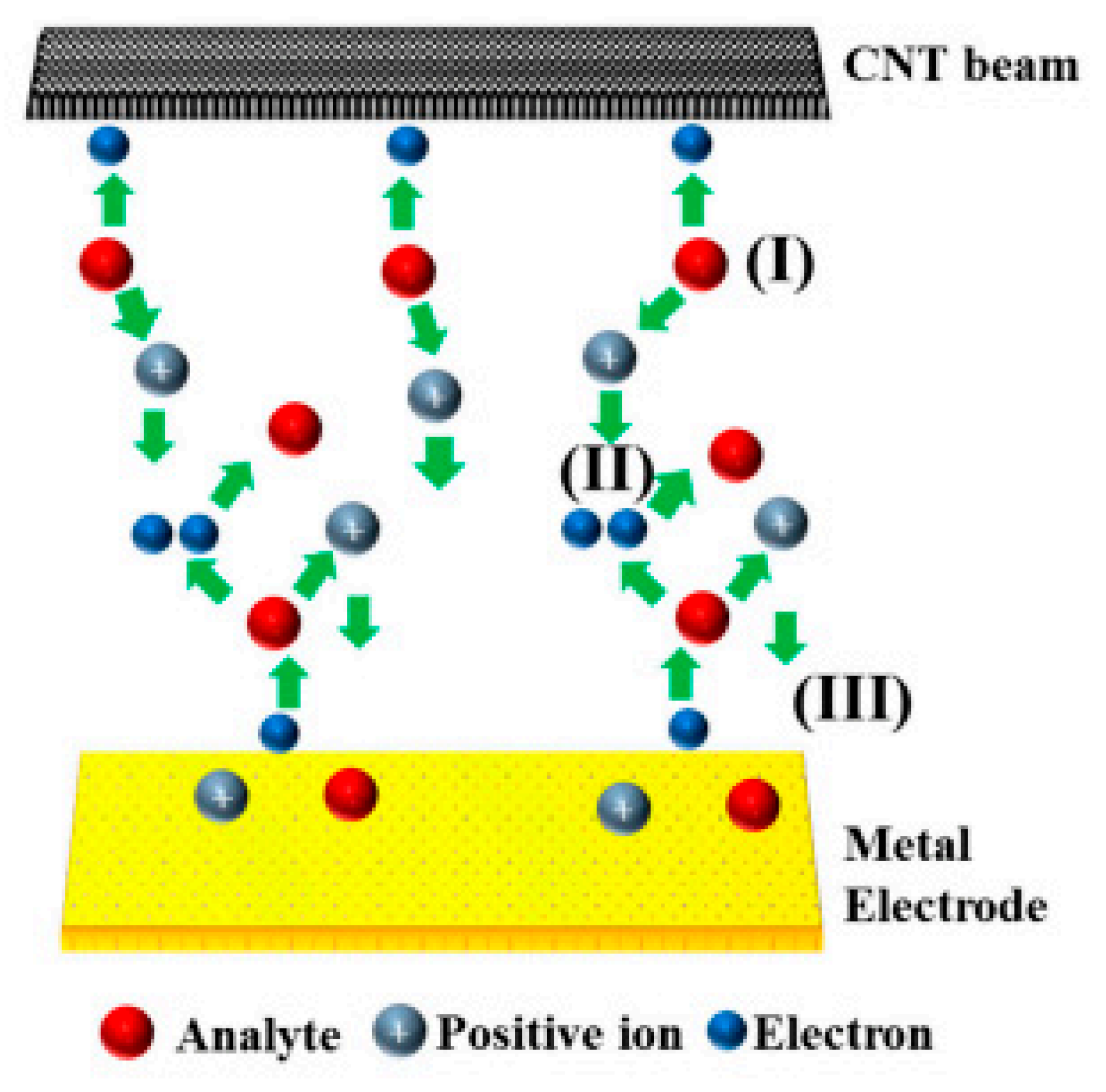
Sensing mechanism with a positive nanotip: (I) nanotip ionization, (II) impact ionization, and (III) electron recombination. All panels adapted with permission from ref. [18].

**Ionization sensors.** Rather than relying on adsorption, ionization sensors identify gases by their characteristic breakdown voltage. The sharp tips of vertically aligned CNTs generate intense local electric fields that initiate corona discharge at much lower voltages than flat metal electrodes; Modi and co-workers reduced the breakdown voltage of air from 960 V to 346 V using a MWCNT-film anode and distinguished NH_3_, CO_2_, N_2_, O_2_, He, Ar, and air by their fingerprint voltages [17]. Since no adsorption is required, ionization sensors can detect inert gases and species with low adsorption energy, and later designs employing CNT cathodes, short electrode gaps, or dielectric-barrier coatings lowered the operating voltage to a few volts (Figure 7) [18].

**Figure 8:**
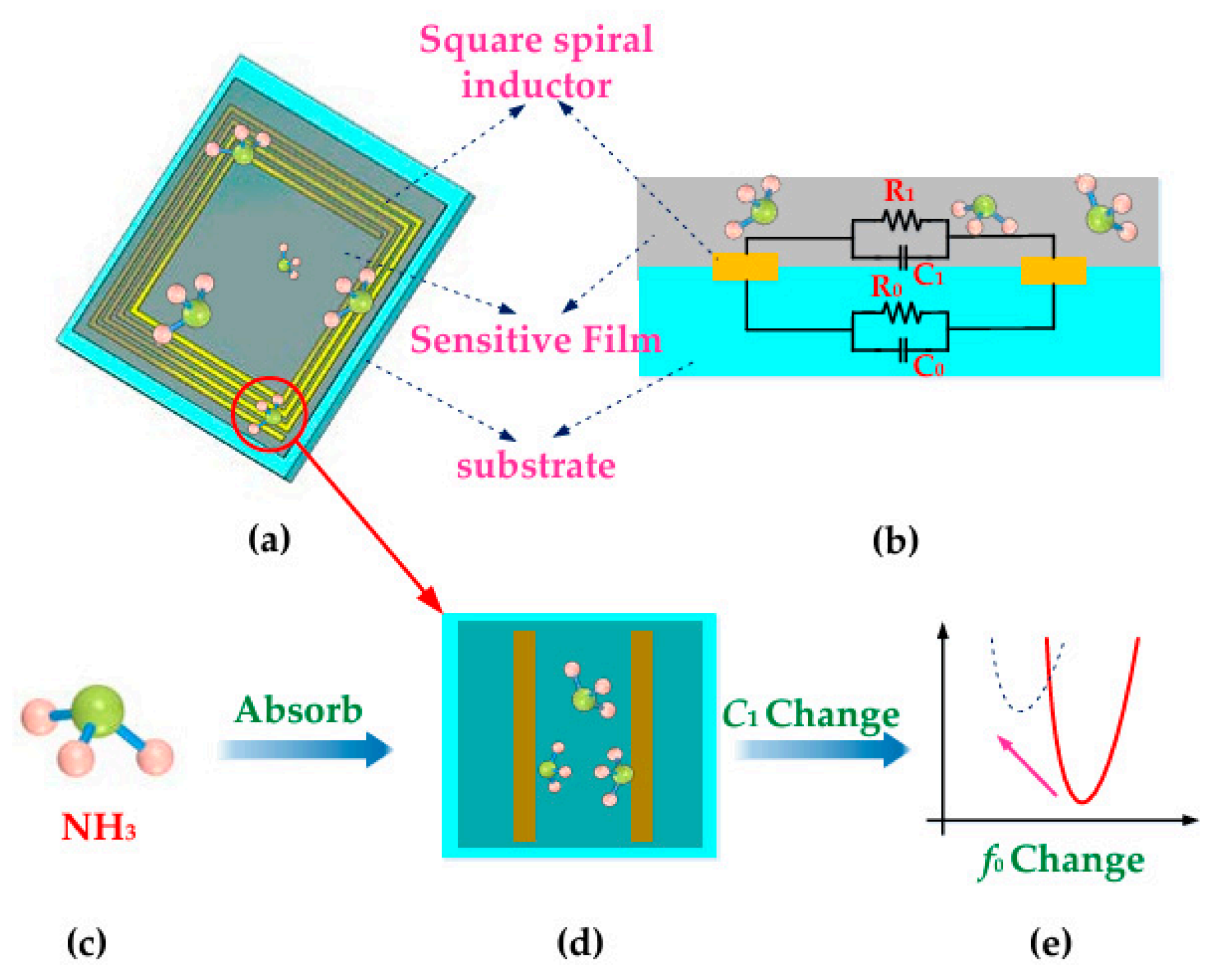
(**a**) Sensor model. (**b**) Sensor circuit model. (**c**) Ammonia molecule model. (**d**) Diagram of adsorbed gas molecules. (**e**) Trend of resonant frequency. All panels adapted with permission from ref. [142].

**Capacitive and resonant sensors.** CNT films also serve as the active element in capacitors and resonators. Parallel-plate and SWCNT capacitors respond to humidity and a broad range of vapors through changes in dielectric constant and field-enhanced polarization at the tube tips [153]. In resonant-circuit and wireless inductor–capacitor designs, gas adsorption shifts the resonant frequency via the permittivity change in the CNT layer, enabling passive, remotely interrogated sensing suitable for sealed environments (Figure 8) [142,143].

### 4.2. Performance Parameters

A rigorous comparison must distinguish response from sensitivity. Response is commonly expressed as a relative change such as ΔR/R_0_, ΔG/G_0_, or R_g_/R_a_, depending on whether the device increases or decreases resistance upon exposure. Sensitivity is the slope of the calibration curve in a defined concentration range; a high response at one concentration does not necessarily imply a low detection limit or high quantitative accuracy. The limit of detection (LOD) is typically estimated from the signal-to-noise ratio or from 3σ divided by the calibration slope. Response and recovery times are kinetic metrics, often reported as the time to reach 90% of the final response during exposure and to return 90% toward baseline during a purge.

Recovery is a major weakness for many CNT sensors because analytes such as NH_3_, NO_2_, and polar VOCs bind strongly to defects, oxygen-containing groups, or polymer receptors. It can be accelerated by mild heating, UV or LED illumination, carrier-gas flow, humidity control, or catalytic functionalization, each of which affects power consumption and device complexity [121,122,144].

Selectivity and specificity are the key practical barriers. Real environments contain humidity, oxygen, CO_2_, VOC mixtures, and temperature fluctuations, all of which alter baseline resistance and adsorption equilibria. For breath analysis, humidity can exceed 90% relative humidity and strongly interfere with NH_3_, NO, acetone, and VOC detection; for environmental monitoring, NO_2_, O_3_, SO_2_, NH_3_, CO, and VOCs can coexist, requiring sensor arrays and pattern recognition rather than a single receptor. Table 4 lists the metrics recommended for a meaningful comparison across studies.

## 5. Functionalization Strategies

Pristine CNT networks provide a high surface area, efficient charge transport, and room-temperature operation, but they also suffer from several intrinsic limitations, including nonspecific charge-transfer responses, weak adsorption of chemically inert gases, slow recovery after strong adsorption, humidity-induced baseline drift, and device-to-device variability caused by differences in chirality, defect density, residual catalyst content, and network morphology. Therefore, CNT functionalization is required not only to increase sensitivity but also to define the dominant sensing mechanism. Noble-metal nanoparticles improve catalytic dissociation and spillover, metal oxides and TMDs introduce heterojunction and surface-redox pathways, conducting polymers enable molecular doping/dedoping and swelling-mediated percolation changes, and MOFs, graphene derivatives, peptides, and molecular receptors provide adsorption-site engineering, molecular sieving, humidity screening, or analyte-specific recognition. These limitation–strategy relationships are considered throughout Section 5 and are further reflected in the mechanism and gas-specific comparison tables.

**Figure 9:**
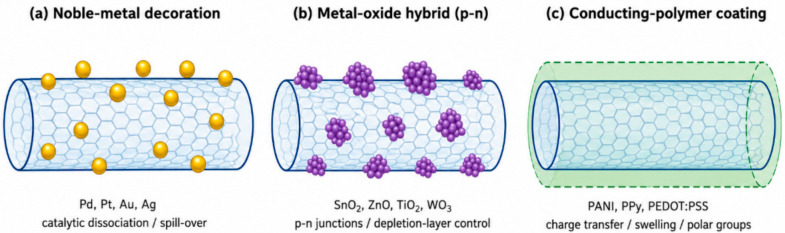
Functionalization strategies for CNT gas sensors. (**a**) Noble-metal nanoparticle decoration provides catalytic dissociation and spillover; (**b**) metal-oxide hybrids form random p–n junctions and depletion layers; (**c**) conducting-polymer coatings add charge-transfer and swelling responses through polar functional groups. Schematic illustration prepared with the assistance of ChatGPT (GPT-5.5)-based AI tools and verified by the author.

Functionalization is the principal route to overcoming the selectivity and recovery limitations of pristine CNTs. Decorating or hybridizing the nanotube wall with noble metals, metal oxides, or conducting polymers introduces specific binding sites, catalytic pathways, or junction effects that extend the range of detectable gases and amplify the response (Figure 9).

### 5.1. Noble-Metal Decoration

**Figure 10:**
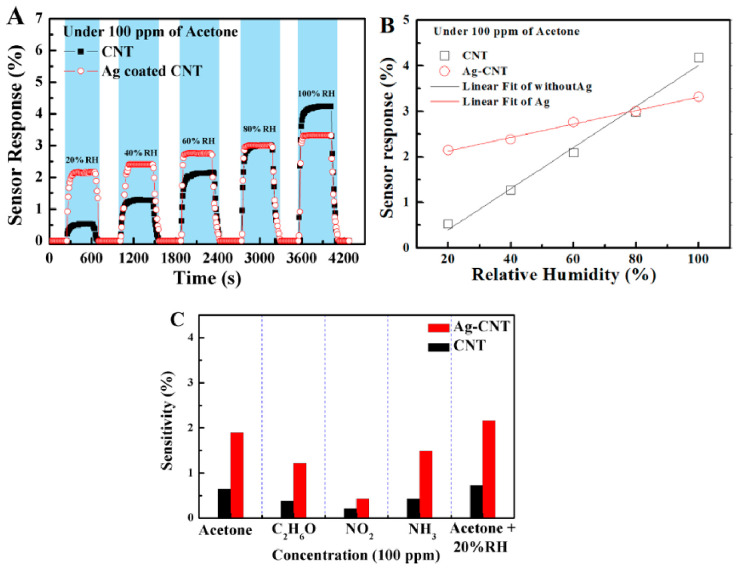
Effect of varying humidity for pure CNT and Ag-CNT sensors under 100 ppm of acetone. (**A**) Sensor responses of CNTs, with and without Ag NPs, are measured at different values of relative humidity (RH). (**B**) Sensor response of CNT-based sensors, with and without Ag NPs, as a function of RH. (**C**) Selectivity of CNT sensors, with and without Ag NPs, to various target gases (acetone, ethanol, NO_2_, ammonia, and acetone with water). All panels adapted with permission from ref. [125].

Decoration with noble metals such as Au, Pt, Pd, Rh, Ag, Ni, and Cu enhances both the sensitivity and the selectivity of CNTs without altering their p-type character (Figure 10) [115,123,124,125]. The metal nanoparticles act as catalytic sites at which target molecules dissociate or adsorb, transferring charge to or from the underlying nanotube and accelerating the surface reaction, thereby shortening response times and lowering the working temperature. Hydrogen sensing is the classic example: pristine CNTs are essentially blind to H_2_, but Pd or Pt nanoparticles dissociate molecular hydrogen into atomic species that lower the metal work function and inject charge into the tube, rendering Pd/Pt–CNT devices highly responsive and self-recovering at room temperature [19,20,21]. This sequence—dissociation of the analyte at the metal particle followed by spillover and charge transfer to the CNT—is the general principle behind noble-metal sensitization, and it has likewise enabled detection of CO, benzene, NH_3_, and other gases; arrays functionalized with several different metals can discriminate among analytes and even detect two gases simultaneously [22,23,24,25,26]. Particle size is critical: very small particles provide more active sites but can sinter or oxidize, whereas large particles are more stable but offer less interfacial area, and metallic nanoparticles may oxidize during cycling, degrading the response over time [19,20,21,22,25,26]. Deposition can be achieved by wet or photochemical reduction, sputtering, evaporation, electrodeposition, or direct mixing; in all cases, the response should be interpreted with CNT-only, metal-only, and physical-mixture controls to distinguish true interfacial synergy from simple additive effects.

### 5.2. Metal-Oxide Hybrids

**Figure 11:**
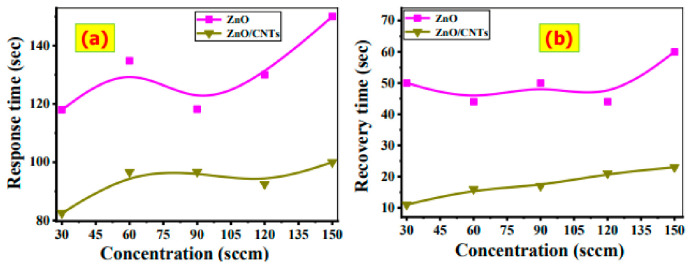
(**a**) Response and (**b**) recovery times for ZnO and ZnO/CNTs films under the flow-rate conditions reported in the original source. The unit sccm denotes gas flow rate, not concentration. All panels adapted with permission from ref. [43].

Hybridizing CNTs with semiconducting metal oxides such as SnO_2_, NiO, ZnO, Co_3_O_4_, and WO_3_ combines the high-temperature gas-sensing capability of the oxide with the room-temperature sensitivity of the nanotube [135,136,137,138,139]. Since most of these oxides are n-type and CNTs are p-type, the interface forms p–n junctions and associated depletion layers, whose modulation by oxidizing or reducing gases produces large conductance changes. In contrast to a planar bilayer, the junctions form randomly at each CNT–oxide contact, which multiplies adsorption sites but renders the response strongly dependent on the relative concentration, particle size, and dispersion of the two components [140]. Comparative data indicate that oxide modification frequently yields the highest sensitivities of the three strategies and can shift the optimum operating temperature toward room-temperature; SnO_2_–CNT composites, for instance, detect NO_2_, NH_3_, and H_2_S at room temperature with a high response [42]. SnO_2_/CNT, ZnO/CNT (Figure 11), WO_3_/CNT, TiO_2_/CNT, CuO/CNT, and NiO/CNT systems have been reported for NO_2_, NH_3_, CO_2_, H_2_S, H_2_, ethanol, acetone, formaldehyde, methane, and other analytes, with synthesis routes including ultrasonic mixing, in situ catalytic pyrolysis, precipitation, and ultrasonic spray pyrolysis, each producing distinct morphologies [43,44,45,46,47,48,49].

Representative metal-oxide/CNT case studies emphasize how catalytic sites and percolation pathways act together: Ag-loaded Cu_2_O/CNTs improved room-temperature NH_3_ sensing by promoting electron transfer and oxygen activation, giving a maximum response of 76.3 to 100 ppm NH_3_ and shortening the response time from 320 s (Cu_2_O/CNTs) to 193 s after optimized Ag loading [127], while CNT-templated In_2_O_3_ nanowire-like networks reached a response of 63.5 to 100 ppm formaldehyde at 300 °C—about 30 times that of compact In_2_O_3_ nanoparticle films—by preserving an open one-dimensional morphology [128].

### 5.3. Conducting-Polymer Composites

Conducting polymers, including polyaniline (PANI), polypyrrole (PPy), and poly(3,4-ethylenedioxythiophene) (PEDOT), have been widely investigated as active components for room-temperature gas sensors because of their tunable electrical conductivity, facile synthesis, structural flexibility, low cost, and reversible doping/dedoping chemistry. In particular, their conjugated backbones and redox-active functional groups allow gas adsorption to induce measurable resistance changes through charge transfer, protonation/deprotonation, and polymer-chain swelling. However, pristine conducting polymers often suffer from limited environmental stability, slow recovery, and insufficient charge-transport pathways. Hybridization with carbon nanotubes has therefore become an effective strategy to improve sensitivity, response/recovery kinetics, and mechanical robustness: in these composites, CNTs provide a highly conductive, high-surface-area scaffold, while the conducting polymer supplies gas-specific interaction sites.

PANI/CNT composites have been studied most extensively for NH_3_ detection. Since NH_3_ is an electron-donating, basic molecule, it interacts strongly with protonated PANI and converts the conductive emeraldine-salt form into the less conductive emeraldine-base form, thereby increasing the resistance of p-type PANI-based sensors. When PANI is coated on CNTs, the CNT network facilitates charge transport and provides additional adsorption pathways, giving an enhanced sensing performance compared with either component alone. Abdulla et al. prepared a PANI/MWCNT nanocomposite by in situ oxidative polymerization of aniline on carboxylated MWCNTs [30]. A uniform PANI layer of approximately 7 nm formed on the MWCNT surface, producing abundant active sites for NH_3_ adsorption and efficient interfacial charge transfer. The resulting sensor showed a response of 15.5% to 2 ppm NH_3_ and 32% to 10 ppm NH_3_ at room temperature, with a rapid response/recovery time of 6/35 s at 2 ppm—much better than carboxylated MWCNTs alone, demonstrating the importance of a thin and uniform polymer coating.

**Figure 12:**
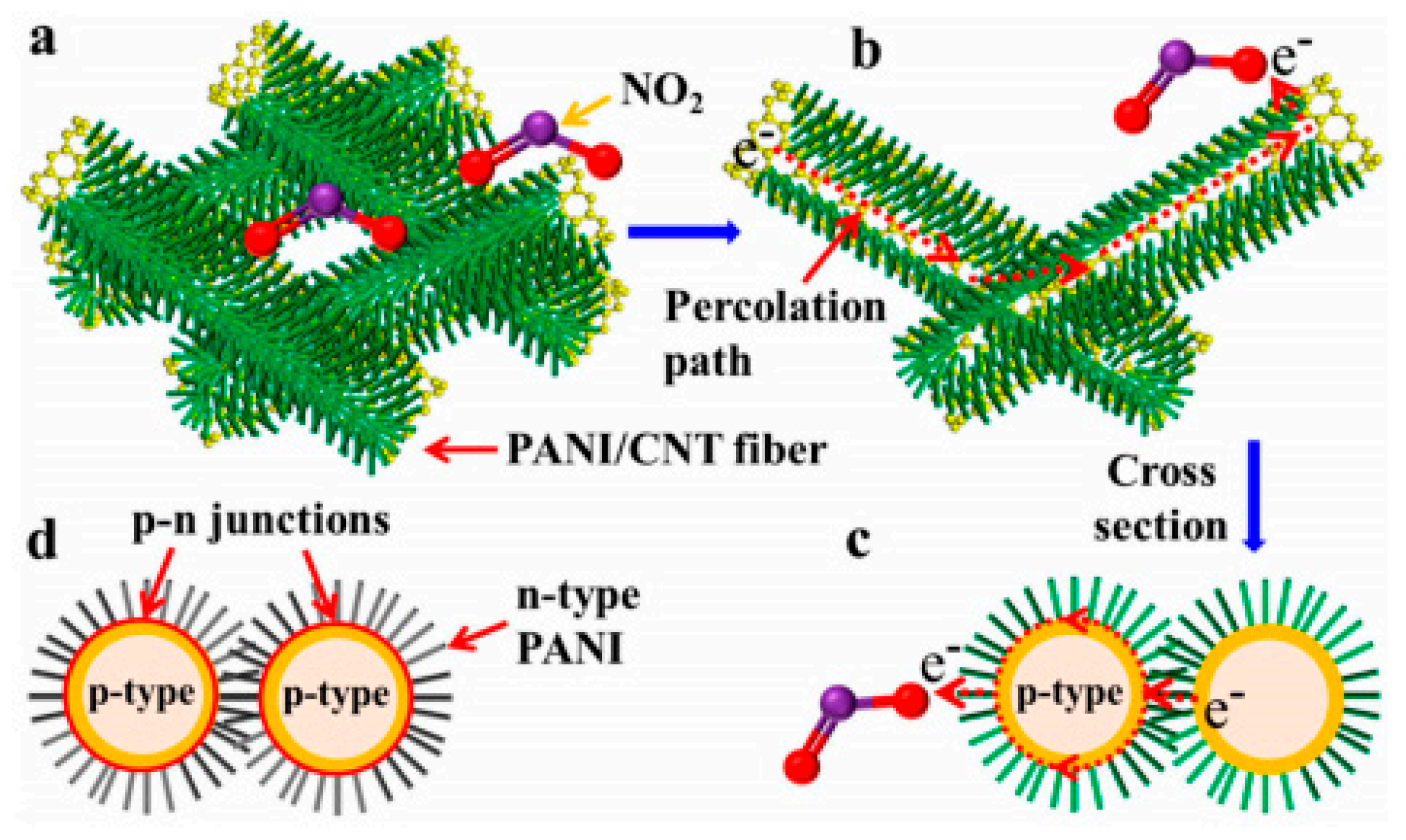
Possible sensing mechanism: (**a**) sketch diagram of conductive network of hierarchical pPANI/CNT fibers, (**b**) percolation path through conjugate interfaces of PANI and MWCNTs, (**c**) cross-section of PANI/CNT fibers, and (**d**) p–n heterojunction structure of hierarchical n-PANI/CNT fibers. All Panels adapted with permission from ref. [31].

Other PANI/CNT architectures show that the interfacial structure strongly governs sensing behavior. Zhang et al. fabricated one-dimensional hierarchical PANI/CNT fibers and demonstrated that interface engineering can tune the sensing polarity and selectivity [31]. In this system (Figure 12), p-type PANI/CNT exhibited high sensitivity toward oxidizing NO_2_, whereas heat-treated n-type PANI/CNT showed improved NH_3_ sensing through the formation of local p–n heterojunctions between n-type PANI and p-type CNTs. The response times to 50 ppm NO_2_ and NH_3_ were only 5.2 and 1.8 s, respectively, and the estimated detection limits were 16.7 ppb for NO_2_ and 6.4 ppb for NH_3_. These results indicate that the CNT/polymer interface can be used not only to enhance conductivity but also to modulate the depletion layer and carrier transport. Hierarchical PANI/CNT fibers demonstrate how conducting-polymer/CNT interfaces can tune both transport and selectivity: the CNT network provides conductive pathways, whereas the PANI shell controls acid/base interactions, p–n transition, and selective responses toward NO_2_ and NH_3_ [31].

Xue et al. prepared a flexible PANI/CNT nanocomposite film on PET substrates by in situ polymerization with simultaneous film deposition [32]. The interconnected network consisted of PANI-nanoparticle-coated CNTs and PANI nanofibers, enabling efficient charge transport and rapid gas diffusion. The device detected NH_3_ from 200 ppb to 50 ppm at room temperature, with response/recovery times of 85/20 s, excellent selectivity over volatile organic compounds, and a stable response even after repeated bending—highlighting the suitability of PANI/CNT composites for lightweight, portable, low-power sensor platforms.

The recovery issue of polymer/CNT NH_3_ sensors has also been addressed. Sharma et al. compared MWCNT–PEDOT and MWCNT–PANI composites for room-temperature NH_3_ detection [33]. Both showed high sensitivity but poor room-temperature recovery because NH_3_ molecules adsorb strongly on CNT-related active sites. MWCNT–PEDOT exhibited higher sensitivity and better thermal stability than MWCNT–PANI, and by combining moderate thermal treatment with a small DC electric field, the recovery time of the MWCNT–PEDOT sensor was reduced from 48 h to 20 min—suggesting that external stimulation can help desorb strongly bound NH_3_ and improve cycling stability.

PPy/CNT composites provide another example of conducting-polymer-enhanced CNT sensing. An et al. fabricated an SWCNT/PPy nanocomposite sensor through in situ chemical polymerization of pyrrole on SWCNTs [34]. The PPy coating increased the specific surface area and supplied additional active adsorption sites, while the SWCNT network reduced the baseline resistance and improved charge transport. The SWCNT/PPy sensor exhibited roughly ten times higher sensitivity toward NO_2_ than pure PPy; however, incomplete recovery was observed at room temperature, implying that both physisorption and chemisorption contributed to the response. This example shows that conducting-polymer coatings can substantially amplify CNT sensitivity, but the strength of the gas–polymer interaction must be carefully controlled to maintain reversibility.

PEDOT-based CNT composites have recently been extended to dual-gas detection. Du et al. constructed Pd&PEDOT@CNT nanoarchitectures by ultrasound-assisted redox self-assembly [35]. The optimized structure contained a uniform PEDOT layer and well-dispersed Pd nanoparticles with sub-nanometer interface gaps on CNTs. This hybrid sensor showed opposite response directions toward two gases at room temperature: a resistance-decreasing response to H_2_ over 0.2–40 vol% and a resistance-increasing response to NH_3_ from 0.5 ppm to 30 vol%. The H_2_ response originated mainly from PdH_x_ formation and the reduction in interparticle gaps between Pd nanoparticles, whereas the NH_3_ response was attributed to PEDOT dedoping. Since the two target gases produced opposite signal polarity, H_2_ and NH_3_ could be discriminated using a single sensing material.

Flexible conducting-polymer films also provide useful design principles for CNT/polymer systems. Bai et al. fabricated transparent conducting films of hierarchically nanostructured PANI networks on PET substrates using sacrificial Ag-nanowire templates [36]. The quasi-one-dimensional PANI network showed improved NH_3_ sensitivity compared with particle-like PANI films while retaining optical transparency and flexibility, indicating that polymer morphology and network connectivity are critical parameters that can be translated to CNT/polymer hybrids.

**Figure 13:**
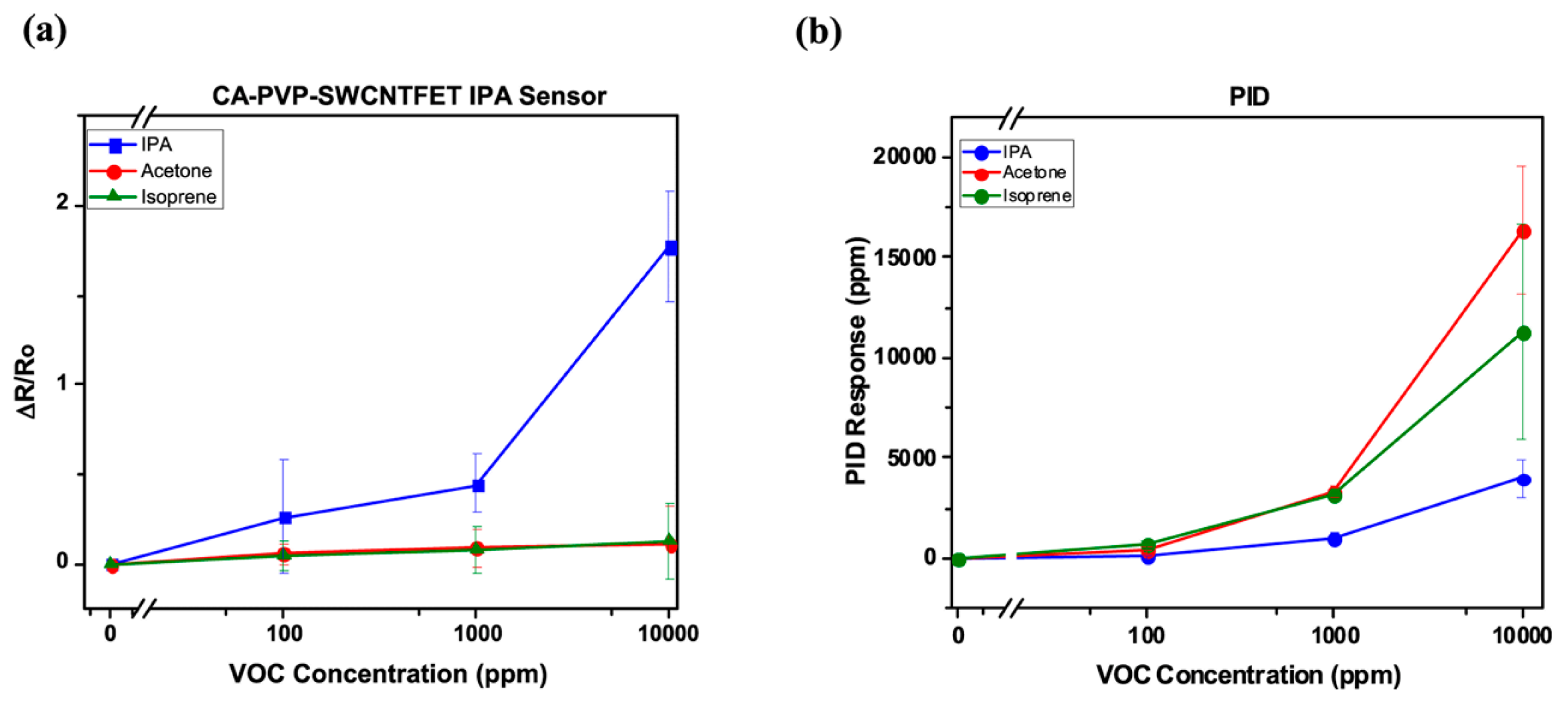
(**a**) CA−PVP−SWCNTFET and (**b**) commercial PID sensor responses at various chemical concentration point injections. CA−PVP−SWCNTFET shows higher responses to IPA as compared to the equivalent concentrations of acetone and isoprene gases. Commercial PID displays no specificity to IPA. All panels adapted with permission from ref. [37].

More recently, an electronic gas sensor was fabricated by coating SWCNTs with a semi-selective polymer matrix to detect isopropyl alcohol (IPA) vapor [37]. The synergistic combination of the analyte-capturing polymer and the highly conductive SWCNT network significantly enhanced the device’s electrical response, demonstrating a low limit of detection (LOD) of 19.3 ppm for IPA. Furthermore, this hybrid sensor exhibited a linear sensitivity profile across a concentration range of 20 to 100 ppm, proving its capability for reliable, room-temperature volatile organic compound (VOC) monitoring (Figure 13).

### 5.4. CNT/Graphene Composites

CNT/graphene composites have recently emerged as an effective hybrid platform for chemiresistive gas sensors because they combine the complementary advantages of one-dimensional CNTs and two-dimensional graphene-based materials. CNTs provide continuous, highly conductive charge-transport pathways, prevent restacking of graphene sheets, and create open diffusion channels for gas molecules. Graphene, graphene oxide (GO), and reduced graphene oxide (rGO), in turn, offer large accessible surface areas, abundant defect sites, and tunable oxygen-containing functional groups for gas adsorption. CNT/graphene composites can therefore overcome some limitations of pristine CNTs or graphene—such as weak gas-specific interactions, aggregation, and poor selectivity—while preserving room-temperature operation and mechanical flexibility.

The sensing enhancement in CNT/graphene composites is mainly attributed to three synergistic effects [154]. First, the interconnected CNT network improves electrical percolation and accelerates charge transfer across the sensing layer. Second, graphene derivatives introduce defect sites, oxygen functional groups, and local adsorption centers that increase analyte interaction. Third, the CNT–graphene interface can act as an additional charge-modulation region, where gas adsorption changes the local Fermi level and interfacial resistance. These features are particularly useful for low-concentration gas detection, because even small adsorption-induced charge-transfer events can be effectively converted into measurable resistance changes.

A representative example is the three-dimensional graphene–CNT network reported for NO_2_ detection [120]. This sensor showed a response of 9.1% to an extremely low NO_2_ concentration of 0.01 ppm at room temperature, with fast response/recovery times of 6/10 s. The excellent performance was attributed to the open three-dimensional architecture, which provided rapid gas-diffusion pathways and efficient charge transport, highlighting the importance of constructing porous CNT/graphene networks rather than simply mixing the two carbon materials.

**Figure 14:**
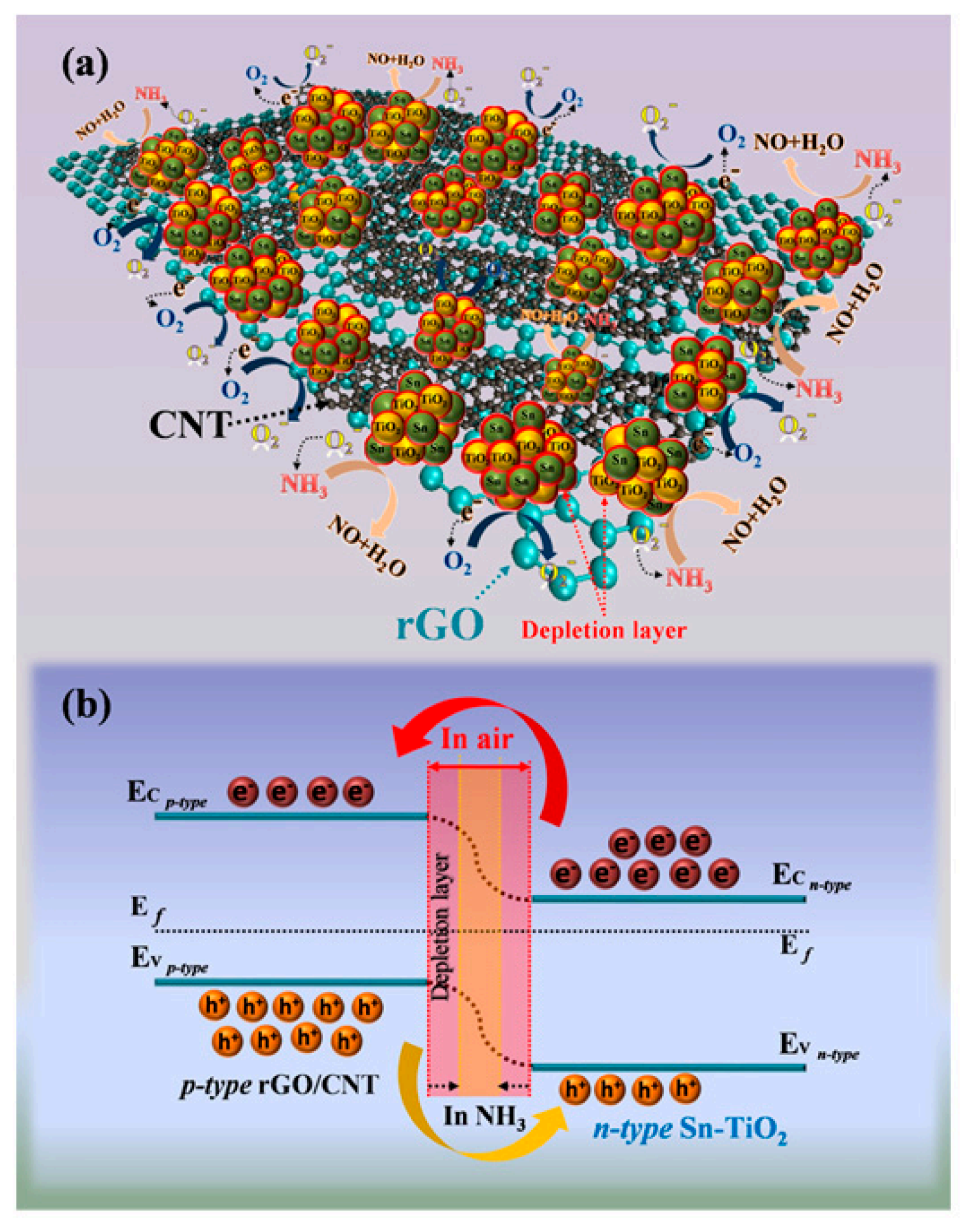
(**a**) Schematic of the proposed NH_3_-sensing mechanism and (**b**) p−n heterojunction of the Sn−TiO_2_@rGO/CNT nanocomposite gas sensor. All panels adapted with permission from ref. [156].

Metal-oxide-decorated CNT/graphene composites further improve sensitivity and selectivity by introducing catalytic sites and heterojunction interfaces. For example, rGO–CNT–SnO_2_ composites showed a high NO_2_ response of about 243% at 5 ppm, with response/recovery times of 13/87 s at room temperature [155]. Here, SnO_2_ provides chemically active adsorption sites for oxidizing gas molecules, while rGO and CNTs facilitate charge transport. Similarly, Sn–TiO_2_/rGO/CNT nanocomposites used for NH_3_ detection exhibited a response of 85.9% at 250 ppm NH_3_, with response/recovery times of 120/80 s at room temperature [156]. These results suggest that combining CNT/graphene frameworks with metal oxides is an effective route to amplify gas-induced resistance modulation. As shown in Figure 14, Sn–TiO_2_@rGO/CNT nanocomposites show that ternary oxide/carbon interfaces can enhance NH_3_ selectivity by combining Sn–TiO_2_ surface reactivity, rGO/CNT charge transport, and p–n heterojunction-mediated barrier modulation at room temperature [156].

**Figure 15:**
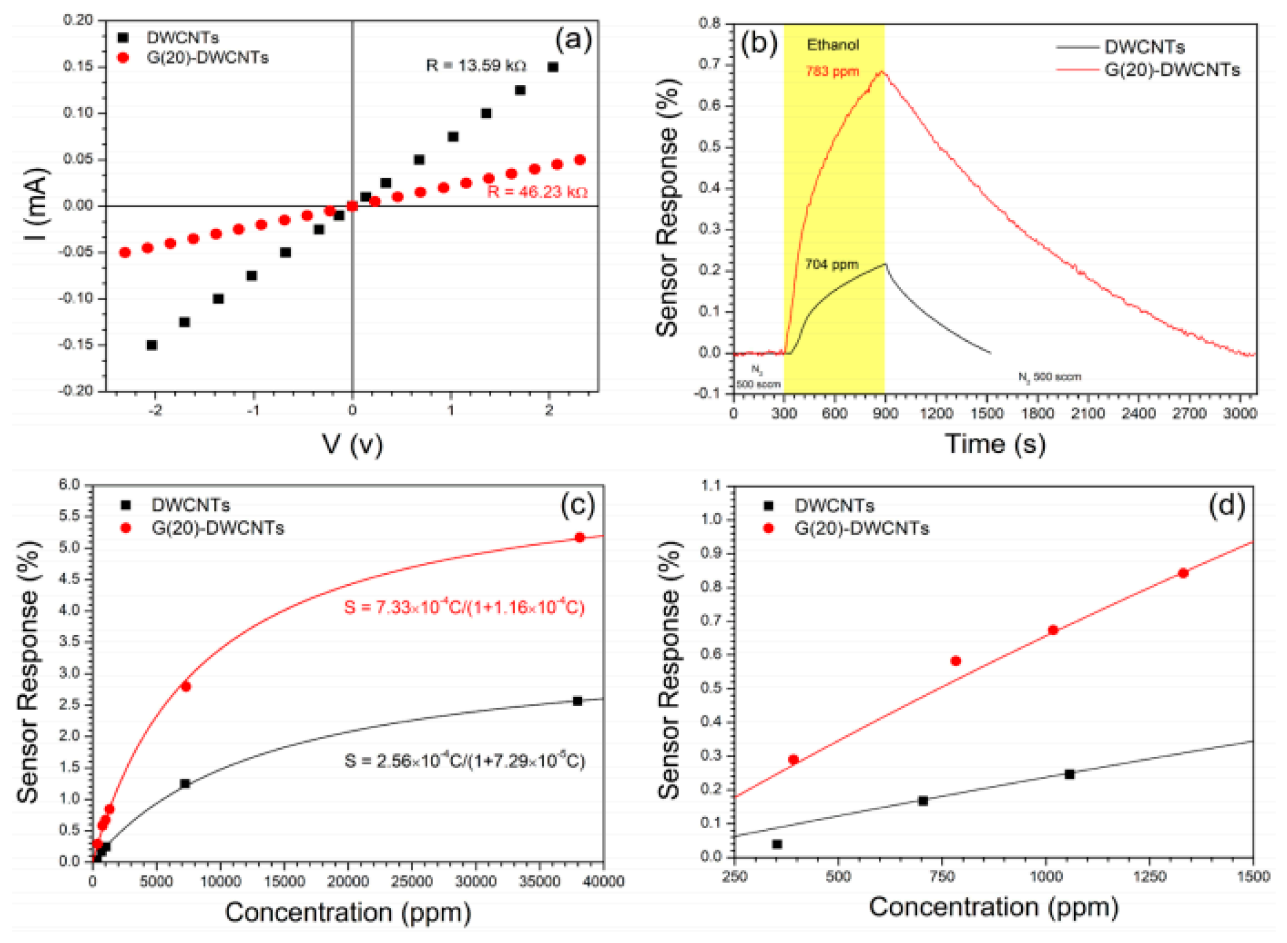
(**a**) I-V characteristics of DWCNTs and G(20)-DWCNTs. (**b**) Sensor response as a function of time for DWCNTs and G(20)-DWCNTs toward ethanol vapor. Sensor response of DWCNTs and G(20)-DWCNTs as a function of ethanol concentration at (**c**) high and (**d**) low concentrations. All panels adapted with permission from ref. [157].

Noble-metal-functionalized CNT/graphene hybrids are also promising, especially for hydrogen sensing. Monodispersed Pt nanoparticles on graphene-like carbon-wrapped CNTs showed a 42.8% response to 4% H_2_ at room temperature, with response/recovery times of 30/90 s and a detection limit of 0.04% [130]. A hybrid carbon nanostructure was synthesized by grafting petal-like graphene nanosheets (GNSs) onto double-walled carbon nanotubes (DWCNTs) using a two-step chemical vapor deposition method. Thanks to the significantly increased surface area for gas adsorption, the resulting G-DWCNT sensor exhibited an approximately 3-fold improvement in electrical response to ethanol vapor compared to pristine DWCNTs (Figure 15). This enhanced sensing capability is driven by an efficient charge transfer mechanism between the target gas molecules and the hybrid nanostructure network [157]. These examples demonstrate that catalytic nanoparticles provide gas-specific reactivity, whereas the CNT/graphene backbone enables rapid signal transduction.

CNT/graphene composites have also been applied to VOC and ammonia sensing. A CNT aerogel functionalized with oxygenated GO showed highly selective chlorobenzene detection; the optimized CNT aerogel–GO composite with 16% oxygen functionality exhibited a response of approximately 95% to 5 ppm chlorobenzene, with a detection limit of about 90 ppb [158]. The optimized oxygen functionality was critical because it supplied adsorption sites without excessively disturbing the conductive CNT network. For NH_3_ sensing, a CNT/rGO amine-functionalized composite showed a 70% response at 25 ppm NH_3_ and an ultralow detection limit below 0.2 ppb [159]. The high response is associated with the interaction between electron-donating NH_3_ and amine/oxygen-functionalized graphene sites, combined with efficient charge transport through the CNT network.

Ternary CNT/graphene/metal-oxide composites can further enhance performance by combining multiple functions in one architecture. For ethanol detection, MWCNT-decorated rGO–Co_3_O_4_ composites achieved a very high response of approximately 12,610% at 100 ppm ethanol at 180 °C, with response/recovery times of 50/20 s [131]. In this case, rGO and MWCNTs improved electron transport and provided additional adsorption sites, while mesoporous Co_3_O_4_ enhanced catalytic interaction with ethanol. CeO_2_-nanohexagon-decorated rGO/CNT composites also showed LPG-sensing performance, with a response of 42% to 400 ppm LPG at room temperature [129]. These examples indicate that CNT/graphene frameworks can serve as versatile conductive scaffolds for various catalytic or semiconducting modifiers.

In addition to common toxic and combustible gases, functionalized GO/CNT composites have been explored for explosive-vapor detection. An amine-functionalized GO/CNT nanocomposite was reported for trinitrotoluene detection with a low detection limit of 0.01 ppm [132]. Although this example is closer to vapor sensing than conventional gas monitoring, it demonstrates that molecular functionalization of the GO/CNT interface can introduce target-specific interactions and broaden the application range of CNT/graphene composite sensors.

To avoid an exhaustive catalogue, representative studies in Table 5 were selected to cover different CNT structures, functionalization strategies, target gases, operating temperatures, and device formats. Response values are compared as reported in the original studies because response definitions differ among reports.

### 5.5. Recent Advances in CNT-Based Functional Hybrid Interfaces

Recent studies have expanded CNT gas-sensor design from simple surface decoration toward more precisely engineered hybrid interfaces. These approaches use CNTs not only as conductive transducers but also as structural scaffolds, percolative networks, adsorption-site modulators, and interfacial charge-transport components. Representative examples include biomolecular CNT interfaces, MOF/MWCNT composites, CNT/TMD core–shell structures, humidity-resistant TMD/MWCNT heterostructures, and oxide/carbon hybrid films.

**Figure 16:**
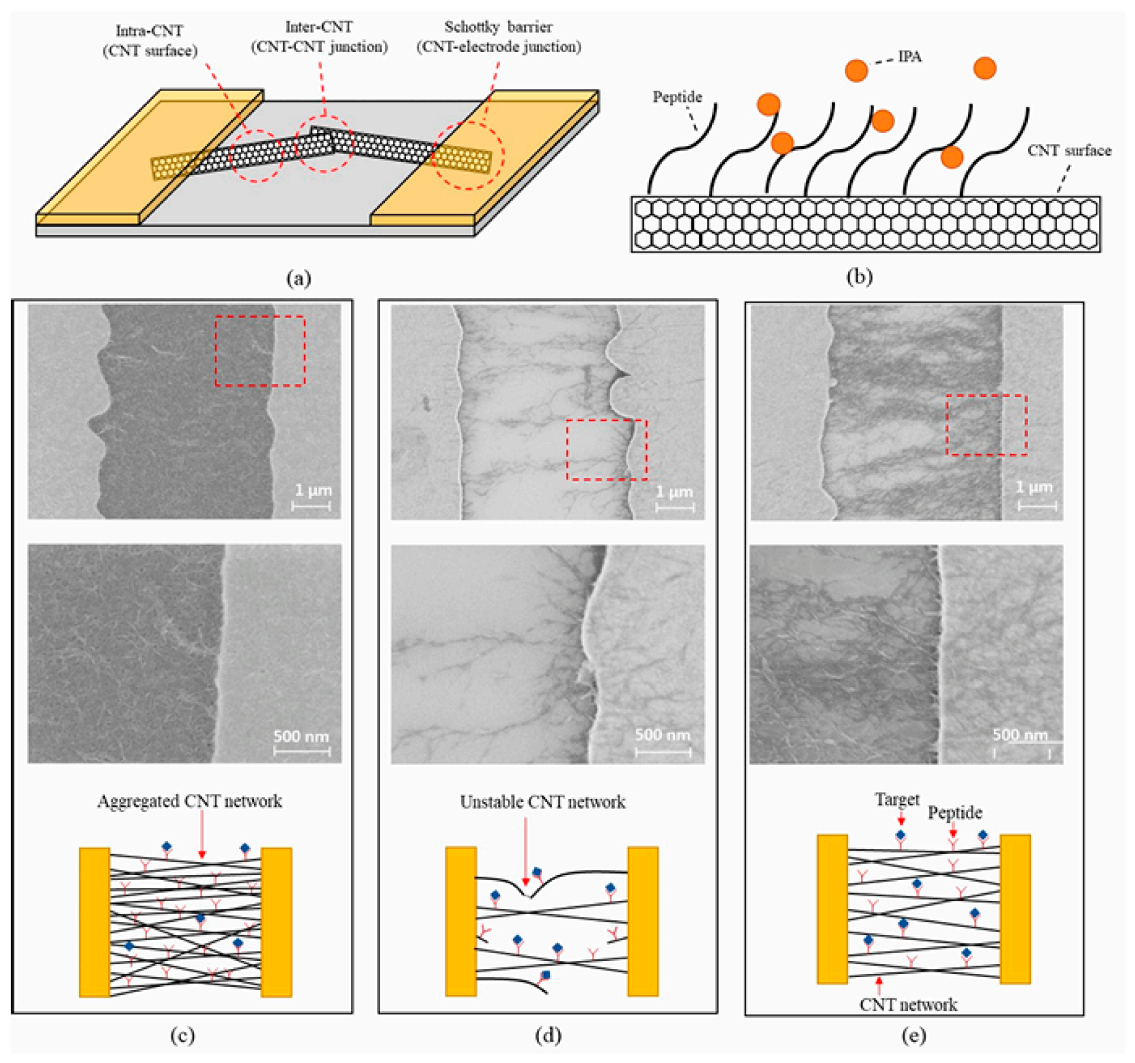
(**a**) Three dominant factors related to CNT sensors; (**b**) the schematics showing events between IPA and peptides on the CNT surface; (**c**) the aggregated CNT network, (**d**) the lack of the CNT network, and (**e**) the appropriate density of the CNT network. All panels adapted with permission from ref. [38].

Peptide-functionalized CNT chemiresistors provide an important example of how CNT network morphology affects sensor performance. Sim et al. investigated peptide-functionalized CNT chemiresistors with different nanotube densities and showed that sensor performance should be evaluated not only by response magnitude but also by noise level, signal-to-noise ratio, and device-to-device variation [38]. The importance of CNT network morphology is clearly illustrated in peptide-functionalized CNT chemiresistors. As summarized schematically in Figure 16, excessively dense CNT networks can reduce the effective molecular-event area through aggregation, whereas overly sparse CNT networks produce unstable percolation pathways, higher noise, and larger device-to-device variation. An intermediate nanotube density provides a more favorable balance between accessible receptor sites and stable electrical connectivity, resulting in an improved signal-to-noise ratio for VOC detection [38].

MOF/MWCNT composites represent another recent direction in adsorption-site engineering. Homayoonnia et al. reported a ZIF-8/MWCNT nanocomposite in which ZIF-8 provided selective adsorption sites for CH_4_, while MWCNTs supplied conductive pathways and reduced the electronic transport limitation of the insulating MOF framework [50]. The resulting composite enabled ppb-level methane detection with a reported LOD of approximately 0.22 ppb. This example shows that MOF/CNT hybridization can be used to combine molecular sieving or adsorption-site selectivity with CNT-mediated electrical readout.

CNT/TMD interfaces have also attracted attention because layered TMDs offer a tunable surface chemistry, defect sites, and heterojunction formation. Zribi et al. prepared CNT/WS_2_ core–shell nanostructures by atomic layer deposition and showed that the WS_2_ shell coverage strongly influences NO_2_ sensing behavior [51].

Intermediate WS_2_ coverage generated effective CNT/WS_2_ junctions and catalytic sites, leading to sensitive NO_2_ detection. Similarly, WSe_2_/MWCNT composites with abundant edge sites showed an enhanced NO_2_ response, high selectivity, and humidity-independent operation, which were attributed to p–p heterojunction formation, improved charge transport, hydrophobic MWCNT contributions, and abundant edge/defect sites in the WSe_2_/MWCNT composite [52].

Oxide/carbon hybrid films provide a further route for tuning the operating temperature and gas response. In TiO_2_-based hydrogen sensors, the addition of MWCNTs, graphene nanoflakes, or MXene modified the electrical and surface properties of TiO_2_ thick films and altered their H_2_ sensing behavior [53]. In particular, MWCNT and MXene incorporation reduced the operating temperature of TiO_2_-based H_2_ sensors from 150 °C to 100 °C, indicating that conductive carbon or 2D additives can assist charge transport and improve low-temperature sensing performance.

Recent metal-oxide/MWCNT heterostructures also demonstrate that hierarchical oxide morphology can be coupled with CNT conduction pathways to improve low-temperature sensing. For example, flower-like Bi_2_SiO_5_/MWCNT nanocomposites showed selective room-temperature LPG sensing, with the optimized 10 wt% MWCNT composite reaching a 77.71% response to 1500 ppm LPG at 30 °C and response/recovery times of 21/37 s; this example further supports the broader strategy of using CNTs to build conductive heterojunction networks in oxide-based gas sensors [160].

A particularly recent example of CNT/2D-semiconductor interface engineering is the MWCNTs–InSe heterostructure reported by Gao et al. [54]. In this material, MWCNTs are distributed on the surface and edges of exfoliated InSe flakes, forming intimate interfacial contacts with the 2D semiconductor. The MWCNTs provide high electrical conductivity, abundant adsorption sites, and oxygen-containing surface groups, whereas InSe provides a gas-reactive semiconducting surface with temperature-sensitive carrier transport. Since MWCNTs generally behave as p-type conductors and few-layer InSe behaves as an n-type semiconductor, their contact forms interfacial p–n/Schottky-type junctions. DFT analysis showed charge transfer from InSe to MWCNTs, and the resulting built-in electric field facilitates charge separation and transport across the interface [54]. For NO_2_ sensing, the MWCNT/InSe interface is especially important because NO_2_ exhibits much stronger adsorption on the heterostructure than CO or H_2_, producing a larger resistance modulation. Thus, the enhanced sensing performance originates from the coupled effects of interfacial charge transfer, heterojunction-mediated barrier modulation, additional adsorption sites supplied by MWCNTs, and improved carrier transport along the hybrid network [54].

These recent hybrid interfaces further illustrate that high performance is achieved not by CNT functionalization alone, but by matching the functional component with an appropriate dominant mechanism, such as heterojunction modulation, adsorption-site engineering, humidity screening, or assisted charge transport.

## 6. Gas-Specific Sensing Characteristics

Carbon nanotubes have been applied to the detection of a broad spectrum of gases, but the published literature is strongly concentrated on a small number of analytes. Ammonia (NH_3_), nitrogen dioxide (NO_2_), and hydrogen (H_2_) are discussed first because they represent prototypical reducing, oxidizing, and weakly interacting gases for CNT networks. NH_3_ and NO_2_ exchange charge directly with p-type CNT frameworks, whereas H_2_ typically requires catalytic metals such as Pd or Pt to promote dissociation, spillover, or Schottky-barrier modulation. In response to the practical importance of additional toxic and greenhouse gases, this revised section further adds hydrogen sulfide (H_2_S) and carbon dioxide (CO_2_). H_2_S highlights the role of Cu/CuO, metal-oxide/CNT heterostructures, ion-gel enrichment, and flexible transistor amplification, while CO_2_ illustrates the need for amine-rich recognition layers, humidity management, baseline-drift control, and scalable printed platforms. Thus, the gas-specific discussion is organized not only by target species but also by the distinct sensing challenges and interface-design strategies required for practical CNT-based detection.

### 6.1. Ammonia (NH_3_)

Ammonia (NH_3_) is a major industrial chemical and agricultural emission, but its toxicity, corrosiveness, and low occupational exposure limit of 25 ppm over 8 h require reliable monitoring in environmental and workplace settings [141,161]. NH_3_ is also an important non-invasive breath biomarker, because abnormal exhaled concentrations are associated with impaired kidney function, Helicobacter pylori infection, halitosis, and metabolic disorders, whereas healthy breath generally contains only low-ppm levels of NH_3_ [39,141]. These applications require sensors with sub-ppm to ppm sensitivity, high selectivity against humidity and CO_2_, and safe operation at or near room temperature [39,161].

**Figure 17:**
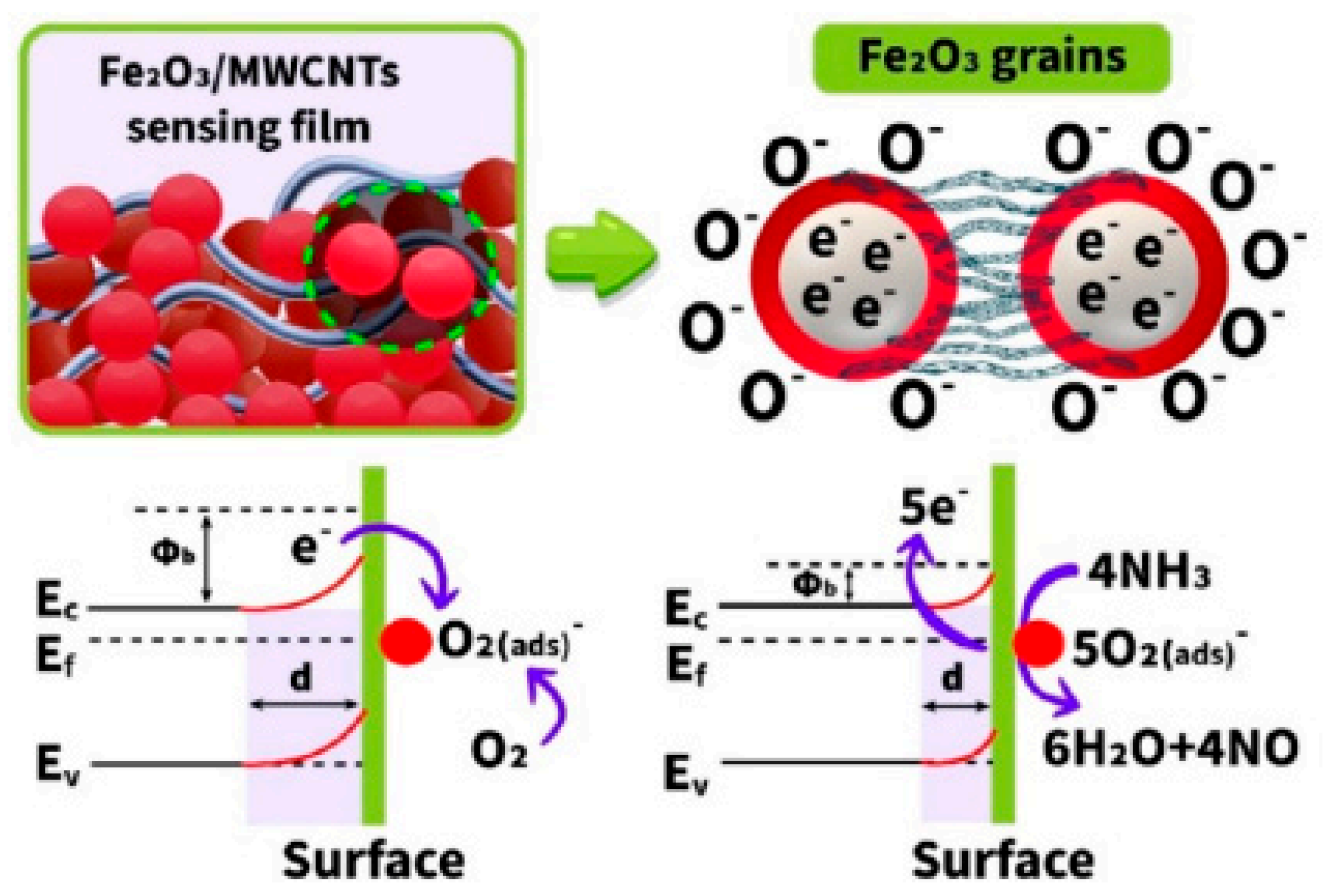
Ammonia-sensing mechanism for the Fe_2_O_3_/MWCNTs material. All panels adapted with permission from ref. [133].

In p-type CNTs, NH_3_ acts as an electron donor: adsorbed NH_3_ transfers electrons to the CNT network, reduces the majority hole concentration, and increases the resistance [39]. Although pristine CNTs can detect NH_3_ through this charge-transfer mechanism, their response and selectivity are often limited. CNT sensing layers are therefore commonly modified with acids, conducting polymers, oxygen- or sulfonate-containing groups, metal nanoparticles, metal oxides, graphene derivatives, or molecular receptors to introduce additional adsorption sites and enhance interfacial charge transfer. For example, Tanaka et al. mechanically interlocked SWCNTs with Cu(II)- and Co(II)-tethered nanobrackets, in which the coordinated metal ions served as selective NH_3_ binding sites. This molecular-receptor design enhanced sensitivity by more than one order of magnitude, reaching approximately 2.3% ppm^−1^, with a detection limit below 50 ppb and clear selectivity over H_2_, CH_4_, ethanol, and acetone [141]. Metal-oxide hybridization is also effective: Pt-decorated Fe_2_O_3_/MWCNT films prepared by e-beam deposition detected NH_3_ down to 3 ppm at 200 °C with response/recovery times of 40/25 s, where MWCNTs increased the effective surface area and provided conductive pathways between Fe_2_O_3_ nanograins [133]. As shown in Figure 17, the Fe_2_O_3_/MWCNT hybrids demonstrate that CNTs can function as conductive bridges and surface-area-enhancing components in oxide-dominated sensing films, improving charge transport and the NH_3_ response through interaction with oxygen species on Fe_2_O_3_ nanograins [133].

**Figure 18:**
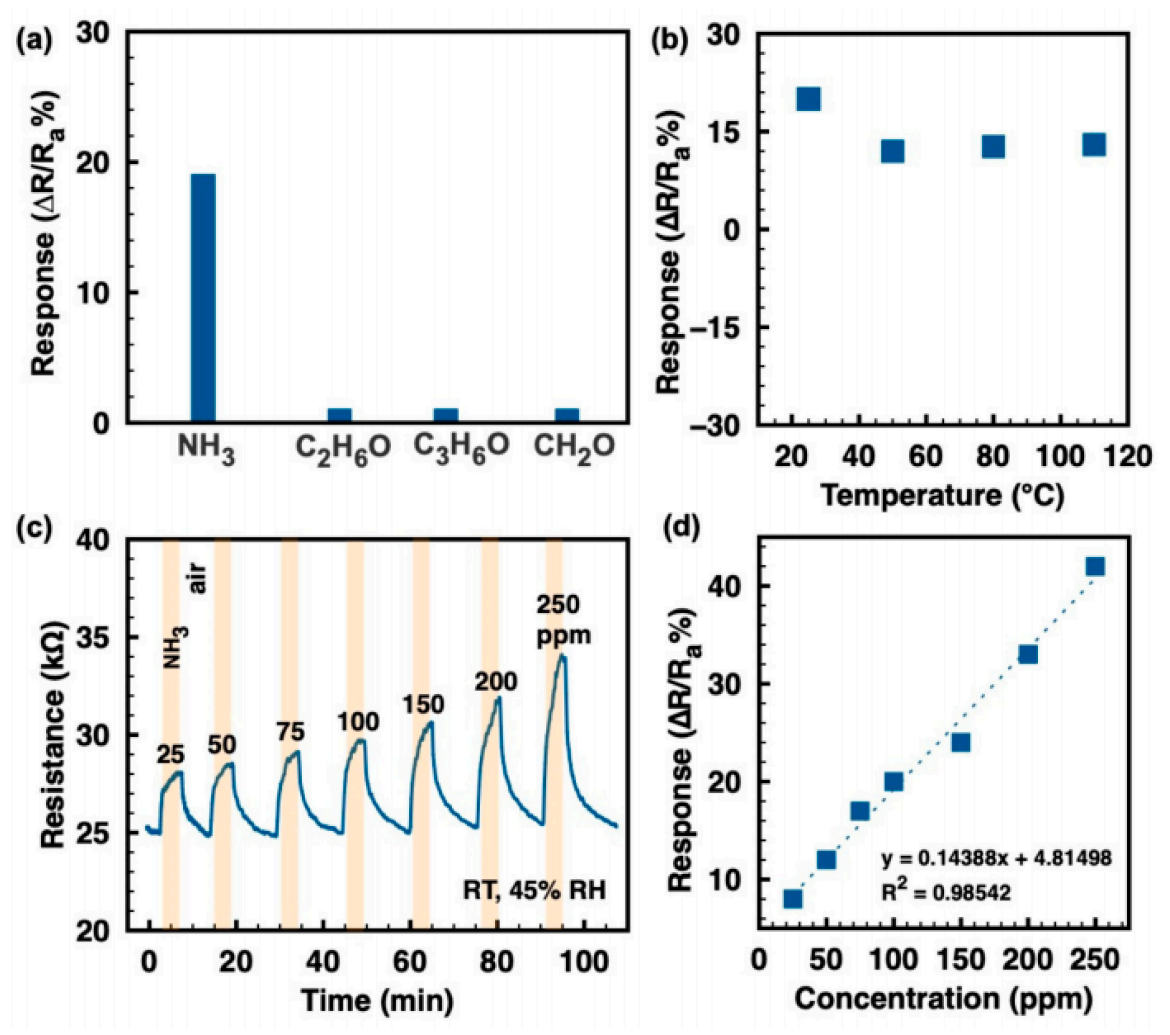
(**a**) Selectivity toward various gases (ammonia, ethanol, acetone, and formaldehyde) of 100 ppm at RT; (**b**) response values to 100 ppm ammonia at different working temperatures; (**c**) continuous resistance vs. time to ammonia at a variety of concentrations at RT; (**d**) the corresponding relationship of response values to ammonia and concentrations, with linear-fitting dashed lines. All panels adapted with permission from ref. [55].

Recent CNT-based NH_3_ sensors show that room-temperature performance can be improved by integrating CNTs with porous frameworks, selective polymer wrappers, conducting polymers, metal oxides, metal nanoparticles, or carbon–carbon junctions. ZIF-8/CNT composites are a representative example: hydrophobic ZIF-8 acts as an NH_3_ preconcentrator and humidity-screening layer, while CNTs form the conductive transduction network (Figure 18). The ZIF-8/CNT sensor showed selective room-temperature NH_3_ detection over ethanol, acetone, and formaldehyde, a response of approximately 20 to 100 ppm NH_3_, a linear response over 25–250 ppm, and an estimated LOD of 208 ppb; its response remained stable under moderate humidity of 45–70% RH but decreased at 90% RH because of competitive water adsorption [55]. Non-covalent polymer wrapping of SWCNTs provides another effective strategy. A Schiff-base polyphenylene polymer, FO-N-PA, was used to sort and functionalize semiconducting SWCNTs with >99% purity. The FO-N-PA@SWCNT sensor exhibited responses of 21.3% and 73.3% to 1 and 35 ppm NH_3_, respectively, with an LOD of 75.78 ppb and a 14-fold higher response than raw SWCNTs. The enhancement was attributed to semiconducting-SWCNT enrichment, polymer-induced adsorption sites, and imine groups that interact with basic NH_3_ molecules [40].

Conducting-polymer/CNT and metal-oxide/CNT composites further demonstrate the importance of interfacial engineering. PANI@Cu@MWCNT nanocomposites exhibited a much higher NH_3_ response than pure PANI at room temperature: among the tested compositions, PANI@Cu_3_@MWCNT_3_ showed the highest response of 116% to 100 ppm NH_3_, while PANI@Cu_2_@MWCNT_2_ provided rapid response/recovery times of 10/13 s [162].

The improvement originated from the combined effects of PANI deprotonation, high CNT conductivity and surface area, and Cu-assisted NH_3_ interaction. In WO_3_/CNT nanocomposites, CNT loading strongly affected the sensing behavior: optimized CNT contents enhanced the NH_3_ response through Schottky heterojunctions between CNTs and n-type WO_3_, whereas excessive CNT loading reduced the response because the opposite resistance-change directions of CNTs and WO_3_ partially compensated each other [56].

**Figure 19:**
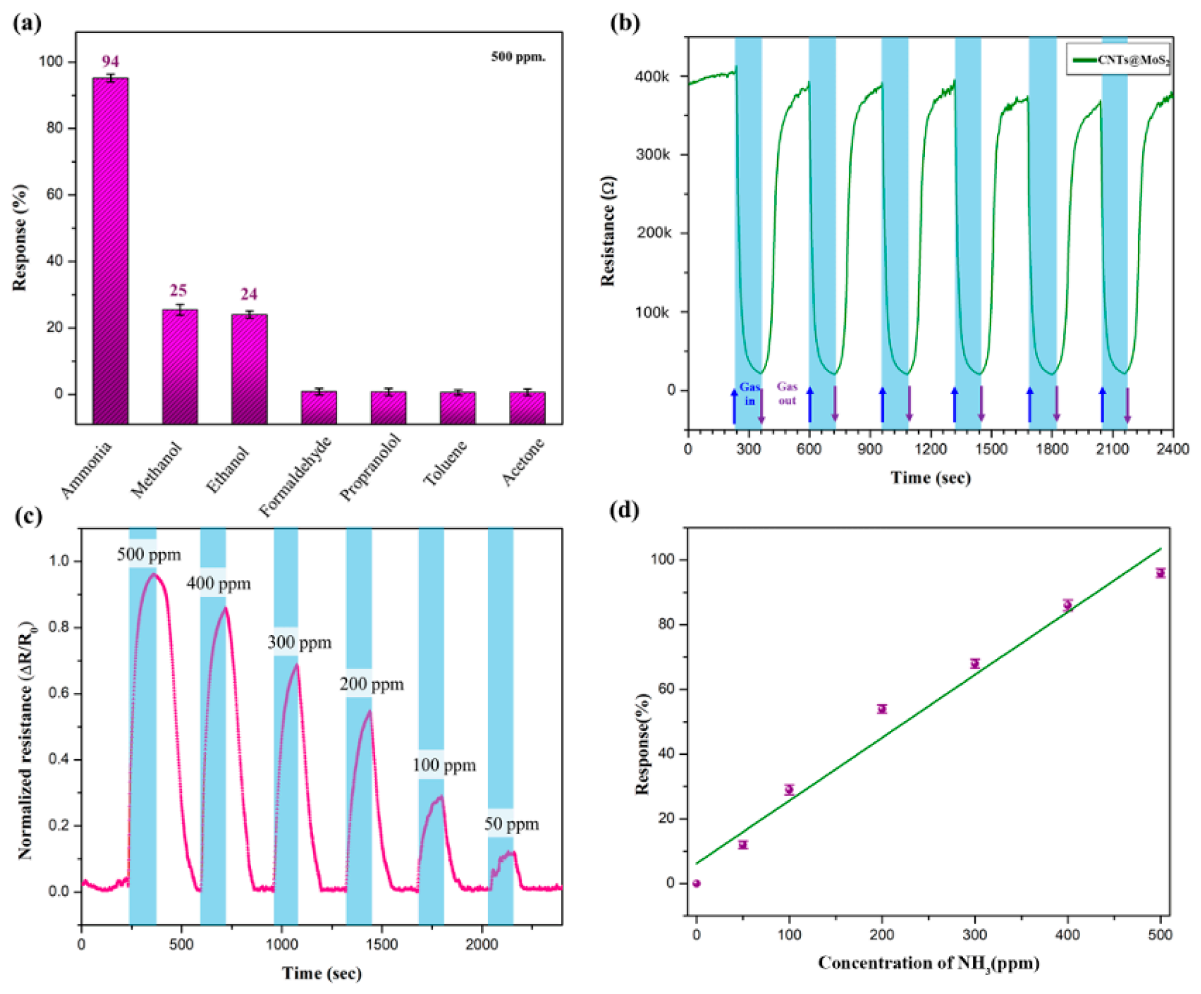
(**a**) Gas responses of CNTs@MoS_2_ gas sensors toward various VOCs (500 ppm) at room temperature. (**b**) Dynamic resistance response of CNTs@MoS_2_ gas sensor toward 500 ppm of NH_3_ at room temperature. (**c**) Responses of the CNTs@MoS_2_ gas sensor toward NH_3_ at varying concentrations (50−500 ppm) under room temperature. (**d**) Linear fitting curve of gas responses (%) versus NH_3_ concentrations. All panels adapted with permission from ref. [57].

CNT/TMD heterostructures have also been explored for room-temperature NH_3_ sensing. Seniwong-Na-Ayuttaya et al. reported a flower-like CNTs@MoS_2_ core–shell sensor, in which MoS_2_ nanosheets supplied NH_3_ adsorption sites and CNTs provided conductive pathways for charge transport [57] (Figure 19). The sensor showed a high response of 94% to 500 ppm NH_3_ at room temperature, with response/recovery times of 36/68 s, a detection limit of 10.33 ppm, and approximately 80% response retention after 150 days.

Metal and molecular decoration also enhance the CNT response: RF-sputtered In-decorated porous MWCNTs showed a maximum response of 144% to 10 ppm NH_3_, compared with 117% for pristine MWCNTs, with a rapid response within 1–7 s [163]. CuPc-functionalized SWCNTs also showed improved NH_3_ sensing through π-π stacking and additional p-type charge-carrier modulation, giving a 56% response enhancement at 40 ppm and more than 95% response under saturation conditions; however, recovery became slower because stronger NH_3_ interaction delayed desorption [41].

Despite these advances, slow or incomplete recovery remains a recurring issue. NH_3_ can bind strongly to defects, oxygenated groups, acidic sites, and receptor molecules, leading to baseline drift or partial recovery; mechanically interlocked SWCNT sensors, for instance, showed only partial recovery under ambient air purging, limiting their repeated use [141,164]. To address this, mild heating, UV or visible-light irradiation, electrical pulsing, DC biasing, self-heating, and increased purge flow have been employed to accelerate desorption [133,141]. Receptor design must therefore balance strong NH_3_ affinity for sensitivity with sufficiently reversible adsorption for recovery. Humidity is another critical factor: surface-adsorbed water can dissolve NH_3_ and promote formation of NH_4_^+^ and OH^−^, often increasing the response of carbon-based sensors, but excessive humidity may suppress NH_3_ adsorption through competitive H_2_O adsorption. Humidity-tolerant sensing layers are thus essential for breath and environmental monitoring [39,141].

### 6.2. Nitrogen Dioxide (NO_2_)

Nitrogen dioxide (NO_2_) is a major atmospheric pollutant generated mainly from fossil-fuel combustion, vehicle emissions, industrial processes, explosions, and welding. Even at low concentrations, long-term exposure to NO_2_ can cause serious respiratory complications and harms plants and terrestrial ecosystems [165,166]. Since ambient NO_2_ levels are strictly regulated, reliable monitoring is essential for air-quality control, environmental protection, and occupational safety [166]. Practical NO_2_ sensors therefore require high sensitivity at ppb–ppm levels, good selectivity against interfering gases and humidity, and stable operation under real environmental conditions; in particular, room-temperature NO_2_ detection has become an important target because it enables low-power, portable, wearable, and distributed sensing platforms [165,166].

Recent CNT-based NO_2_ sensors demonstrate that performance can be improved by controlling carbon morphology, interfacial structure, surface functional groups, and device architecture. Since NO_2_ is a strong oxidizing gas, it generally acts as an electron acceptor on p-type carbon networks: electron transfer from CNTs or carbon nanofibers to adsorbed NO_2_ increases the hole concentration and typically decreases the resistance of the sensing layer. However, the magnitude and reversibility of this response depend strongly on the density of adsorption sites, network conductivity, humidity, and the strength of NO_2_ binding.

Granulated carbon nanofibers (CNFs) provide a simple, low-cost carbon platform for room-temperature NO_2_ detection. Bannov et al. prepared non-treated granulated CNFs by methane decomposition over a Ni/Al_2_O_3_ catalyst and used them directly as a sensing material [167]. The sensor operated at 25 ± 2 °C over a broad NO_2_ range of 1–500 ppm and showed a response of 5.1% to 10 ppm NO_2_. The response decreased with increasing relative humidity, particularly at lower NO_2_ concentrations, because adsorbed water molecules occupied adsorption sites and hindered NO_2_ adsorption. The response to NH_3_ was more than two times lower than that to NO_2_, and CH_4_ produced almost no response, indicating reasonable selectivity; however, incomplete recovery suggested that both physisorption and partial chemisorption contributed to NO_2_ adsorption.

Earlier CNT-network sensors also showed that the type and defect chemistry of CNTs strongly affect NO_2_ sensing. Sayago et al. fabricated resistive sensors by airbrushing DWCNT, carboxylated DWCNT, and MWCNT networks on alumina substrates [168]. The sensors detected NO_2_ down to 0.1 ppm and showed much lower responses to H_2_, NH_3_, toluene, and octane. Among the tested materials, DWCNT-COOH networks exhibited the highest sensitivity, attributed to open end-caps, dangling bonds, and carboxyl-related defect sites. The NO_2_ response was maximized at 100–200 °C, whereas recovery was improved at 250 °C—a typical trade-off in CNT-based NO_2_ sensors, in which defects and functional groups enhance adsorption and sensitivity, but strong NO_2_ binding can slow recovery.

Three-dimensional CNT architectures have been introduced to overcome aggregation and increase the accessible sensing area. Ma et al. developed 3D SiO_2_@MWCNT core–shell nanospheres by electrostatic self-assembly of MWCNTs on APTES-modified SiO_2_ nanospheres [169]. This structure prevented severe MWCNT aggregation and generated an open, interconnected conductive network. The 3D SiO_2_@MWCNT sensor showed a high room-temperature response of approximately 82.6% to 1 ppm NO_2_—much higher than that of conventional 2D MWCNT networks—and UV-assisted recovery shortened the recovery time to about 44 s. The sensor also showed good repeatability, selectivity, and 30-day stability; the improved response was attributed to the effective utilization of the large MWCNT surface area and the increased number of accessible adsorption sites.

Flexible and hybrid carbon-based platforms have also been explored for room-temperature NO_2_ monitoring. Pagidi et al. incorporated acid-functionalized CNTs into polymer-dispersed liquid crystals (PDLCs) to fabricate an f-CNT-PDLC resistive sensor [170]. The f-CNTs provided conductive pathways and active NO_2_ adsorption sites, while the PDLC matrix improved mechanical stability and prevented deformation of the liquid-crystal phase. The device showed responses of approximately 12.9% and 1% to 100 and 5 ppm NO_2_, respectively, with a linear concentration dependence, five-cycle reproducibility, and selectivity against H_2_S, CO, H_2_, and NH_3_. Here, the sensing signal originated from charge transfer between NO_2_ and f-CNTs, together with orientational modulation of f-CNTs and liquid crystals inside the PDLC droplets.

Chemical doping is another effective strategy for increasing NO_2_ affinity. Tian et al. prepared highly nitrogen-doped SWCNT films through gas-phase fluorination followed by NH_3_ annealing [145]. The nitrogen content was tunable from 2.9 to 9.9 at%, and the optimized N-SWCNT film with 9.8 at% nitrogen showed a detection limit of 500 ppb at room temperature. The sensor operated at a very low voltage of 0.01 V and retained stable performance under bending, demonstrating its suitability for flexible and transparent wearable sensors. The enhancement was mainly attributed to pyridinic nitrogen sites, which provided active adsorption centers for NO_2_ while maintaining the conductive SWCNT network.

**Figure 20:**
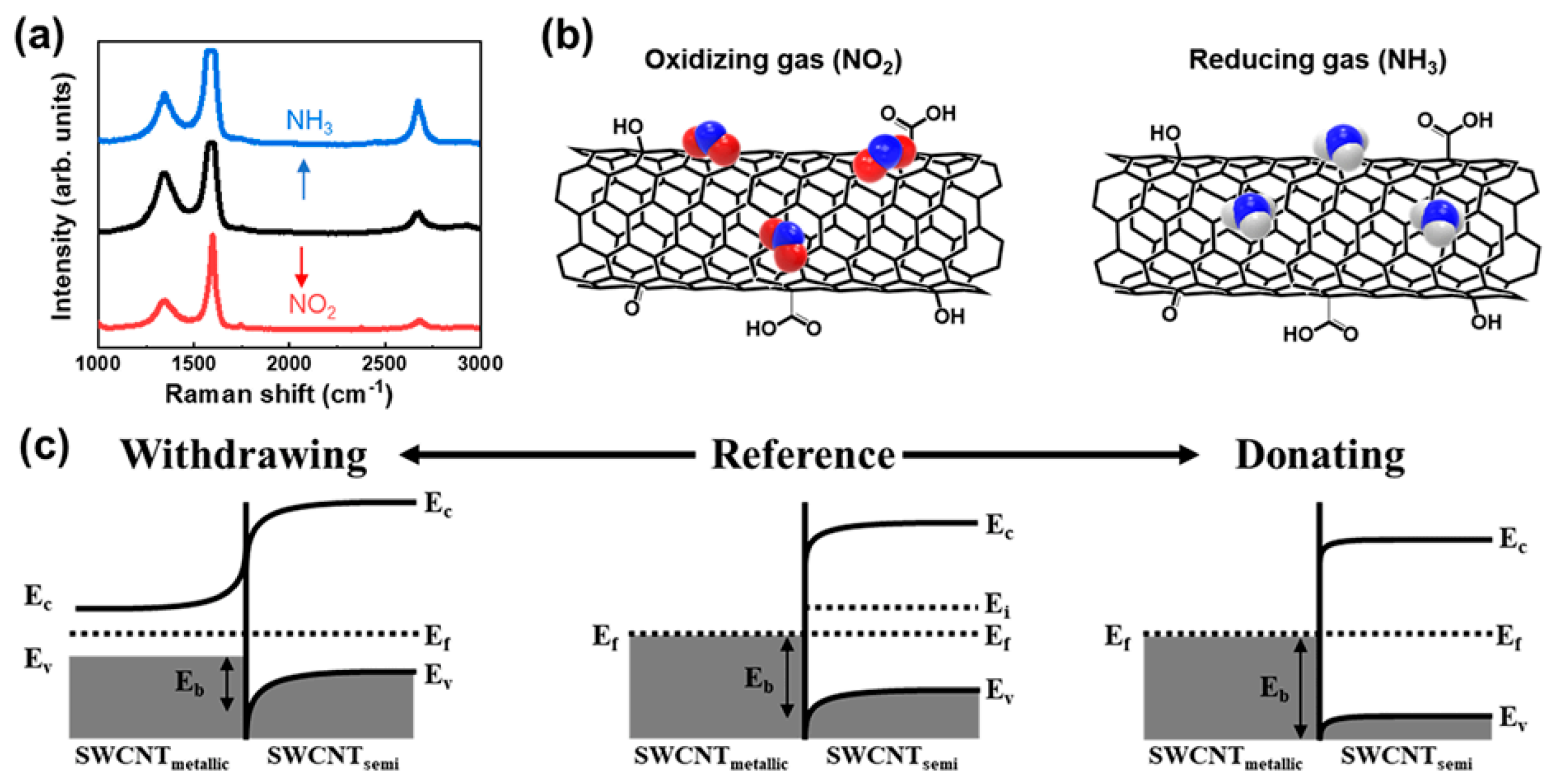
(**a**) Change in Raman spectra in the Oxy-SWCNT film after exposure to NO_2_ (red) or NH_3_ (blue). (**b**) Schematics of the adsorption of NO_2_ (**left**) and NH_3_ (**right**) onto Oxy-SWCNT, (**c**) change in band diagram in the junction between metallic and semiconducting SWCNT upon exposure to electron-withdrawing gas (**left**) and electron-donating gas (**right**). E_c_, E_i_, E_f_, E_v_, and E_b_ denote the conduction band, intrinsic Fermi level, Fermi level, valence band, and barrier height, respectively. All panels adapted with permission from ref. [146].

Substrate and surface engineering can further enhance the performance of SWCNT-based NO_2_ sensors. Hur et al. fabricated transparent, flexible, eco-friendly sensors by spray-coating oxidized SWCNT bundles on regenerated cellulose films [146]. The oxygen-functionalized outer shell of the SWCNT bundles improved solution processability and NO_2_ adsorption, while the inner pristine SWCNTs maintained efficient charge transport (Figure 20). Compared with SiO_2_-supported devices, cellulose-supported sensors showed an enhanced NO_2_ response because the polar glycosidic linkages in cellulose increased NO_2_ affinity. By adding halloysite nanotubes to increase cellulose surface roughness, the Oxy-SWCNT/HNT-cellulose sensor achieved an ultralow NO_2_ detection limit of 0.43 ppb. Although recovery was relatively slow, the combination of transparency, flexibility, biodegradability, and sub-ppb sensitivity makes this architecture attractive for wearable and disposable environmental sensors.

**Figure 21:**
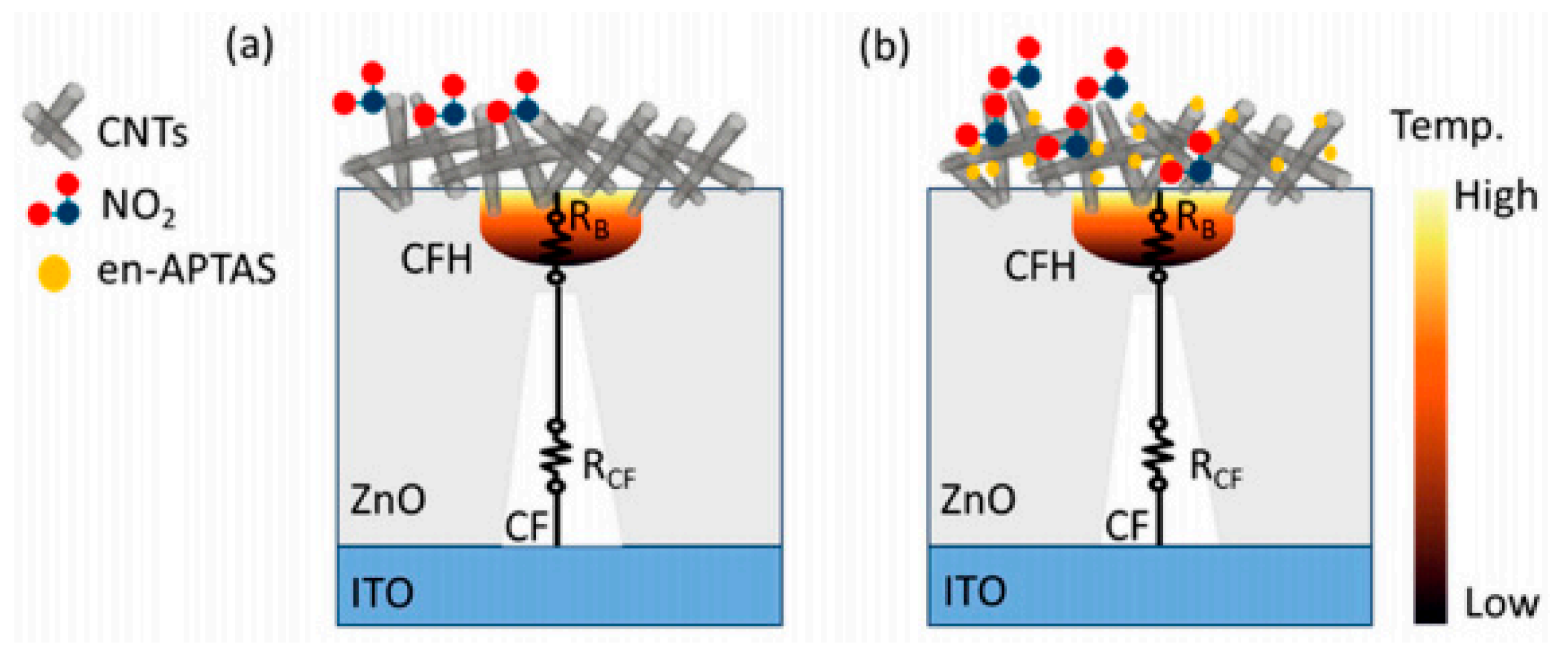
Schematics of interaction of NO2 gas with (**a**) CNTs and (**b**) en-APTAS-decorated CNTs. All panels adapted with permission from ref. [172].

Recent device-level approaches have focused on improving recovery and durability. Chae et al. integrated a transparent CNT gas sensor with an SnO_2_-based memristor heater [171]. The optimized 175 nm SnO_2_ memristor-heater/CNT device showed responses of 40.69% to 20 ppm and 19.42% to 0.5 ppm NO_2_ at room temperature. Compared with ZnO-based heaters, SnO_2_ provided better thermal stability and lower cycle-to-cycle variability, enabling stable operation over 50 cycles. Similarly, Ahmad et al. decorated CNT/ZnO/ITO memristor gas sensors with an en-APTAS membrane (Figure 21) [172]. The amine groups in en-APTAS increased NO_2_ adsorption sites and strengthened charge-transfer interactions, improving the response by about 60% at 20 ppm compared with the undecorated sensor. To overcome slow NO_2_ desorption, a −2.5 V pulse with a 1 ms width allowed the sensor to recover to baseline within 1 ms. These results show that local heating and pulse-recovery strategies can address one of the key limitations of CNT-based NO_2_ sensors.

The influence of film processing has also been studied systematically. Orlando et al. compared SWCNT films prepared with carboxymethyl cellulose (CMC) and sodium dodecyl sulfate (SDS) surfactants by spray deposition [173]. CMC produced thicker, more effectively dispersed SWCNT films than SDS, and the residual oxygen-containing groups influenced NO_2_ adsorption. The sensors showed improved NO_2_ responses under humid conditions and at elevated operating temperature, with the best performance obtained for 10-layer films at 150 °C and 40% RH—responses of 78.6% for CMC-SWCNT and 62.5% for SDS-SWCNT toward 2 ppm NO_2_. Cross-sensitivity tests further showed that heating to 150 °C improved selectivity toward NO_2_ over NH_3_, suggesting that both surfactant selection and mild thermal activation are important parameters in printed SWCNT sensor design.

### 6.3. Hydrogen (H_2_)

Hydrogen (H_2_) is attracting increasing attention as a clean energy carrier because it offers a high gravimetric energy density, rapid diffusion, and zero carbon emissions at the point of use [126,174]. It is expected to play an important role in decarbonizing transportation, industrial processes, power generation, and energy storage [126]. However, hydrogen is colorless and odorless, has a very low ignition energy, and forms explosive mixtures with air over a wide concentration range of 4–75 vol%, making accidental leakage difficult to recognize and potentially hazardous [174]. These risks are particularly critical in confined or semi-confined environments such as fuel-cell vehicles, storage systems, pipelines, and refueling stations [174]. Fast, selective, low-power, and reliable hydrogen sensors are therefore essential for the safe deployment of hydrogen technologies and the broader hydrogen economy [126,174].

Recent CNT-based H_2_ sensors show that the role of CNTs has expanded from simple conductive networks to multifunctional platforms that combine a gas-accessible surface area, mechanical flexibility, catalytic-metal interfaces, and Schottky-barrier modulation. Since pristine CNTs interact only weakly with H_2_ at room temperature, most high-performance devices rely on Pd or Pt decoration, surface functionalization, metal-oxide hybridization, microheater-assisted activation, or contact-engineered diode structures. In these systems, CNTs provide a high surface-to-volume ratio and efficient charge transport, while catalytic metals dissociate H_2_ molecules and convert hydrogen adsorption into a measurable resistance or current change.

**Figure 22:**
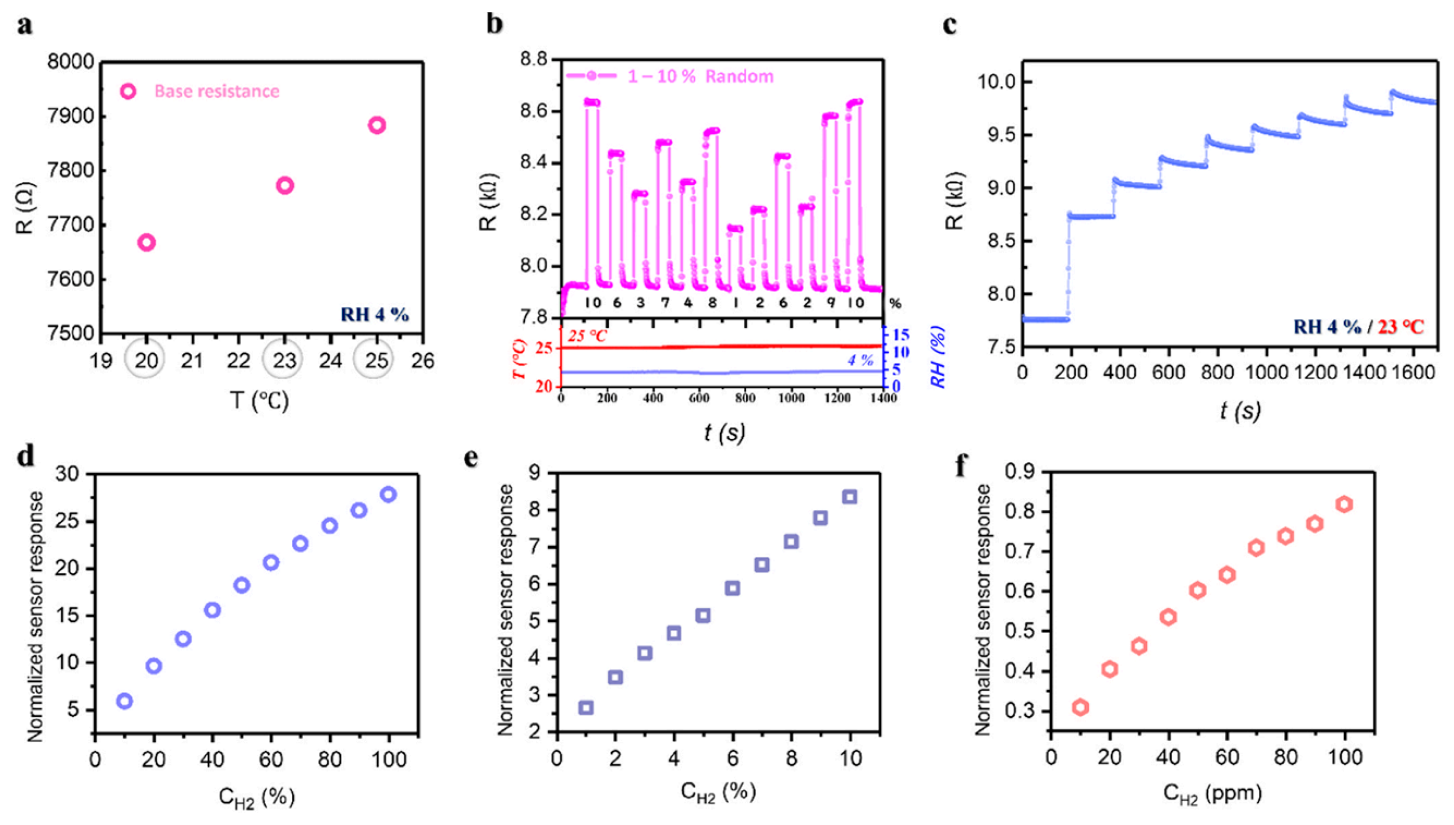
(**a**) Effect of temperature on the baseline resistance of the PdAu–PMP composite sensor. (**b**) Simultaneously measured resistance, temperature, and humidity responses of the sensor under randomly varying H_2_ exposure (H_2_ concentrations 1–10%). (**c**) Dynamic resistance response of the PdAu–PMP composite sensor to hydrogen concentration: 30–100%, demonstrating rapid and accurate operation even without an intermediate recovery period. (**d**–**f**) Normalized response of the sensor as a function of H_2_ concentration in different concentration ranges: (**d**) 10–100%, (**e**) 1–10%, and (**f**) 10–100 ppm. All panels adapted with permission from ref. [27]. Sensors and Actuators B: Chemical.

Pd-based CNT composites remain highly attractive for H_2_ sensing because Pd dissociates H_2_ and forms palladium hydride, but pure Pd often suffers from phase-transition-induced hysteresis, volume expansion, and long-term instability at high H_2_ concentrations. Han et al. developed a wide-range PdAu–PMP composite sensor consisting of intense-pulsed-light-treated PdAu alloy electrodes and a Pd/MWCNT@PVP nanocomposite sensing layer [27]. In this architecture, Pd nanoparticles on the MWCNT network promote surface catalytic H_2_ dissociation and charge transfer, while the PdAu alloy electrodes suppress the α–β phase transition of Pd and enable stable bulk hydrogen absorption. The sensor operated at room temperature over a broad H_2_ range from 10 ppm to 100%, with an estimated LOD of approximately 1 ppm and rapid response/recovery times of 3/8 s at 50% H_2_, 7/14 s at 5% H_2_, and 15/36 s at 50 ppm H_2_. The device also showed high selectivity (Figure 22), reproducible responses under 4–85% RH, and oxygen-independent operation, making it promising for H_2_ pipelines, storage tanks, fuel-cell systems, and explosion-prone environments [27].

A more controlled route to room-temperature H_2_ detection was demonstrated using chemically immobilized monolayer semiconducting SWCNTs. Girma et al. fabricated uniform ≈1 nm sc-SWCNT monolayers by a click reaction between azide-functionalized polymer-wrapped sc-SWCNTs and alkyne-functionalized substrates, followed by Pd-nanoparticle decoration and PMMA coating [175]. The click-assembled SWCNT films showed much better reproducibility than spin-coated films because the CNT density and network morphology were chemically controlled. Pd thickness was a critical parameter: insufficient Pd provided too few H_2_ adsorption sites, whereas excessive Pd formed additional conductive pathways and reduced modulation of the SWCNT channel. With optimized 2 nm Pd and an anisole-derived PMMA selective layer, the sensor achieved a high room-temperature response of 285 to 1% H_2_ in air, with response/recovery times of 10/3 s. The PMMA coating also improved selectivity against CO, CO_2_, CH_4_, and humidity by acting as a molecular barrier while allowing efficient H_2_ diffusion [175].

Low-cost CNT synthesis and deposition methods have also been explored for simple chemiresistive H_2_ sensors. Al-Diabat et al. synthesized MWCNTs using a conventional microwave oven, functionalized them with nitric acid, and deposited them between Pd electrodes on SiO_2_ substrates by dielectrophoresis [176]. The sensor showed an unusually high room-temperature sensitivity of 315% to 140 ppm H_2_ and retained a measurable response even at 20 ppm. The high performance was attributed to acid-induced –COOH and –OH groups, improved CNT dispersion, increased adsorption sites, and efficient electron transfer from H_2_ to the CNT network. In a related study, untreated CNTs and HNO_3_-treated CNTs were directly compared [58]: the functionalized CNT sensor showed a much higher response of 368% over 20–300 ppm H_2_, with response and recovery times of 15 and 72 s, respectively, whereas untreated CNTs showed inferior transport and sensing properties. These results indicate that even without noble-metal nanoparticle decoration, defect and functional-group engineering can substantially enhance room-temperature CNT/H_2_ interactions [58,176].

Device-level heating provides another strategy to activate H_2_ sensing in pristine CNTs. Lee et al. directly integrated spray-coated MWCNTs onto a suspended Pt microheater fabricated by MEMS processing [59]. Since pristine CNTs show little H_2_ response at room temperature, the Pt microheater locally raised the sensing temperature while maintaining a low power budget. The device began to respond above approximately 170 °C, and the response increased with temperature, reaching 1.82% to 10% H_2_ at 420 °C, with response and recovery times of 39 and 35 s and an estimated LOD of 1200 ppm. Raman spectra before and after sensing showed little degradation of the CNT layer, confirming the structural stability of the integrated sensor. Although the sensitivity was lower than that of Pd-functionalized CNT sensors, this work demonstrates that CNTs can be incorporated into MEMS platforms for low-power, miniaturized H_2_ sensing without additional catalytic decoration [59].

CNT/metal-oxide hybridization can improve sensitivity by combining the conductivity and porosity of CNTs with the surface redox activity of metal oxides. Abdulhameed and Mahnashi developed CNT/TiO_2_ H_2_ sensors using a two-stage dielectrophoresis process [60]. In conventional single-stage deposition, simultaneous CNT/TiO_2_ assembly increased sensitivity but also raised the resistance because TiO_2_ interrupted CNT conduction pathways. In contrast, the two-stage process first aligned CNTs across the electrodes and then deposited TiO_2_ nanoparticles on top of the CNT network. This structure preserved a low resistance of approximately 524 Ω while increasing the room-temperature sensitivity to 15.78% at 1000 ppm H_2_, compared with less than 2% for CNT-only sensors and 3.42% for single-stage CNT/TiO_2_ deposition. The sensing mechanism was attributed to H_2_ reaction with chemisorbed oxygen on TiO_2_ and functionalized CNT sites, producing water and releasing electrons that increase conductance. This work highlights that the fabrication sequence and nanoscale percolation control are as important as material selection in CNT-composite sensors [60].

Schottky-contact engineering offers a highly sensitive alternative to conventional chemiresistive sensing. Dang et al. reported a wafer-scale SWCNT-film diode H_2_ sensor using Pd/Cr/Pd trilayer contacts [28]. The dangling-bond-free surface of SWCNTs weakens Fermi-level pinning, allowing the Schottky barrier at the Pd/CNT interface to respond strongly to Pd work-function changes during PdH_x_ formation. Compared with single Pd contacts, the Pd/Cr/Pd trilayer improved stability because the intermediate Cr layer relieved stress associated with Pd lattice expansion during repeated H_2_ absorption and desorption. The optimized sensor showed a response of 12.56 to 5000 ppm H_2_ with a response time of 22 s, stable operation over repeated cycles and at high humidity, and excellent selectivity against CH_4_, CO, ethanol, SO_2_, NO_2_, and NH_3_. Importantly, the sensor was integrated into a system for monitoring H_2_ evolution during lithium-ion battery thermal runaway, demonstrating the practical relevance of CNT-based H_2_ sensors for early-warning safety systems [28].

**Figure 23:**
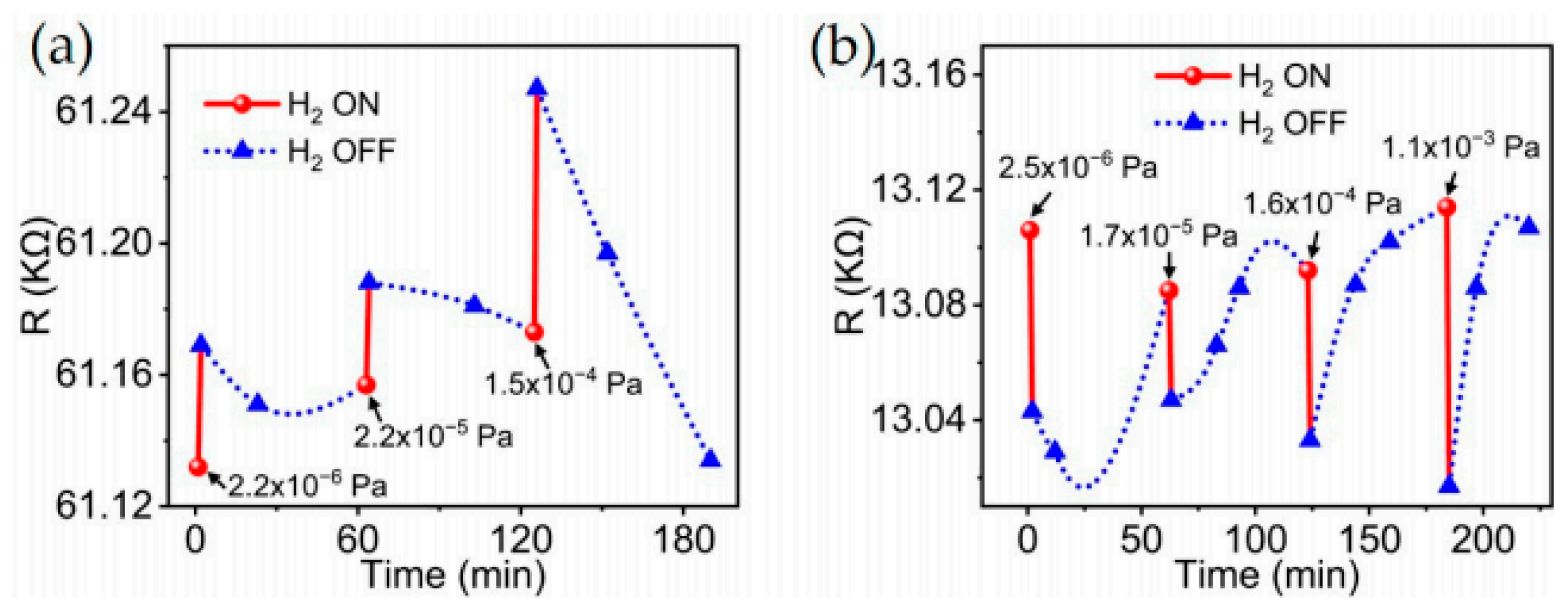
The hydrogen sensing response: (**a**) MWCNT/Pd sensors and (**b**) MWCNT/Au sensor. All panels adapted with permission from ref. [61] MDPI.

The importance of CNT–metal Schottky interfaces was further clarified by Huang et al., who compared MWCNT/Pd and MWCNT/Au junctions under extremely low H_2_ pressures of 10^−6^–10^−3^ Pa [61]. The two devices exhibited opposite resistance trends: resistance increased in MWCNT/Pd sensors but decreased in MWCNT/Au sensors upon H_2_ exposure (Figure 23). This behavior was attributed to different work-function changes in the metal and CNT surfaces after hydrogen adsorption, which altered the Schottky barrier and carrier transport at the metal–CNT junction. The study is important because it shows that CNTs should not be regarded only as chemically inert conductors; hydrogen adsorption can also modify CNT electronic properties and thereby influence contact-dominated sensing signals. These findings provide a useful design rule for CNT devices in which contact resistance, rather than channel resistance alone, governs the H_2_ response [61].

CNT/graphene hybrid electrodes decorated with Pt nanoparticles provide another route for improving charge transport and signal amplification. Alamri et al. investigated Pt-nanoparticle/CNT/graphene nanohybrids prepared by atomic layer deposition and compared SWCNT, DWCNT, and MWCNT films [62]. In this architecture, Pt nanoparticles catalyze H_2_ dissociation, CNT films provide a large sensing area, and graphene serves as a high-mobility charge-transport channel that reduces losses at CNT–CNT junctions. Among the tested CNTs, MWCNTs with a diameter of approximately 10 nm gave the best performance because they balanced an accessible surface area with higher electrical conductivity. The Pt-NPs/MWCNT/graphene sensor showed a response of approximately 26% to 10% H_2_, which increased to 36% after 5 min of UV irradiation. UV treatment likely removed adsorbed air molecules from the CNT/graphene surfaces and opened additional adsorption sites. Although the response decreased in air compared with N_2_, oxygen-assisted desorption improved recovery, suggesting that ambient operation can be beneficial for practical cycling [62].

The fundamental sensing mechanism of Pd/SWCNT back-to-back Schottky contacts was examined by Zhang et al. [29]. In this device, aligned SWCNTs bridged Pd microelectrodes, forming a chemiresistive/ChemFET structure. Upon H_2_ exposure, Pd absorbs hydrogen and forms PdH_x_, reducing the Pd work function and modulating the Schottky barrier height at the Pd–SWCNT interface. Since the current through a Schottky barrier depends exponentially on the barrier height, small H_2_-induced work-function changes generate large current or resistance changes. The sensor detected 50–2000 ppm H_2_ at room temperature, and its response followed a logarithmic dependence on H_2_ concentration. Comparative measurements with Pt, Cr, and Au electrodes showed that Pd/SWCNT contacts provided the most favorable combination of sensitivity, reversibility, and response behavior. The I–V and FET analyses confirmed that Schottky-barrier modulation, rather than a simple carrier-density change in the SWCNT channel, was the dominant sensing mechanism [29].

### 6.4. Hydrogen Sulfide (H_2_S)

Hydrogen sulfide (H_2_S) is a highly toxic, corrosive, and flammable gas associated with petroleum refining, coal processing, wastewater treatment, chemical production, and biological decomposition. Its occupational exposure limit is typically in the low-ppm range, while ppb-level monitoring is desirable for environmental and early-warning applications. For CNT sensors, H_2_S detection is challenging because pristine CNT networks usually show a limited and poorly selective response; practical sensing therefore relies on catalytic metals, copper-based chemistry, metal-oxide/CNT heterostructures, or transistor amplification to convert H_2_S adsorption or reaction into a larger electrical signal [44,47,177,178,179].

**Figure 24:**
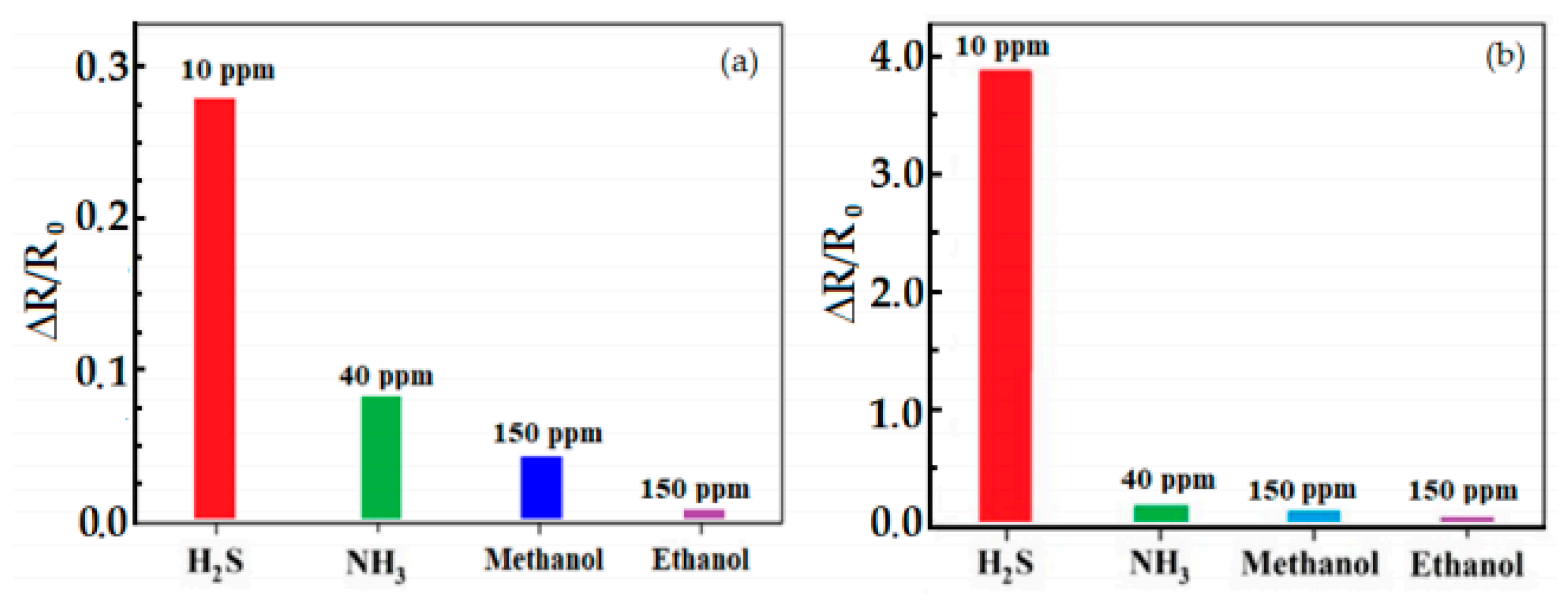
Selectivity of the sensors, prepared by (**a**) physical and (**b**) chemical methods. All panels adapted with permission from ref. [177].

Cu-containing CNT composites are particularly important because copper and copper oxides have strong chemical affinity toward H_2_S. Asad and Sheikhi demonstrated room-temperature wireless H_2_S detection using CuO-SWCNT hybrid nanomaterials, showing that CuO decoration can provide selective H_2_S reaction sites while SWCNTs serve as the conductive transduction network [44]. Musayeva et al. further compared physical and chemical Cu deposition on pristine and oxygen-functionalized MWCNTs. In their f-MWCNT/Cu nanocomposites, oxygen-containing functional groups improved Cu anchoring and gas interaction, and the chemically deposited f-MWCNT/Cu structure exhibited approximately five times higher H_2_S sensitivity, reaching about 400% ΔR/R_0_ at room temperature, than the physically deposited analogue (Figure 24) [177].

Recent flexible and heterostructured CNT sensors further extend H_2_S detection toward low-power and ppb-level operation. CNT/Fe_2_O_3_ films have been reported for flexible ppb-level H_2_S sensing at room temperature [47]. Geng et al. used printed ion-gel CNT thin-film transistors in which the ion gel enriched H_2_S and the transistor architecture amplified the signal; the device showed a response of 4045.5% to 2 ppm H_2_S, a sensitivity up to 1565% ppm^−1^, response/recovery times of 10/26 s, ultralow power consumption of 1.03 nW, and stable operation after 10,000 bending cycles [179]. More recently, a multilevel CNT-CuO-ZnO film combined the flexible CNT network with CuO/ZnO heterostructures and CuO-H_2_S reaction chemistry, achieving a response of 50% to 300 ppb H_2_S, a measured LOD of 20 ppb, and 96.8% response retention after bending [178]. These examples show that H_2_S sensing benefits from coupling CNT conductivity and flexibility with copper-based reaction sites, oxide heterojunctions, and device-level signal amplification (Figure 24).

### 6.5. Carbon Dioxide (CO_2_)

Carbon dioxide (CO_2_) is a major greenhouse gas and an important indicator for indoor air quality, occupational safety, environmental monitoring, and breath analysis. Unlike NO_2_ or NH_3_, CO_2_ is chemically stable and interacts only weakly with pristine CNT sidewalls; therefore, direct charge transfer from CO_2_ to bare CNTs is generally insufficient for reliable room-temperature sensing. CNT-based CO_2_ sensors usually require receptor layers that introduce specific acid–base or hydration-assisted interactions, most commonly amine-rich polymers such as polyethyleneimine (PEI), amidine-containing polymers, or hybrid amine/CNT composites [43,67,180,181,182,183,184,185].

Early flexible CNT CO_2_ sensors showed that uniform CNT films can detect CO_2_ at room temperature. Lin et al. transferred CVD-grown CNT thin films onto polyimide substrates and reported an immediate response, with 2.23% sensitivity at 800 ppm CO_2_, demonstrating the feasibility of flexible CNT-film CO_2_ sensing [180]. Kumar and Yadav synthesized MWCNT thin films by direct liquid-injection CVD and reported room-temperature CO_2_ sensing with a response of 2.1, 98% reproducibility, and response/recovery times of 30.2/49.6 s, supported by theoretical calculations of CO_2_ adsorption on CNTs [181]. However, because pristine CNT-CO_2_ interaction remains weak, receptor-functionalized CNTs are generally required for a stronger and more selective response.

Amine-functionalized CNT sensors directly address this limitation. Han et al. fabricated PEI-functionalized CNT thin films by vacuum filtration, in which PEI wrapping introduced primary, secondary, and tertiary amine groups capable of interacting reversibly with CO_2_ at room temperature; the PEI-CNT sensor reached about a 4.2% response at 1000 ppm CO_2_ and showed an improved response compared with pristine CNTs, as well as repeatability, selectivity, and flexibility [67]. Yoon et al. introduced a CO_2_-switchable SWCNT-polymer composite containing amidine groups that convert to amidinium bicarbonate under humid CO_2_ exposure, providing reversible conductance modulation and selectivity over O_2_ and Ar [182]. These demonstrate that CO_2_ sensing in CNT networks is governed less by direct CNT adsorption and more by receptor-mediated charge modulation or carrier compensation.

**Figure 25:**
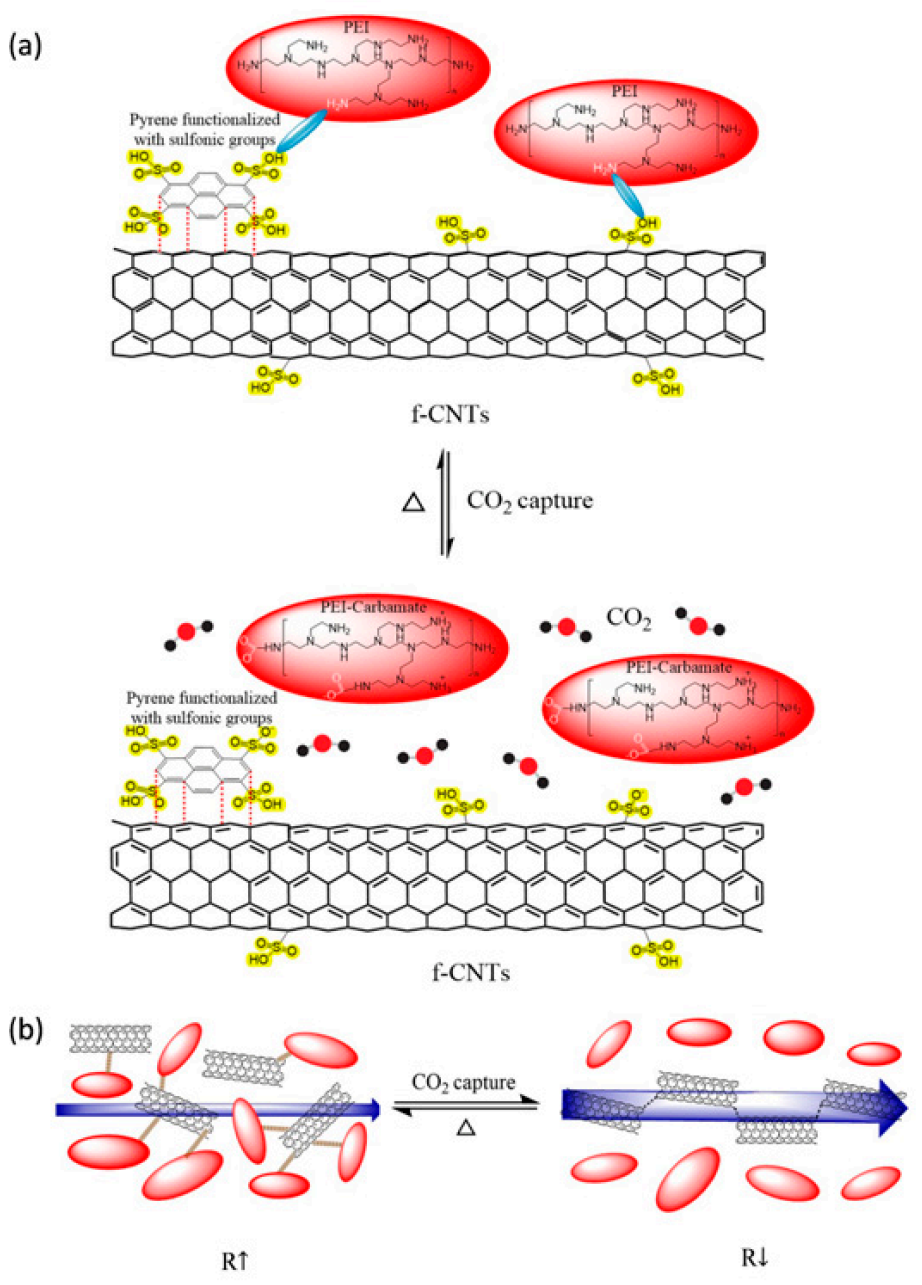
(**a**) Chemical structure of f-CNTs with PEI before and after CO_2_ capture. (**b**) Schematic illustration of the sensing mechanism. All panels adapted with permission from ref. [183].

Recent studies emphasize scalability, drift, humidity compensation, and long-term reliability. Liu et al. developed screen-printable f-CNT/PEI composites in which pyrene and sulfonate modification improved CNT dispersion in PEI, enabling flexible and scalable CO_2_ sensors with a detection range of 300–5000 ppm and systematic assessment of ink dilution and humidity effects (Figure 25) [183].

**Figure 26:**
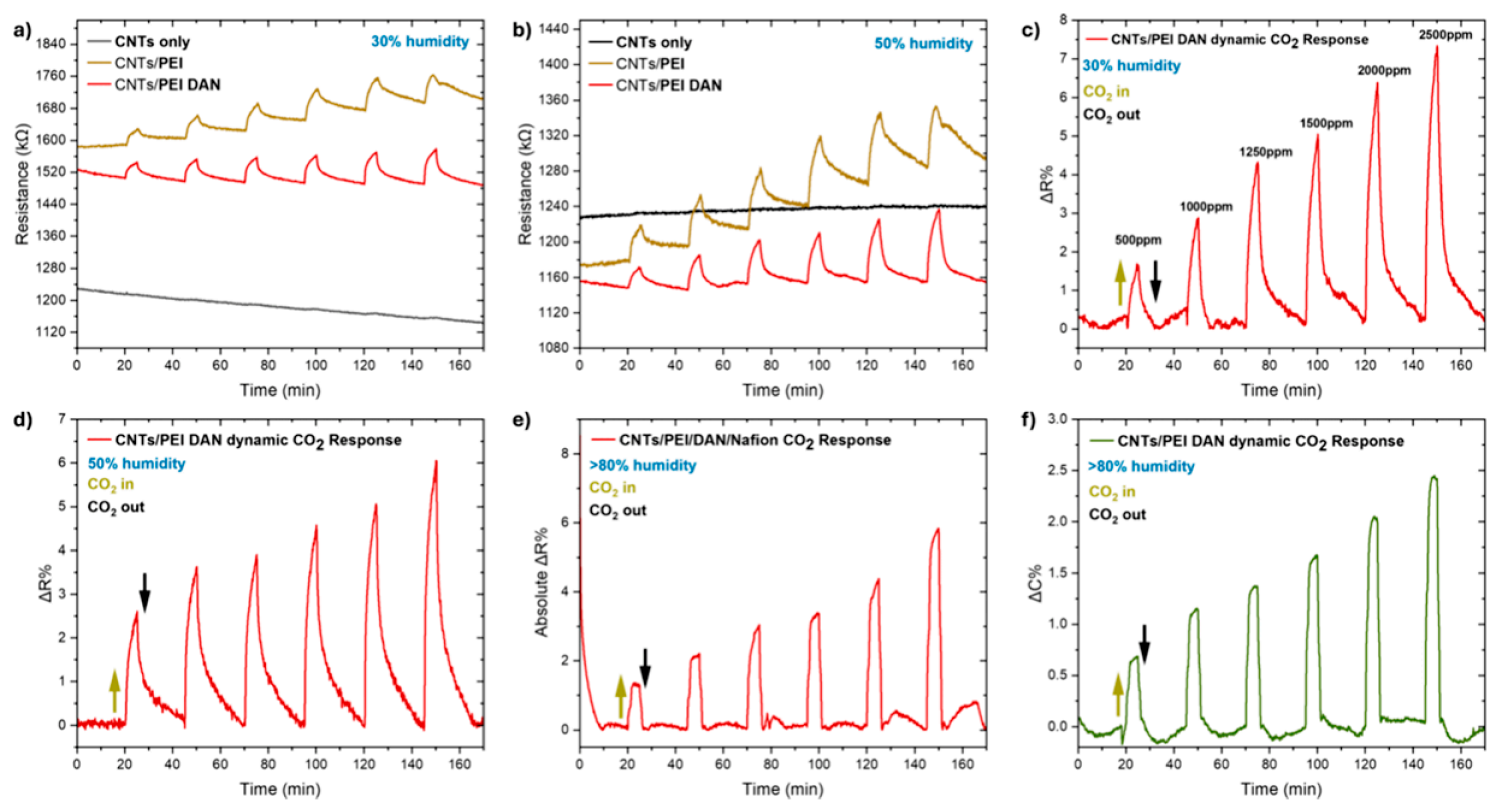
Signal drift mitigation after DAN mixing with PEI at (**a**) 30% and (**b**) 50% relative humidity (RH). Dynamic resistance response to sequential CO_2_ pulse of the CNT/PEI/DAN sensor at (**c**) 30% and (**d**) 50% relative humidity (RH). (**e**) Real-time resistance response to increasing CO_2_ concentrations. (**f**) Real-time capacitance response under the same CO_2_ exposure conditions. All panels adapted with permission from ref. [185].

A subsequent study showed that high-molecular-weight PEI/f-CNT composites improved thermal and cycling stability, providing a 300–2000 ppm CO_2_ detection range and robust operation over 40 continuous cycles [184]. Most recently, Tzourmpakis et al. reported a drift-minimized COOH-f-SWCNT/PEI/DAN sensor with a Nafion layer for dual-mode humidity compensation. The device detected 500–2500 ppm CO_2_ with a sensitivity of 2.67 × 10^−3^ ppm^−1^, a 7.5% response at 2500 ppm under 30% RH, improved batch-to-batch reproducibility, and stable indoor/outdoor and breath-monitoring demonstrations [185]. These advances show that practical CNT-based CO_2_ sensing requires not only amine chemistry but also control of baseline drift, humidity, recovery kinetics, reproducibility, and scalable fabrication (Figure 26).

The CNT network provides electrical transduction, while PEI or switchable polymer receptors promote reversible CO_2_ interaction under appropriate humidity conditions, leading to resistance modulation. Recent CO_2_ sensors move beyond response enhancement toward scalable printed f-CNT/PEI inks, stabilized amine chemistry, Nafion-assisted humidity compensation, drift-minimized operation, and real-world indoor/outdoor or breath-monitoring demonstrations. The comprehensive comparison in Table 6 summarizes representative NH_3_, NO_2_, H_2_, H_2_S, and CO_2_ sensors selected from Section 6. To make the comparison more useful for assessing practical reliability, the table now includes response/LOD information, response/recovery kinetics where reported, selectivity or humidity tolerance, stability/repeatability/durability, and the dominant sensing mechanism.

## 7. Flexible and Wearable CNT-Based Gas Sensors

Wearable and flexible sensors require sensing layers that simultaneously satisfy electrical conductivity, mechanical compliance, stability, fast response, user comfort, and compatibility with integrated electronics. Carbon-based materials, particularly CNTs, are well suited to this requirement because their high aspect ratio, large accessible surface area, low density, chemical stability, and excellent flexibility allow conductive networks to be formed on polymer films, fibers, fabrics, paper, and mask substrates. In flexible sensor systems, CNTs can act not only as active chemiresistive channels but also as stretchable conductors, porous scaffolds, ion-to-electron transducers, and interfacial bridges that improve charge transport in hybrid materials. Recent progress in carbon-based flexible sensors also shows that device-level design, including textile integration, self-powered operation, and wireless readout, is becoming as important as the sensing material itself [63].

Recent studies demonstrate several routes for translating CNT gas sensors from rigid substrates to wearable platforms. These include direct coating of CNT composites on porous mask fibers, continuous spinning of weavable CNT fibers, ultrasonic nanosoldering of graphene/CNT hybrids onto non-woven fabrics, laser writing of porous graphene electrodes combined with CNT composites, self-powered SWCNT films (Figure 25), CNT-based electrodes for sweat analysis, CNT-coated cotton fabrics for toxic gas detection, wireless polymer/MWCNT sensor arrays, MoS_2_-SWCNT hybrid films on PET, and PVA-wrapped MWCNT networks on fabrics [64,65,66,67,68,69,70,134,186,187,188].

A representative example of breath-compatible NH_3_ sensing is the p-PP/CNT/PANI sensor fabricated directly on the porous polypropylene layer of a disposable surgical mask [64]. In this device, CNTs were first deposited onto the mask fibers to form conductive pathways, followed by in situ polymerization of PANI as the NH_3_-sensitive layer. The resulting p-PP/CNT/PANI network combined the high surface area of the porous mask substrate, the conductivity of CNTs, and the protonation/deprotonation chemistry of PANI. The optimized sensor operated at room temperature and detected NH_3_ from 500 ppb to 70 ppm with a response of 452% to 70 ppm NH_3_ and response/recovery times of 93/36 s. It also showed high selectivity over CO_2_, CO, and several VOCs, reliable bending tolerance, 20-day stability, and real-time detection of different breathing patterns when integrated into a surgical mask. This work is important because it links chemical NH_3_ sensing with respiratory-rate monitoring in a simple, disposable wearable format.

Textile integration can also be achieved by using CNTs themselves as continuous sensing fibers. Rohilla et al. reported additive-free, continuous CNT fibers produced by floating-catalyst CVD and collected at a high production rate of approximately 339 m h^−1^ [65]. The fibers exhibited high porosity, high electrical conductivity, about 16% strain-to-failure, a Young’s modulus of approximately 1.4 GPa, and sufficient mechanical robustness to be stitched into commercial polyester fabric. Without additional functionalization, the CNT fiber textile detected VOCs such as methanol, ethanol, acetone, and benzene, as well as toxic gases including H_2_S, with an approximate detection limit of 50 ppb and response times below 30 s for most VOCs. For H_2_S, a 4.6% resistance change was observed at 10 ppm, although NO and H_2_S required longer response times of about 200 s. The sensing performance was maintained under bending, twisting, and knotting, demonstrating that macroscopic CNT fibers can serve as both wearable conductors and gas-sensitive elements.

Wearable gas sensors must also preserve comfort, breathability, and washability when incorporated into clothing. Although the rGO/CNTs/NWF e-textile reported by Yao et al. was developed primarily as a strain and pressure sensor rather than a gas sensor, it provides useful design principles for wearable CNT gas-sensor substrates [66]. The ultrasonic nanosoldering process strongly attached CNTs and rGO onto non-woven fabric, forming a conductive three-dimensional hybrid network that remained gas-permeable and superhydrophobic. The rGO/CNTs/NWF sensor showed gauge factors from 2.2 to 80.3 under tensile strain and pressure sensitivities up to 1.21 kPa^−1^, while maintaining stable responses over 2000 tensile or compressive cycles and preserving conductance after prolonged washing. These results indicate that strong interfacial anchoring of CNT/graphene networks is critical for wearable gas sensors that must operate under repeated deformation, perspiration, washing, and air-flow conditions.

For VOC monitoring, Lu et al. developed a flexible room-temperature methanol sensor based on laser-induced graphene (LIG) electrodes coated with a CoPc/MWCNT composite [186]. LIG provided a three-dimensional porous electrode with abundant diffusion pathways, MWCNTs supplied efficient electron-transport channels, and CoPc introduced selective interaction sites through its metal center and π-conjugated structure. The optimized four-layer LIG-CoPc/MWCNT sensor detected methanol over 10–1000 ppm with a sensitivity of 0.589 ohm ppm^−1^ and an LOD of 165 ppb, covering occupational exposure limits for methanol. It showed a rapid initial response (tau45 = 5 s), a recovery time (tau85 = 108 s), low hysteresis of 2.77%, high selectivity over ethanol, isopropanol, acetone, ammonia, formaldehyde, and acetaldehyde, and only 4.25% performance degradation over 30 days. This architecture illustrates how CNTs can be combined with molecular receptors and laser-patterned carbon electrodes to achieve low-power, flexible VOC sensing at room temperature.

**Figure 27:**
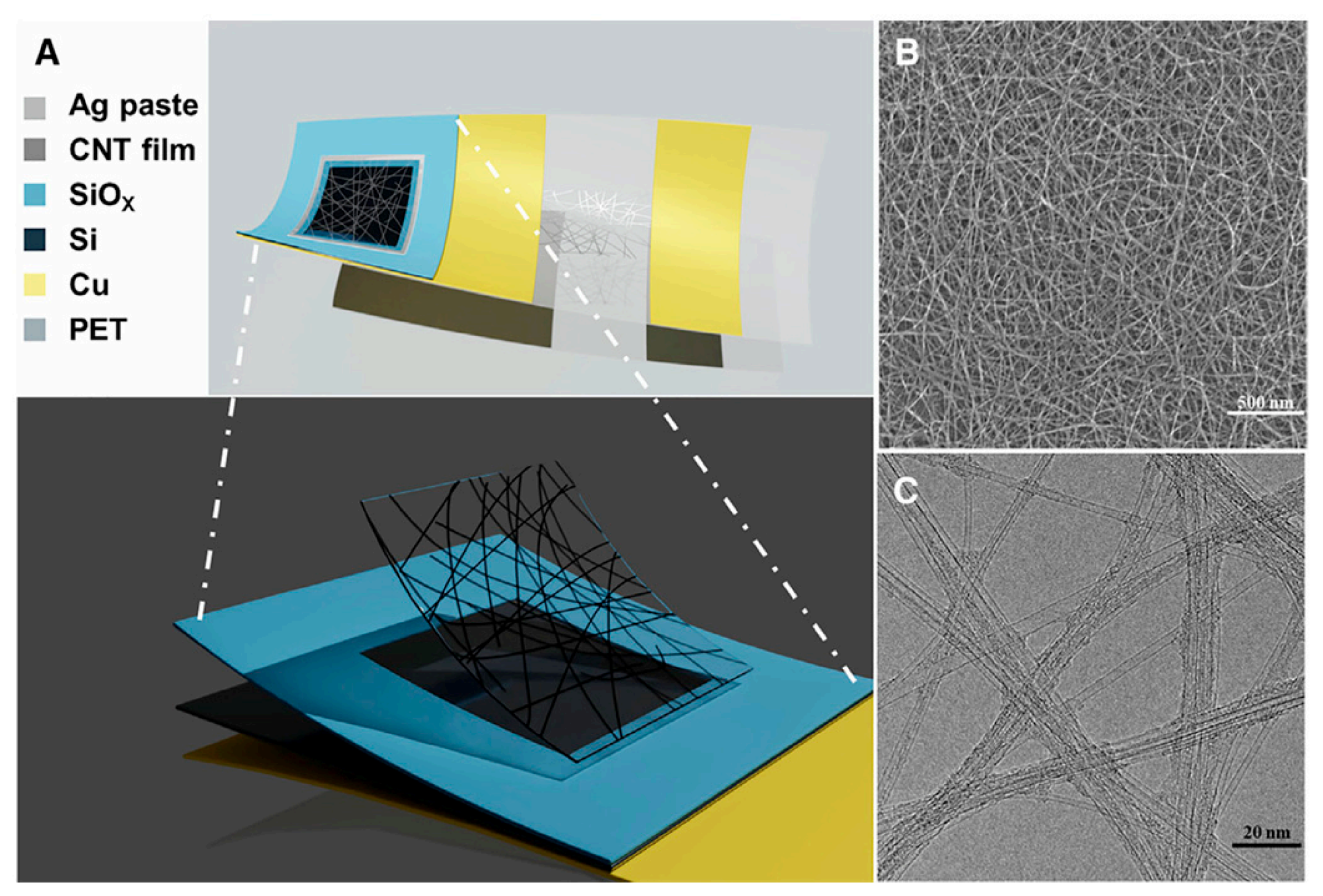
A self-powered flexible gas sensor based on SWCNT films. (**A**) Schematic of the flexible, self-powered gas sensor, which comprises an SWCNT film detector and an SWCNT/Si heterojunction solar cell. (**B**,**C**) Typical (**B**) SEM and (**C**) TEM images of the SWCNT films used in the devices (scale bars: SEM, 500 nm, and TEM, 20 nm). All panels adapted with permission from ref. [134].

**Figure 28:**
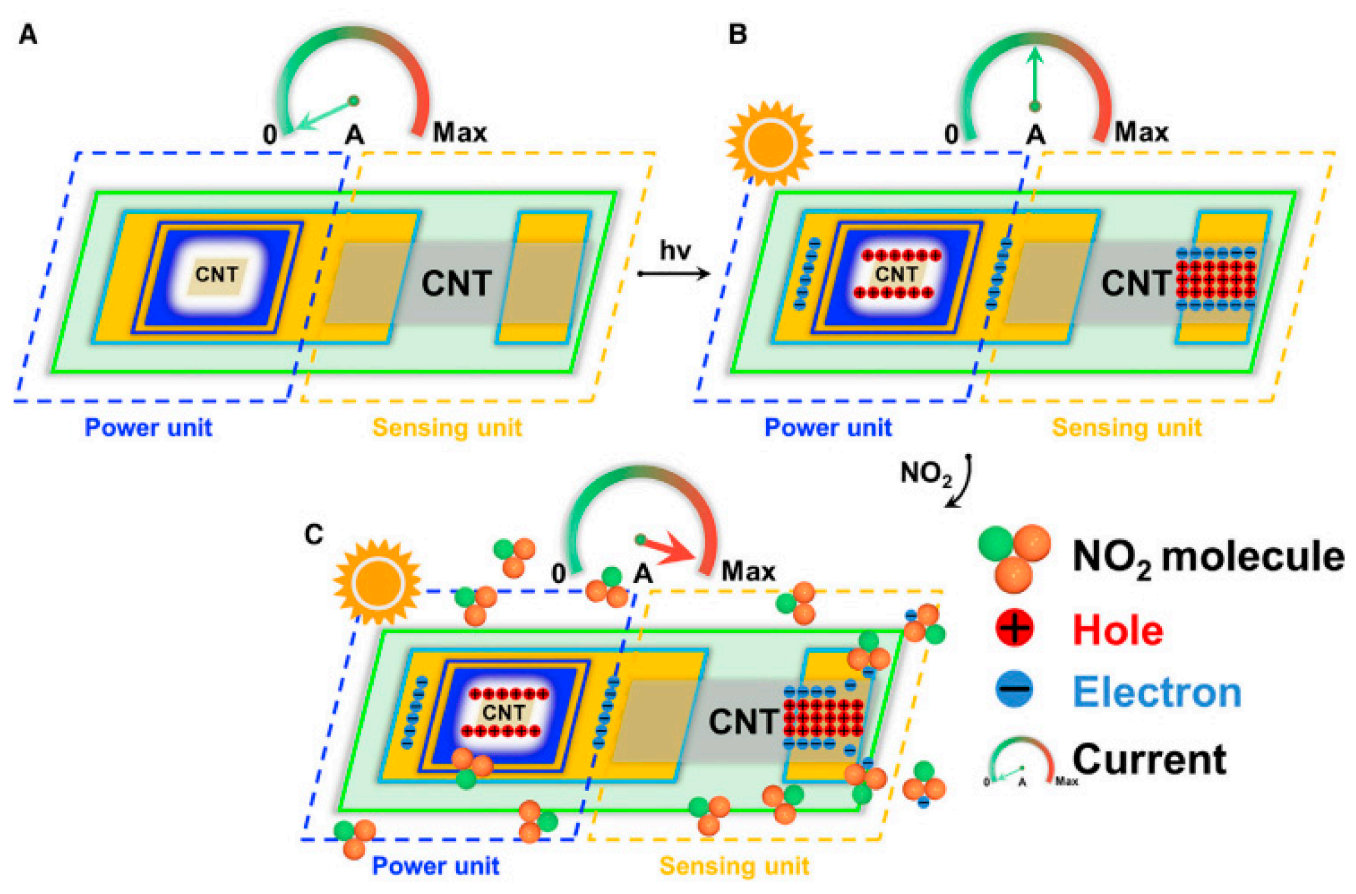
Schematic showing the working mechanism of the self-powered flexible sensing system. (**A**) Original dark stage. (**B**) Stage under illumination. (**C**) Exposure to NO_2_. All panels adapted with permission from ref. [134].

Self-powered operation is particularly attractive for distributed wearable sensors because it reduces dependence on batteries and external wiring. Guo et al. demonstrated a flexible NO_2_ sensing system in which SWCNT films served both as the sensing unit and as a component of an SWCNT/Si heterojunction (Figure 27) [134]. The self-powered device detected NO_2_ down to 1 ppm at room temperature and showed an approximately 72 s response time to 100 ppm NO_2_. Compared with an externally powered SWCNT sensor, the integrated self-powered system improved the sensitivity and response time by about 230% and 330%, respectively, because both the sensing unit and power unit participated in NO_2_-induced carrier modulation (Figure 28). The device retained sensing ability after 500 bending cycles and was coupled with a Bluetooth Low Energy module for wireless alarm notification, showing a practical route toward autonomous wearable gas-sensing nodes.

Although the CNT-based ion-selective electrode arrays reported by Roy et al. are not gas sensors, they are relevant to wearable CNT transducer design [187]. In this system, a plasticized PVC ion-selective membrane containing sodium ionophore infiltrated porous CNT films, producing stronger mechanical attachment than on flat Au, Pt, or carbon electrodes. The complete flexible Na^+^ sensing platform showed a near-Nernstian sensitivity of 58 ± 3 mV decade^−1^ and stable open-circuit potential for at least 1 h. This example is therefore cited only as a wearable chemical-sensing platform that illustrates how porous CNT networks can serve as mechanically compliant, electrochemically stable transducers for future integrated breath, sweat, and exposure-monitoring systems.

**Figure 29:**
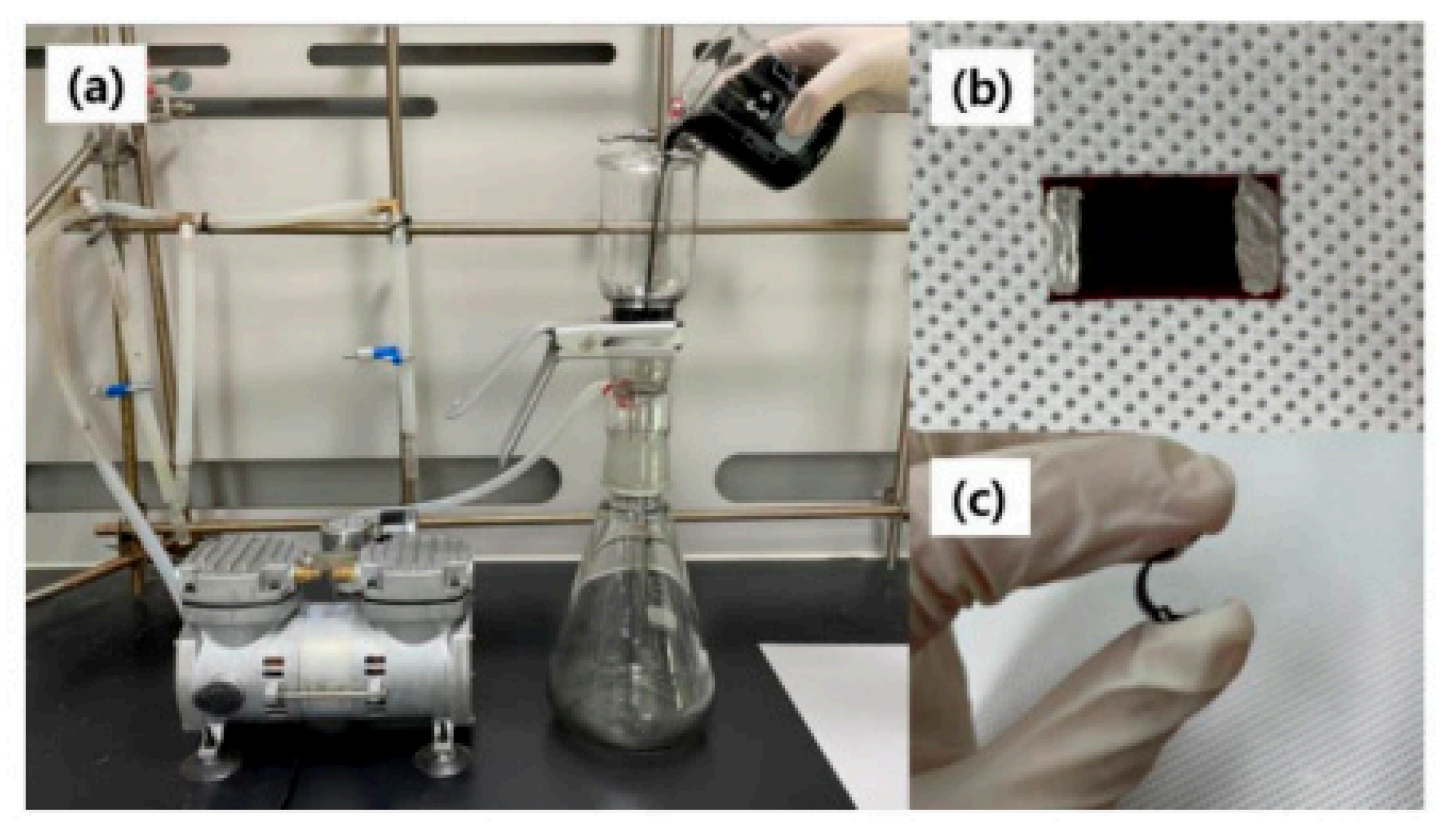
(**a**) Filtration system; (**b**) the image of the fabricated sensor; (**c**) the flexibility of the sensor. All panels adapted with permission from ref. [67].

CO_2_ is difficult to detect at room temperature because it is chemically stable and interacts weakly with pristine CNTs. Han et al. addressed this limitation by non-covalently functionalizing a vacuum-filtered CNT thin film with polyethyleneimine (PEI), whose primary, secondary, and tertiary amine groups react reversibly with CO_2_ to form carbamate or bicarbonate-related species [67]. PEI wrapping increased the CNT diameter from about 10 to 16 nm without significantly damaging the graphitic structure, and the dense filtered CNT network provided a uniform and flexible resistive channel on a polyimide substrate (Figure 29). The PEI-CNT sensor showed a concentration-dependent response to 200–1000 ppm CO_2_ at room temperature, reaching about 4.2% response at 1000 ppm in nitrogen and significantly outperforming pristine CNTs. Humidity enhanced the response, reaching approximately a twofold increase at 1000 ppm CO_2_ under 80% RH, while repeatability, five-day stability, selectivity over O_2_, CO, H_2_, and CH_4_, and a bending test confirmed its potential for flexible CO_2_ monitoring.

Carbon monoxide detection has also been demonstrated using a low-cost textile platform. Kumar et al. fabricated MWCNT-coated cotton fabric sensors by a repeated dip-and-dry process using SDS-dispersed functionalized MWCNTs [68]. Increasing the MWCNT concentration from 0.3 to 0.5 mg mL^−1^ produced denser conductive networks and reduced sheet resistance from 17,457 to 2217 Ω sq^−1^. The optimized CCM 5 sensor detected CO from 25 to 100 ppm at room temperature, with responses of 9.11% at 25 ppm and 15.2% at 100 ppm, and showed a response time below 49 s. The sensor also exhibited repeatability, reproducibility, selectivity over methane and hydrogen, a low estimated material cost of about 0.3 USD for a 2 cm × 0.5 cm device, and lightweight biodegradable textile compatibility. However, complete recovery was not achieved without external heating, UV illumination, or vacuum treatment, indicating that desorption remains a key issue for CNT-coated fabric CO sensors.

Wireless readout and temperature stabilization are also important for moving CNT-based flexible sensors toward practical wearable systems. Chiou and Wu developed a wearable and wireless gas-sensing system based on a flexible polymer/MWCNT composite sensor array on a polyimide flexible substrate [69]. Eight polymer/MWCNT sensing elements based on PVP, PEO, EC, and PMS were integrated with interdigitated electrodes, a microcontroller-based readout circuit, a Bluetooth Low Energy module, and a smartphone application. A double-spiral heater and RTD were embedded on the backside of the flexible array to maintain a thermostat temperature of 40 °C and reduce ambient-temperature effects, with a heater power consumption of approximately 210 mW. The system generated distinct response patterns toward 1000 ppm NH_3_, 1000 ppm NO_2_, and 10 ppm toluene, demonstrating that polymer/MWCNT arrays can combine semi-selective gas recognition, wireless data transmission, and real-time smartphone display in a wearable air-pollution monitoring platform [69].

Hybridizing CNTs with two-dimensional semiconductors provides another route to flexible and transparent wearable chemical sensors. Kim et al. fabricated MoS_2_-SWCNT hybrid films by growing MoS_2_ nanosheets on spin-coated SWCNT networks and transferring the hybrid layer to PET substrates [70]. The SWCNT network increased charge transport, gas-accessible adsorption sites, and mechanical robustness, while the MoS_2_ nanosheets supplied a semiconducting sensing surface. At room temperature, the MoS_2_-SWCNT sensor showed responses of approximately 54% to 40 ppm NO_2_ and 34% to 40 ppm NH_3_, higher than pristine MoS_2_ films. The transferred hybrid film retained a high optical transmittance of 92.83% in the visible range, and the MoS_2_-SWCNT device maintained a stable resistance and sensing performance after 10^5^ bending cycles, whereas the resistance of pristine MoS_2_ sensors increased to approximately 300% under the same bending process [70].

Fabric-based CNT/polymer composites further highlight the potential of directly wearable gas sensors. Maity et al. fabricated a room-temperature ethanol sensor by spray layer-by-layer coating of MWCNTs followed by PVA on commercially available cotton fabrics [188]. PVA wrapping improved ethanol selectivity through hydroxyl-group interactions and swelling-mediated modulation of the MWCNT inter-tube contact resistance. The PVA/MWCNT fabric sensor showed a nearly 13-fold higher response than bare MWCNT fabrics, a linear response over 100–500 ppm ethanol, an estimated LOD of 9.17 ppm, and fast response/recovery times of 24 ± 2 s and 29 ± 3 s at room temperature. It also maintained stable baseline resistance under forward and reverse bending up to 60° and showed higher selectivity to ethanol than ammonia, acetone, benzene, cyclohexane, methanol, toluene, and xylene, making it a relevant example of a low-cost wearable textile gas-sensing platform [188].

Together, these additional examples show that flexible and wearable CNT gas sensors should be assessed not only by gas response but also by system-level factors such as substrate breathability, bending durability, temperature compensation, wireless readout, power consumption, and compatibility with textile or mobile-device integration.

Table 7 summarizes representative flexible and wearable CNT-based sensing platforms discussed in Section 7. The selected examples are intended to compare platform design and wearable relevance rather than provide an exhaustive catalogue.

## 8. Challenges and Future Perspectives

Although CNT-based gas sensors have made substantial progress in sensitivity, miniaturization, room-temperature operation, and mechanical flexibility, several challenges must still be addressed before their widespread practical deployment. In this section, the remaining challenges are discussed according to practical deployment requirements: humidity tolerance, selectivity against interfering gases, response/recovery kinetics, reproducibility and long-term stability, power consumption, and system-level integration.

### 8.1. Humidity Effects and Moisture-Tolerant Interfaces

Humidity remains one of the most important practical limitations of CNT-based gas sensors. Water molecules can adsorb on CNT sidewalls, defect sites, oxygen-containing functional groups, polymer coatings, and metal-oxide or two-dimensional-material interfaces, thereby altering baseline resistance, competing with target-gas adsorption, and inducing false-positive or false-negative signals. This issue is particularly critical for room-temperature, wearable, breath-related, and environmental sensors because they operate under continuously varying relative humidity rather than under dry laboratory conditions.

Recent studies indicate that humidity tolerance can be improved through both material-level and signal-level strategies (Table 8). Edge-rich WSe_2_/MWCNT composites provide a representative material-design route: WSe_2_ supplies abundant reactive edge sites for NO_2_ adsorption, whereas MWCNTs increase conductivity, introduce hydrophobic pathways, and form nanoscale interfacial barriers. The optimized WSe_2_/MWCNT composite showed a 1.85-fold higher response to 1 ppm NO_2_ than pristine WSe_2_ at 100 °C, a theoretical LOD of 2.8 ppb, and stable cyclic and long-term operation even at 80% RH [52]. Similarly, WS_2_/MWCNT composites enabled room-temperature dual sensing of NH_3_ and NO at temperatures down to 18 °C and 90% RH. In that system, the hydrophobic nature of MWCNTs reduced the effect of relative humidity, while the WS_2_/MWCNT interface enabled opposite resistance responses to NH_3_ and NO, supporting discrimination under humid conditions [147].

Device and readout strategies are also important. WO_3_/CNT flexible NO_2_ sensors illustrate how CNTs can improve conductivity and interfacial charge transfer in oxide-based sensors, while integrated electronics enable real-time data acquisition, wireless transmission, and mobile display [189]. In another approach, Falco et al. demonstrated dynamic self-compensation of humidity in printed CNT devices by measuring impedance at different frequencies. The 1 MHz response isolated moisture-related contributions and enabled gas-specific humidity compensation without using an additional humidity sensor [143]. These examples show that moisture tolerance cannot be solved by one universal method; rather, it requires combining hydrophobic interfaces, selective adsorption sites, a controlled operating temperature, differential/reference channels, and calibration or algorithmic compensation.

### 8.2. Selectivity and Interfering Gas Responses

Selectivity is another central challenge because pristine CNTs usually exhibit a broad resistive response to many electron-donating or electron-withdrawing molecules. This broad sensitivity is advantageous for detecting small perturbations in the surrounding atmosphere, but it becomes problematic when humidity, oxygen, CO_2_, VOCs, NH_3_, NO_2_, H_2_S, CO, and other gases coexist. Practical CNT gas sensors therefore require either chemically selective functional interfaces or array-based discrimination strategies rather than relying only on a single nonspecific resistance signal.

Functionalization with catalytic nanoparticles, metal oxides, conducting polymers, biomolecular receptors, MOFs, TMDs, and ionic liquids can improve selectivity by introducing analyte-specific adsorption sites, redox activity, acid/base interactions, molecular sieving, or hydrogen-bonding interactions. For example, WS_2_/MWCNT composites enabled discrimination between NH_3_ and NO by producing opposite resistance responses and NO-dominated signals during simultaneous exposure [147]. MOF/MWCNT composites offer another selectivity route because MOFs provide adsorption sites with different electron affinities while MWCNTs provide electrical transport [50]. Future CNT-sensor reports should therefore include interference tests under realistic gas mixtures, quantitative selectivity factors, concentration-dependent cross-response maps, and RH-dependent selectivity rather than only single-gas measurements in dry air.

### 8.3. Response/Recovery Kinetics and Reversibility

Fast response and complete recovery are essential for real-time monitoring, repeated exposure cycles, and wearable operation. However, CNT-based gas sensors often exhibit slow or incomplete recovery because target molecules bind strongly to defects, oxygen-containing groups, polymer domains, catalytic nanoparticles, metal-oxide interfaces, or porous hybrid layers. This problem becomes more pronounced at room temperature, where thermal desorption is limited. Thus, adsorption strength must be balanced with reversibility: maximizing the response alone can produce high sensitivity but poor cycling stability.

Several strategies have been proposed to improve recovery, including mild heating, UV or visible-light illumination, electrical pulsing, self-heating, hydrophobic coatings, optimized chamber flow, and interface engineering. Low-power localized heating is particularly attractive because it can accelerate desorption without continuously heating the entire sensor substrate. Suspended CNT sensors demonstrated that self-heating can recover NO_2_-exposed nanotubes at microwatt-level power, reducing the recovery time by orders of magnitude compared with a room-temperature purge alone [72]. More recent filament-heater-integrated transparent CNT sensors showed a dynamic NO_2_ response with a sensing power of 6.15 µW and pulse-assisted recovery below 1 ms at 30.8 µW, while reducing humidity-induced response variation [190]. These results indicate that recovery should be discussed together with power consumption and humidity robustness rather than as an isolated kinetic metric.

### 8.4. Reproducibility, Long-Term Stability, and Standardized Reporting

Reproducibility remains a central challenge for CNT-based gas sensors because device performance is strongly affected by the CNT source, chirality, metallic/semiconducting fraction, defect density, dispersion quality, network density, film thickness, electrode contact, functionalization uniformity, and environmental history. Even when the same sensing material is used, variations in CNT percolation networks can produce large differences in baseline resistance, noise level, response magnitude, and recovery behavior. Peptide-functionalized CNT chemiresistors, for example, showed that a lower CNT density can slightly improve sensitivity but also increases noise and device-to-device variation, whereas an intermediate CNT density provides a more favorable balance between accessible receptor sites and stable charge transport [38].

Long-term stability should also include mechanical reliability in flexible and wearable devices. CNT films on polymer, paper, or textile substrates can retain a gas response under bending, stretching, or twisting; however, non-uniform film assembly can generate local stress concentration, random cracking, unstable percolation pathways, and baseline drift during repeated deformation. Printed ion-gel CNT-TFT H_2_S sensors, for example, showed stable operation after 10,000 bending cycles at a 5 mm radius while maintaining ultralow-power transistor-mode amplification [179]. Such bending durability is a form of long-term stability because it determines whether a flexible sensor can maintain calibration during repeated use.

A minimal reporting protocol is needed to make CNT gas-sensor studies more comparable, reproducible, and practically assessable. Since CNT sensor performance is strongly influenced by the CNT source, defect density, network density, functionalization uniformity, contact geometry, relative humidity, and recovery protocol, these parameters should be reported together with the sensing response rather than treated as secondary information. Table 9 summarizes the essential reporting categories, the minimum information to report, and the reason each item is needed. In addition to the response and LOD, future studies should clearly report the CNT material properties, synthesis and purification history, functionalization and sensing-layer fabrication conditions, substrate and electrode information, channel geometry, baseline current or resistance, response definition and sign convention, sensitivity and LOD calculation methods, gas concentration range, carrier gas, flow rate or chamber volume, exposure/recovery protocol, operating temperature, relative humidity, recovery method, selectivity and interference tests, repeated-cycle data, long-term drift, device-to-device or batch-to-batch statistics, mechanical durability tests for flexible devices, and power consumption of the sensing layer, heater or light source, readout circuit, and wireless module. When device statistics, long-term stability, or other key parameters are unavailable, their absence should be explicitly acknowledged as a reporting gap.

### 8.5. Power Consumption and Low-Temperature Operation

Power consumption is a decisive parameter for continuous, portable, wireless, and wearable sensing. CNT sensors are attractive because they can often operate at room temperature or at lower temperatures than conventional metal-oxide sensors. Nevertheless, many CNT hybrids still require thermal, optical, or electrical activation to accelerate recovery, stabilize the baseline, or activate surface reactions. Thus, the relevant metric is not only the power used by the sensing channel but also the total power required for recovery, heating, readout, compensation, and communication.

Several low-power CNT sensor architectures illustrate different trade-offs. Acid-functionalized MWCNT hydrogen sensors detected 0.05% H_2_ at room temperature and reduced the recovery time from 190 s for pristine MWCNTs to 100 s after functionalization, showing that chemical activation can improve low-temperature H_2_ sensing without noble-metal catalysts [148]. In MEMS-based Au-CNT hydrogen sensors, a porous Au-CNT film increased the active surface area while the micro-hotplate platform localized heating; the device consumed 72 mW at 2.5 V and operated at approximately 300 °C [71]. Although this power is higher than self-heated CNT devices, the MEMS platform shows how thermal localization reduces the energy penalty relative to conventional heated substrates.

For ultralow-power operation, self-heated or transistor-mode architectures are more attractive. Suspended CNT sensors used the nanotube itself as a nanoscale heating element and recovered from NO_2_ exposure at only 2.9 µW, demonstrating that self-heating can preserve the low-power advantage of CNTs while solving the recovery bottleneck [72]. Printed ion-gel CNT-TFTs achieved extremely high H_2_S response amplification at room temperature with a working power consumption of 1.03 nW under 2 ppm H_2_S, fast response/recovery times of 10/26 s, and stable bending performance [179]. Filament-heater-integrated transparent CNT sensors further showed a dynamic NO_2_ response at 6.15 µW and complete pulse recovery below 1 ms at 30.8 µW [190]. These examples indicate that studies on future low-power CNT gas sensors should report the power-normalized sensitivity, duty-cycle operation, recovery energy, and total system power rather than only the sensing-layer bias.

### 8.6. System-Level Integration

Future CNT gas sensors should be developed as complete sensing systems rather than as isolated sensing films. Material-level advances such as ternary and quaternary composites, TMD/CNT heterojunctions, MOF/CNT interfaces, and oxide/CNT hybrids must be coupled with signal acquisition, drift compensation, pattern recognition, power management, wireless communication, and user-oriented readout. This system-level perspective is particularly important for flexible and wearable sensors, where selectivity, humidity tolerance, mechanical reliability, and low-power operation must be maintained simultaneously under variable ambient conditions.

The WO_3_/CNT integrated intelligent sensing system provides a recent example of this transition. In that study, WO_3_ nanoparticles supplied NO_2_-reactive adsorption sites, CNTs improved conductivity and flexibility, and the sensor was coupled with an integrated intelligent sensing system for signal acquisition, processing, wireless transmission, and mobile display [189]. Such demonstrations show that CNT-based gas sensors are moving from material-focused devices toward on-site monitoring platforms in which the sensing layer, flexible substrate, readout circuit, communication module, and user interface are designed together.

Recent algorithm-centered studies further indicate that system-level reliability cannot be achieved by hardware optimization alone. A unified deep-learning framework for smart gas sensing combined multi-task learning, transfer learning, domain adaptation, and SHAP-based interpretability to predict the sensor status, gas identity, and concentration from transient responses while improving robustness against sensor drift [73]. Although this framework was demonstrated using a general gas-sensor array, its concepts are directly relevant to CNT-based electronic noses because CNT networks commonly suffer from cross-sensitivity, baseline drift, and device-to-device variability. Similarly, recent reviews of AI-enabled MOS gas sensors show that classical machine learning and deep neural networks can compensate for poor intrinsic selectivity and support applications in smart factories, smart agriculture, healthcare, and smart homes [74].

CNT-specific system demonstrations also support this direction. A machine-learning-optimized CNT sensor platform used a hierarchical 16-element CNT array, multifunctional CNT surface chemistries, and a CNN/LSTM/attention-based processing pipeline with a Synthetic Signature Subtraction algorithm to reduce cross-reactivity during simultaneous monitoring of environmental contaminants [191]. The reported system achieved 94.2% overall classification accuracy, detection limits of 0.05–1.2 ppb, 87.3–96.8% accuracy in complex environmental matrices, and real-time wireless operation at an average power consumption of 315 mW [191]. Although further independent validation and standardization are still necessary, this example illustrates how CNT sensor arrays can be combined with embedded electronics and machine learning to move toward field-deployable multi-analyte monitoring.

Another promising route is to increase sensing dimensionality electronically rather than by adding many different sensing materials. Electrostatically reconfigurable CNT field-effect transistor sensing units use the bottom-gate voltage to modulate the chemical potential of the top-gate sensing layer, thereby tuning gas adsorption and charge-transfer behavior without changing the material composition [192]. This approach generated distinct gate-dependent response patterns for SO_2_, NO_2_, and O_2_, enabled classification accuracies above 90% within 3 min, and was proposed as a back-end-of-line-compatible path toward CMOS-integrated artificial olfaction [192]. Reconfigurable CNT transistor arrays therefore provide a useful bridge between material-level CNT sensing and scalable intelligent sensing hardware.

Overall, the next stage of CNT gas-sensor development should emphasize co-design across four levels: (i) functional CNT interfaces that provide analyte-dependent interaction pathways, (ii) device architectures that support low-noise, low-power, and mechanically robust transduction, (iii) array or reconfigurable designs that generate high-dimensional response fingerprints, and (iv) algorithmic and communication layers that enable real-time classification, quantification, drift correction, and user feedback. Reporting these system-level features together with conventional response metrics will make CNT gas sensors more comparable, reproducible, and relevant to practical deployment. To clarify these system-level directions, Table 10 summarizes representative strategies for deployable CNT-based gas sensors, including integrated sensing modules, AI-assisted gas recognition frameworks, machine-learning-optimized CNT sensor arrays, and reconfigurable CNT transistor platforms.

## 9. Conclusions

Carbon nanotubes (CNTs) provide a versatile platform for next-generation gas sensors because their one-dimensional structure, large accessible surface area, high electrical conductivity, and tunable electronic properties allow gas adsorption to be converted into measurable electrical signals. In pristine CNT networks, charge transfer between adsorbed gas molecules and the nanotube surface enables room-temperature detection of strongly interacting gases, such as NO_2_ and NH_3_. However, pristine CNTs generally suffer from limited selectivity, weak response to low-reactivity molecules, slow or incomplete recovery, and sensitivity to environmental factors such as humidity. The development of CNT gas sensors has therefore shifted from simple chemiresistive nanotube films toward engineered hybrid interfaces and device architectures.

Functionalization is the key strategy for expanding the sensing capability of CNTs. Catalytic noble metals such as Pd, Pt, Au, and Ag promote dissociation or selective adsorption of target gases, enabling sensitive detection of H_2_ and other weakly adsorbing molecules. Metal oxides including SnO_2_, ZnO, TiO_2_, WO_3_, CuO, and Fe_2_O_3_ introduce redox-active surfaces and p–n or Schottky junctions that amplify resistance modulation. Conducting polymers such as PANI, PPy, and PEDOT provide gas-specific interactions, doping/dedoping chemistry, and flexibility for room-temperature operation. Graphene, rGO, and other two-dimensional materials further improve charge transport, mechanical stability, and interfacial tuning. Through these strategies, CNT-based sensors have been extended to a broad range of analytes, including NH_3_, NO_2_, H_2_, CO, H_2_S, CO_2_, NO, and volatile organic compounds.

Device design has also become increasingly important. Chemiresistors and field-effect transistors remain the most widely used platforms because of their simple structure, low cost, and compatibility with miniaturized electronics. At the same time, Schottky-contact, diode, ionization, capacitive, resonant, and self-heating architectures provide additional routes to improve sensitivity, reduce power consumption, accelerate recovery, or detect gases with low adsorption energy. The integration of CNT films onto flexible substrates such as polymers, paper, textiles, and masks has further expanded their potential for wearable exposure monitoring, breath analysis, and point-of-care diagnostics. These advances show that CNTs should be considered not only as sensing materials but also as conductive scaffolds, flexible electrodes, interfacial transducers, and components of integrated sensing systems.

Despite this progress, several challenges still limit practical deployment. Reproducible synthesis and large-area deposition of CNT networks remain difficult because sensor performance is strongly affected by the CNT type, chirality, purity, defect density, alignment, film thickness, and junction resistance. Selectivity is still a major issue in complex gas mixtures, particularly under variable humidity. Strong adsorption on defects, functional groups, polymers, or catalytic sites can improve sensitivity but often causes slow recovery and baseline drift. Long-term stability, calibration reliability, device-to-device variation, and standardized testing protocols also require further attention. Future studies should therefore move beyond reporting high response values under ideal laboratory conditions and instead evaluate sensors under realistic humidity, temperature, interfering-gas, and long-term cycling environments.

Overall, CNT-based gas sensors have evolved from pristine nanotube chemiresistors into multifunctional hybrid platforms that combine carbon nanostructures, receptor chemistry, catalytic nanoparticles, semiconducting oxides, conducting polymers, two-dimensional materials, flexible substrates, and signal-processing methods. The most promising future devices will likely rely on deliberately integrated mechanisms rather than a single sensing pathway, with the material synthesis, functional interface design, device geometry, environmental compensation, power budget, recovery strategy, and data interpretation optimized together as one complete system. If reproducibility, selectivity, humidity tolerance, recovery, and standardization can be addressed, CNT-based gas sensors can make substantial contributions to portable environmental monitoring, industrial safety, hydrogen-infrastructure protection, smart packaging, wearable exposure assessment, and non-invasive health diagnostics.

## Figures and Tables

**Table 1:** Representative seminal contributions and paradigm-shifting advances in CNT-based gas sensors.

Single-tube CNT transistor gas sensing	Seminal NH_3_ and NO_2_ detection using individual semiconducting SWCNT transistors	Demonstrated that trace molecular adsorption can strongly modulate CNT conductance at room temperature.	Established CNTs as highly sensitive, low-power electronic transducers for gas sensing and provided the foundation for charge-transfer-based CNT chemiresistors.	[14]
CNT-network chemiresistors and chemically diverse arrays	Functionalized SWCNT network chemiresistors and array-type CNT sensors	Shifted CNT gas sensing from single-tube proof-of-concept devices to scalable network films and partially selective sensor arrays.	Enabled practical device layouts, chemical diversity, multichannel responses, and pattern-recognition approaches for improved selectivity.	[15,16]
CNT ionization gas sensors	Vertically aligned CNT nanotip ionization sensors for fingerprint-like gas identification	Introduced a non-adsorption-based CNT sensing principle using field-enhanced ionization rather than simple resistance modulation.	Expanded CNT sensing to weakly adsorbing or inert gases and diversified CNT transduction beyond chemiresistive devices.	[17,18]
Noble-metal-decorated CNT hydrogen sensors	Pd/Pt-functionalized CNT sensors, PdAu-PMP composites, Pd/Cr/Pd SWCNT diodes, and Pd/SWCNT Schottky contacts	Showed that catalytic dissociation, spillover, PdH_x_ formation, and metal/CNT work-function or Schottky-barrier modulation can overcome the weak intrinsic CNT-H_2_ interaction.	Established catalytic metal/CNT interfaces as a general route for detecting weakly interacting gases and for engineering fast, selective room-temperature H_2_ sensors.	[19,20,21,22,23,24,25,26,27,28,29]
Molecular receptor and polymer-functionalized CNT sensors	PANI/CNT, PPy/CNT, PEDOT/CNT, polymer-wrapped SWCNT, peptide-functionalized CNT, metalloreceptor-SWCNT, and CuPc/SWCNT sensors	Demonstrated that selectivity can be engineered through receptor chemistry, acid–base interaction, polymer doping/dedoping, swelling, and molecular recognition.	Moved CNT gas sensing beyond nonspecific donor/acceptor charge transfer toward chemically selective interface design and analyte-specific recognition.	[30,31,32,33,34,35,36,37,38,39,40,41]
CNT/oxide, CNT/TMD, and CNT/MOF hybrid interfaces	Metal-oxide/CNT hybrids, MOF/MWCNT composites, CNT/TMD core–shell structures, WSe_2_/MWCNT, CNTs@MoS_2_, and MWCNTs-InSe heterostructures	Introduced heterojunction modulation, surface-oxygen reactions, molecular sieving, adsorption-site engineering, and humidity-tolerant hybrid interfaces.	Expanded CNT sensors toward higher selectivity, lower operating temperature, improved humidity tolerance, and mechanism-guided hybrid-interface design.	[42,43,44,45,46,47,48,49,50,51,52,53,54,55,56,57]
Flexible, wearable, and self-powered CNT sensing platforms	Mask-integrated CNT/PANI sensors, CNT fibers, textile-compatible CNT composites, self-powered SWCNT/Si systems, wireless polymer/MWCNT arrays, MoS2-SWCNT films, and PVA/MWCNT fabrics	Moved CNT gas sensors from rigid laboratory devices toward mechanically compliant, wearable, wireless, textile, and autonomous sensing formats.	Linked CNT sensing materials with flexible substrates, user-oriented monitoring, wireless readout, mechanical durability, and low-power operation.	[28,29,58,59,60,61,62,63,64,65,66,67,68,69]
System-level and intelligent CNT sensing systems	WO_3_/CNT integrated intelligent sensing system, AI-assisted gas-sensor frameworks, ML-optimized CNT arrays, and reconfigurable CNT transistor arrays	Reframed CNT gas sensors as deployable systems combining sensing films, electronics, data processing, wireless communication, and intelligent pattern recognition.	Established a future direction toward field-deployable multi-analyte monitoring, drift compensation, artificial olfaction, and standardized system-level validation.	[70,71,72,73,74]

**Table 2:** Comparison of the principal CNT synthesis and post-treatment methods relevant to gas-sensor fabrication.

Method	Typical Conditions	Product Features	Advantages for Sensors	Limitations/Sensing-Related Concerns	Refs.
Arc discharge	Graphite electrodes; inert gas; high-current arc	Highly crystalline CNTs; low defect density; relatively inert sidewalls	Stable electrical transport, low intrinsic noise, and useful benchmark material for evaluating intrinsic CNT responses	Limited morphology control and scalability; low density of adsorption/anchoring sites may require additional functionalization for weakly interacting gases	[88,89,90]
Laser ablation	Laser-vaporized graphite-catalyst target; high temperature	High-purity, SWCNT-rich products; low defect density; relatively narrow structural distribution	Useful for high-quality reference materials and studies requiring stable CNT electronic properties	Costly and low throughput; limited direct device integration; relatively inert surfaces may reduce response without receptor or catalyst modification	[90,91]
CVD	Hydrocarbon precursor with Fe/Co/Ni/Mo catalyst; typically 550–1000 °C	Controllable density, alignment, length, and patterning; more defects and residual catalyst species than arc/laser CNTs	Scalable and compatible with direct device growth; defects, open ends, and residual catalysts can provide adsorption, catalytic, or anchoring sites	More structural variation; residual catalysts and nonuniform growth can increase baseline drift, hysteresis, humidity sensitivity, and device-to-device variation	[90,92,93,94]
Purification/acid treatment	Oxidative purification, acid washing, sonication, annealing, or plasma treatment after synthesis	Removes amorphous carbon and excess catalysts; introduces oxygen-containing groups such as carboxyl, hydroxyl, and carbonyl groups	Improves dispersion and film formation; creates anchoring sites for metals, oxides, polymers, MOFs, or biomolecular receptors; can enhance charge transfer	Excessive oxidation can damage CNT conductivity, introduce nonuniform functional groups, increase water adsorption, and reduce reproducibility	[94,95,96]

**Table 4:** Recommended performance metrics for CNT-based gas sensors intended for comparison across studies.

Metric	Recommended Definition	Why It Matters	Common Pitfalls
Response	Relative change in resistance, conductance, current, capacitance, frequency, or optical signal at a specified concentration	Allows comparison of signal amplitude	Different formulas can invert sign or exaggerate response; always report baseline
Sensitivity	Slope of calibration curve within a defined linear or quasi-linear range	Determines quantitative resolution	Single-point response is often mislabeled as sensitivity
LOD	Minimum reliably detectable concentration, preferably from noise and calibration slope	Defines trace-detection capability	LOD extrapolated beyond the measured range can be misleading
Response/recovery time	Time to reach 90% of response or return toward baseline under stated flow conditions	Captures adsorption/desorption kinetics	Static and dynamic chambers are not directly comparable
Selectivity	Target response relative to relevant interferents and mixtures	Determines real-use utility	Testing only dry single gases overestimates performance
Humidity stability	Response and baseline under controlled RH values	Critical for breath, wearable, and environmental sensing	RH often omitted or not stabilized
Repeatability and drift	Cycle-to-cycle variation and long-term baseline/sensitivity change	Predicts calibration lifetime	Short tests do not represent shelf life or field use
Power consumption	Power used by sensing element, heater/light source, readout, and communication	Required for IoT and wearable nodes	Only the sensing layer is sometimes counted while peripheral power is ignored

**Table 5:** Representative performance of functionalized CNT and CNT/graphene gas sensors selected from Section 5.

Sensor/Material	Gas	Conditions	Key Performance	Main Role	Ref.
Ag-Cu_2_O/CNT	NH_3_	RT; 100 ppm	Response 76.3; 193 s	Ag/oxide/CNT sensitization	[127]
CNT-templated In_2_O_3_	HCHO	300 °C; 100 ppm	Response 63.5; ~30× compact film	Open 1D oxide network	[128]
PANI/MWCNT	NH_3_	RT; 2–10 ppm	15.5% at 2 ppm; 32% at 10 ppm	Thin PANI shell	[30]
PANI/CNT fibers	NO_2_/NH_3_	RT; 50 ppm	5.2 s/1.8 s response; ppb LODs	Interface-tuned polarity	[31]
Flexible PANI/CNT film	NH_3_	RT; 0.2–50 ppm	85/20 s; bending-stable	Flexible polymer/CNT network	[32]
MWCNT-PEDOT/PANI	NH_3_	RT + thermal/DC	Recovery 48 h -> 20 min	Assisted desorption	[33]
SWCNT/PPy	NO_2_	RT	~10× sensitivity vs. PPy	PPy sites + CNT transport	[34]
Pd&PEDOT@CNT	H_2_/NH_3_	RT	Opposite resistance directions	Dual-gas discrimination	[35]
PANI network/PET	NH_3_	RT; flexible	Improved vs. particle-like PANI	Polymer morphology control	[36]
Polymer-coated SWCNT	IPA	RT; 20–100 ppm	LOD 19.3 ppm; linear response	Polymer receptor layer	[37]
3D graphene/CNT	NO_2_	RT; 0.01 ppm	9.1%; 6/10 s	Porous 1D/2D network	[120]
rGO-MWCNT-SnO_2_	NO_2_	RT; 5 ppm	243%; 13/87 s	Ternary junctions	[155]
Sn-TiO_2_/rGO/CNT	NH_3_	RT; 250 ppm	85.9%; 120/80 s	Oxide/carbon interface	[156]
Pt/graphene-like CNT	H_2_	RT; 4%	42.8%; LOD 0.04%	Pt catalytic sites	[130]
GNS-grafted DWCNT	Ethanol	RT	~3× response vs. DWCNT	Larger adsorption surface	[157]
GO-CNT aerogel	Chlorobenzene	RT; 5 ppm	~95%; LOD ~90 ppb	Oxygenated GO sites	[158]
CNT/rGO-Am	NH_3_	RT; 25 ppm	70%; LOD < 0.2 ppb	Amine-rich junction	[159]
MWCNT/rGO-Co_3_O_4_	Ethanol	180 °C; 100 ppm	12,610%; 50/20 s	Mesoporous oxide synergy	[131]
CeO_2_/rGO/CNT	LPG	RT; 400 ppm	42% response	CeO_2_/carbon scaffold	[129]
Amine-GO/CNT	TNT vapor	RT	LOD 0.01 ppm	Functional GO/CNT interface	[132]

Note: Response values are listed as reported in the original articles and should not be directly ranked because response definitions, gas concentrations, operating conditions, humidity levels, and device configurations differ among studies.

**Table 6:** Representative performance of CNT-based NH_3_, NO_2_, H_2_, H_2_S, and CO_2_ sensors selected from Section 6.

Sensor/Material	Gas	Conditions	Response/LOD/Kinetics	Selectivity/Stability/Durability; Dominant Mechanism	Ref.
ZIF-8/CNT	NH_3_	RT; 25–250 ppm; RH 45–70%	LOD 208 ppb; response/recovery NR	Humidity-stable MOF/CNT interface; ZIF-8 preconcentration and humidity-screening mechanism	[55]
FO-N-PA/SWCNT	NH_3_	RT; 1–35 ppm	21.3% at 1 ppm; 73.3% at 35 ppm; kinetics NR	Semiconducting-SWCNT enrichment and Schiff-base wrapping improve NH_3_ charge-transfer selectivity; stability NR	[40]
PANI@Cu@MWCNT	NH_3_	RT; 100 ppm	116% max.; 10/13 s response/recovery	PANI/Cu/MWCNT synergy; acid–base interaction and conductive polymer doping/dedoping; repeatability NR	[162]
WO_3_/CNT	NH_3_	RT	Response depends on CNT loading; LOD/kinetics NR	WO_3_/CNT Schottky- or heterojunction-mediated modulation; stability NR	[56]
CNTs@MoS_2_	NH_3_	RT; 50–500 ppm	94% at 500 ppm; 36/68 s; LOD 10.33 ppm	CNT/TMD heterojunction and adsorption-site engineering; selectivity reported; long-term stability NR	[57]
In-MWCNT	NH_3_	RT; 10 ppm	144% vs. 117% for pristine MWCNT; kinetics/LOD NR	Indium decoration creates active adsorption sites and enhanced charge transfer; stability NR	[163]
CuPc/SWCNT	NH_3_	RT; 40 ppm	56% response enhancement; kinetics/LOD NR	π–π molecular functionalization improves NH_3_ interaction; repeatability/stability NR	[41]
Granulated CNF	NO_2_	25 ± 2 °C; 1–500 ppm	5.1% at 10 ppm; kinetics/LOD NR	Low-cost carbon platform; humidity-sensitive response highlights need for RH control	[167]
3D SiO_2_@MWCNT	NO_2_	RT + UV; 1 ppm	82.6%; recovery ~44 s; LOD NR	3D surface exposure and UV-assisted desorption improve recovery; stability NR	[169]
N-doped SWCNT film	NO_2_	RT; 0.01 V	LOD 500 ppb; response/recovery NR	Pyridinic-N adsorption sites; bending-stable flexible film; long-term drift NR	[145]
Oxy-SWCNT/cellulose	NO_2_	RT	LOD 0.43 ppb; kinetics NR	Cellulose-assisted adsorption in transparent/flexible architecture; selectivity/stability NR	[146]
SnO_2_ heater/CNT	NO_2_	RT; 0.5–20 ppm	40.69% at 20 ppm; recovery enabled by heater; 50 cycles	Integrated microheater supports repeated recovery; oxide/CNT interfacial transduction	[171]
en-APTAS/CNT-ZnO	NO_2_	RT + recovery pulse	60% response increase; 1 ms recovery pulse	Amine membrane and memristive recovery strategy; pulse-assisted reversibility; long-term stability NR	[172]
CMC/SDS-SWCNT	NO_2_	150 °C; 2 ppm	78.6% (CMC); 62.5% (SDS); kinetics/LOD NR	Surfactant-controlled printed CNT films; selectivity/stability NR	[173]
PdAu–PMP composite	H_2_	RT; 10 ppm–100%	LOD ~1 ppm; 3/8 s at 50% H_2_	Oxygen-independent Pd/MWCNT@PVP + PdAu alloy mechanism; catalytic dissociation/PdHx and CNT conduction	[27]
Pd/sc-SWCNT + PMMA	H_2_	RT; 1%	Response 285; 10/3 s response/recovery	Controlled monolayer and PMMA coating; Pd-catalyzed H_2_ dissociation and charge/barrier modulation; stability NR	[175]
Functionalized MWCNT/Pd	H_2_	RT; 20–140 ppm	315% at 140 ppm; kinetics/LOD NR	Acid functionalization and Pd contacts enhance H_2_ spillover/contact modulation; selectivity/stability NR	[176]
CNT/TiO_2_ by two-stage DEP	H_2_	RT; 1000 ppm	15.78% vs. <2% for CNT-only; kinetics/LOD NR	Percolation-preserving oxide deposition; CNT/oxide heterointerface enhances response; stability NR	[60]
Pd/Cr/Pd SWCNT diode	H_2_	RT; 5000 ppm	Response 12.56; 22 s response; recovery NR	Schottky-barrier modulation at Pd/SWCNT interface; mechanism-confirming diode design	[28]
Pt/CNT/graphene	H_2_	RT; 10%	26%, increased to 36% after UV; kinetics/LOD NR	Pt catalysis with graphene-assisted transport and UV-assisted activation; stability NR	[62]
Pd/SWCNT Schottky contact	H_2_	RT; 50–2000 ppm	Logarithmic response; kinetics/LOD NR	Pd contact controls Schottky-barrier/work-function modulation; mechanism-focused study	[29]
CuO-SWCNT hybrid	H_2_S	RT; wireless sensor	Selective RT H_2_S response; detailed LOD/kinetics NR	CuO reaction sites and SWCNT transport; CuO-to-CuS chemistry supports selectivity	[44]
CNT/Fe_2_O_3_ film	H_2_S	RT; ppb-level	Flexible ppb-level H_2_S detection; detailed kinetics NR	CNT/oxide hybrid film; oxide surface reaction coupled with CNT conduction; durability NR	[47]
f-MWCNT/Cu	H_2_S	RT; air	~400% ΔR/R_0_; ~5× higher than physically deposited Cu; kinetics temperature-dependent	Oxygenated f-MWCNTs anchor Cu; Cu/CuS-related interaction improves selectivity; self-recovery studied	[177]
Ion-gel CNT-TFT	H_2_S	RT; 2 ppm; 5% RH	4045.5% response; 1565% ppm^−1^; 10/26 s; 1.03 nW	Ion-gel enrichment plus CNT-TFT amplification; excellent selectivity and stable after 10,000 bending cycles	[179]
CNT-CuO-ZnO film	H_2_S	RT; 20 ppb–10 ppm	LOD 20 ppb; 50% at 300 ppb; response stable within ~5 s	Multilevel CuO/ZnO/CNT heterostructure; strong selectivity; 96.8% response retained after bending	[178]
ZnO/CNT	CO_2_	RT	Room-temperature CO_2_ response; LOD/kinetics NR	Oxide/CNT hybrid interface; response attributed to adsorption and interfacial charge modulation; stability NR	[43]
Transferred CNT film/PI	CO_2_	RT; 800 ppm	2.23% sensitivity; immediate response	Uniform flexible CNT film prepared by transfer; favorable response speed/stability; mechanism mainly CNT adsorption	[180]
DLICVD MWCNT thin film	CO_2_	RT	Response 2.1; 98% reproducible; 30.2/49.6 s	CNT morphology and theoretical adsorption support; reproducibility explicitly reported	[182]
PEI-CNT thin film	CO_2_	RT; 200–1000 ppm	~4.2% at 1000 ppm; kinetics NR	PEI amine–CO_2_ interaction/carbamate formation; repeatability/selectivity and flexibility evaluated	[67]
Switchable SWCNT-polymer	CO_2_	Humid conditions	Reversible chemiresistive response; kinetics/LOD NR	Amidine-to-amidinium bicarbonate switching; selective over O_2_ and Ar; humidity-dependent reversible chemistry	[181]
Screen-printed f-CNT/PEI	CO_2_	RT; 300–5000 ppm	Wide-range response; detailed kinetics NR	Scalable printed amine/CNT interface; selectivity and humidity effects evaluated; mechanism proposed	[183]
High-MW PEI/f-CNT	CO_2_	RT; 300–2000 ppm	Robust response over 40 continuous cycles	High-molecular-weight PEI improves aging/thermal stability; screen-printed reproducible PEI/f-CNT composite	[184]
COOH-f-SWCNT/PEI/DAN/Nafion	CO_2_	RT; 500–2500 ppm; variable RH	7.5% at 2500 ppm; 2.67 × 10^−3^ ppm^−1^; 1–3 min dual-mode response/recovery	DAN minimizes drift; Nafion enables humidity-compensated resistive/capacitive dual-mode sensing; repeatability and batch reproducibility reported	[185]

Note: Response values are listed as reported in the original articles. Direct numerical comparison should be made with caution because response definitions, target-gas concentrations, humidity conditions, operating temperatures, device architectures, and readout modes differ among studies.

**Table 7:** Representative flexible and wearable CNT-based sensing platforms selected from Section 7.

Platform/Material	Target/Use	Device Format	Key Performance	Wearable Relevance	Ref.
p-PP/CNT/PANI	NH_3_ + breathing	Sensor on surgical-mask fibers	0.5–70 ppm; 452% at 70 ppm; 93/36 s	Mask-integrated breath monitoring	[64]
Continuous CNT fibers	VOCs, H_2_S, NO	Stitched CNT-fiber textile	~50 ppb VOC LOD; <30 s for most VOCs	Weavable, bend/twist/knot tolerant	[65]
rGO/CNT/NWF e-textile	Wearable strain/pressure platform	Non-woven fabric hybrid network	Stable over 2000 cycles; washable	Gas-permeable substrate concept	[66]
LIG-CoPc/MWCNT	Methanol	Flexible LIG electrode + composite	LOD 165 ppb; 5 s initial response	Low-power flexible VOC sensing	[186]
SWCNT/Si self-powered system	NO_2_	Flexible solar cell + SWCNT sensor	LOD 1 ppm; 500 bending cycles	Battery-free wireless alarm potential	[134]
CNT-based ISE array	Na^+^ sweat sensing	Flexible CNT electrochemical electrode	58 ± 3 mV decade^−1^	Wearable CNT transducer design	[187]
PEI-CNT thin film	CO_2_	Vacuum-filtered CNT film on PI	200–1000 ppm; ~4.2% at 1000 ppm	Flexible CO_2_ monitoring	[67]
MWCNT-coated cotton	CO	Dip-coated cotton fabric	25–100 ppm; 15.2% at 100 ppm	Low-cost textile gas sensor	[68]
Polymer/MWCNT array	NH_3_/NO_2_/toluene	Flexible PI array + BLE smartphone readout	1000 ppm NH_3_/NO_2_ and 10 ppm toluene; 40 °C thermostat; ~210 mW heater	Wireless real-time wearable air-pollution monitoring	[69]
MoS2-SWCNT hybrid film	NO_2_/NH_3_	Transparent hybrid film on PET	54% at 40 ppm NO_2_; 34% at 40 ppm NH_3_; stable after 10^5^ bending cycles	Flexible transparent TMD/CNT chemical sensor	[70]
PVA/MWCNT cotton fabric	Ethanol	Spray-coated fabric chemiresistor	100–500 ppm; LOD 9.17 ppm; 24 +/− 2/29 +/− 3 s; ~13-fold vs. bare MWCNT	Low-cost fabric-based room-temperature ethanol sensor	[188]

Note: PI, polyimide; LIG, laser-induced graphene; ISE, ion-selective electrode; BLE, Bluetooth Low Energy; PET, polyethylene terephthalate.

**Table 8:** Representative humidity-mitigation strategies for CNT-based gas sensors.

Strategy	Mechanistic Role	Representative Example	Advantages	Limitations
Hydrophobic CNT/TMD interface	Suppresses water adsorption and improves interfacial charge transport	WSe_2_/MWCNT NO_2_ sensor stable at 80% RH [52]; WS_2_/MWCNT dual NH_3_/NO sensor at 90% RH [147]	Improves RH tolerance while preserving CNT conductivity	Performance depends on TMD edge sites, CNT ratio, and interface uniformity
Oxide/CNT hybrid interface	Combines oxide adsorption sites with CNT percolation pathways	WO_3_/CNT flexible NO_2_ sensor with integrated on-site monitoring [189]	Useful for high-response NO_2_ sensing and integrated readout	Often still requires optimized operating temperature or calibration
Frequency-domain compensation	Separates moisture-sensitive impedance components from gas response	Printed CNT devices with 1 MHz humidity compensation [143]	No extra RH sensor required; compatible with low-cost nodes	Requires frequency-resolved readout and gas-specific calibration curves
Localized microheating or pulsed heating	Promotes desorption and reduces water-induced baseline drift	Filament-heater-integrated low-power transparent CNT sensor [190]	Improves recovery and humidity robustness	Adds device complexity and power budget
Standardized RH reporting	Enables fair comparison under realistic environments	RH-dependent response curves, RH cycling, and baseline-drift tests	Improves reproducibility and field relevance	Requires longer test protocols and additional calibration

**Table 9:** Minimal reporting standards recommended for CNT-based gas-sensor studies.

Reporting Category	Minimum Information to Report	Why It Is Needed
CNT material information	CNT type and source; synthesis method; purification or acid-treatment history; purity; diameter/length range; metallic/semiconducting fraction when relevant; defect or functional-group information; residual catalyst information if available.	CNT chirality, defect density, residual catalyst, and surface chemistry strongly affect baseline resistance, response, recovery, and reproducibility.
Functionalization and sensing layer	Functional material; loading amount; deposition or coating method; dispersion conditions; film thickness or areal density; network density; receptor/catalyst distribution; post-treatment or annealing conditions.	Functionalization determines the dominant sensing mechanism, selectivity, humidity tolerance, recovery behavior, and long-term stability.
Device architecture	Device type; substrate; electrode material; channel length/width; contact geometry; baseline current or resistance; number of devices tested.	Device geometry and contact resistance can dominate CNT sensor response, especially in network, FET, and contact-engineered devices.
Gas exposure conditions	Target-gas concentration range; carrier gas; background oxygen condition; chamber type or volume; flow rate or static-exposure condition; exposure and purge times; operating temperature; relative humidity.	Response values are not directly comparable unless the test atmosphere and exposure protocol are specified.
Response definition and calibration	Exact response formula; sign convention; baseline value; sensitivity calculation method; calibration range; LOD calculation method and noise criterion.	CNT sensors use different response formulas and sign conventions; clear definitions prevent misleading comparisons.
Response/recovery kinetics	Response-time criterion; recovery-time criterion; recovery method; purge condition; incomplete recovery or residual baseline shift if present.	Kinetic values depend strongly on the definition and recovery protocol, especially for strongly adsorbed gases.
Selectivity and interference	Interfering gases; interferent concentrations; gas-mixture tests if available; humidity-dependent selectivity; quantitative selectivity factors or response ratios.	Dry single-gas tests often overestimate practical selectivity in real environments containing humidity and mixed gases.
Repeatability and reproducibility	Number of repeated cycles; cycle-to-cycle variation; number of devices; mean +/− standard deviation; device-to-device and batch-to-batch statistics if available.	Device-to-device variation is a major limitation of CNT network sensors and should be reported rather than hidden.
Long-term stability and durability	Storage time; operating time; baseline drift; sensitivity retention; mechanical bending, stretching, or washing tests for flexible devices.	Practical deployment requires stable operation under repeated exposure, storage, and mechanical stress.
Power and system information	Bias voltage/current; sensing-layer power; heater or light-source power; recovery-pulse power; readout-circuit power; wireless-module power if applicable.	Low-power operation is essential for wearable, wireless, and IoT CNT-sensor platforms, but peripheral power is often omitted.

**Table 10:** Representative system-level strategies for deployable CNT-based gas sensors.

System-Level Strategy	Representative Example	Function in Practical Sensing	Key Remaining Issue	Ref.
Integrated sensing module	WO_3_/CNT flexible NO_2_ sensor coupled with an integrated intelligent sensing system	Connects sensing material with real-time acquisition, processing, wireless transmission, and mobile display	Requires validation under long-term field exposure, humidity cycling, and mixed-gas backgrounds	[189]
Unified AI framework	Multi-task deep learning, transfer learning, domain adaptation, and SHAP-based smart gas sensing	Supports gas identity, concentration, sensor-status prediction, drift compensation, and interpretable channel selection	Needs lightweight deployment on embedded hardware and validation using CNT-specific datasets	[73]
AI-assisted gas-sensor roadmap	AI-enabled MOS gas-sensor review covering PCA, LDA, SVM, RF, BP-NN, CNN, and multi-scenario applications	Provides a transferable roadmap for combining cross-sensitive sensors with pattern recognition	CNT studies should report datasets, preprocessing, training/validation splits, and model uncertainty	[74]
ML-optimized CNT array	Sixteen-element CNT array with CNN/LSTM/attention processing and Synthetic Signature Subtraction	Enables multi-analyte classification, cross-reactivity suppression, wireless operation, and field-oriented environmental monitoring	Independent benchmarking and standardized mixed-gas protocols are needed	[191]
Reconfigurable CNT transistor array	Bottom-gate-programmable CNT FET sensing units with gate-dependent SO_2_/NO_2_/O_2_ fingerprints	Generates multiple sensing states electronically without changing sensing materials and supports CMOS-compatible artificial olfaction	Long-term stability, humidity compensation, and array-level calibration must be demonstrated	[192]

## Data Availability

No new data were created or analyzed in this study. Data sharing is not applicable to this article.
